# The influence of working memory capacity and material complexity on the testing effect

**DOI:** 10.3389/fpsyg.2026.1952951

**Published:** 2026-10-30

**Authors:** Xin Lu, Kejia Qu, Yuanlin Sun, Ya Yang

**Affiliations:** 1 College of Educational Science, Hengyang Normal University, Hengyang, Hunan, China; 2 School of Psychology, Liaoning Normal University, Dalian, Liaoning, China

**Keywords:** diminishing-cues retrieval practice, material complexity, retrieval interval, testing effect, working memory capacity

## Abstract

**Introduction:**

This study investigated how working memory capacity and learning-material complexity influence the testing effect and explored strategies to improve learning outcomes for learners with low working memory capacity studying complex materials.

**Methods:**

Four experiments involving fourth-grade primary school students were conducted. Experiments 1a and 1b compared retrieval practice with restudy among learners with high and low working memory capacities using low- and high-complexity materials, respectively. Experiment 2 examined repeated retrieval practice under equally spaced and expanding interval schedules. Experiment 3 examined diminishing-cues retrieval practice.

**Results:**

For low-complexity materials, learners with low working memory capacity performed significantly better following retrieval practice than restudy. For high-complexity materials, this advantage emerged among learners with high working memory capacity. Among learners with low working memory capacity studying complex materials, both repeated-retrieval schedules in Experiment 2 improved post-test performance relative to restudy. In Experiment 3, diminishing-cues retrieval practice outperformed both cue-free retrieval practice and restudy.

**Discussion:**

The findings suggest that material complexity moderates the relationship between working memory capacity and the testing effect. Repeated retrieval practice under the schedules tested and diminishing-cues retrieval practice may support learners with low working memory capacity when studying complex materials.

## 1 Introduction

Testing has historically been viewed as a neutral educational tool, used to support learning and to evaluate or select learners. Recent experimental evidence, however, has challenged this view by showing that testing can enhance long-term memory more effectively than restudying (Roediger and Butler, 2011; Roediger and Karpicke, 2006; Zhang et al., 2008). This benefit is commonly attributed to retrieval practice, the act of recalling previously learned information during a test (Carpenter, 2009). Retrieval practice is now recognized as a powerful learning strategy, with robust effects reported across many studies (Karpicke and Blunt, 2011; Karpicke and Roediger, 2008; McDaniel and Fisher, 1991; Peterson and Wissman, 2018). These effects have been observed across diverse learning materials, including word lists, prose passages, and images (Finn and Roediger, 2011; Ma et al., 2022; Racsmány et al., 2018; Surma et al., 2018; Zheng et al., 2014).

Numerous studies have examined factors that influence the effectiveness of testing (Roediger and Karpicke, 2006; Roediger and Butler, 2011; Smith and Karpicke, 2014). Recent work suggests that learner characteristics, including working memory capacity, can modulate the testing effect (Agarwal et al., 2016; Tse and Pu, 2012; Wiklund-Hörnqvist et al., 2014). Working memory capacity refers to the amount of information that can be temporarily maintained in working memory and is often used as an index of storage capacity (Baddeley and Hitch, 1974). Differences in working memory capacity are also considered an important source of individual differences in cognitive ability (Conway et al., 2002). Together, these findings provide theoretical and empirical support for examining how learning strategies operate across learners with different cognitive characteristics (Agarwal et al., 2016; Tse and Pu, 2012; Wiklund-Hörnqvist et al., 2014).

Existing studies on the role of working memory capacity in the testing effect have yielded mixed findings. Some studies have reported no significant influence of working memory capacity on the testing effect (Minear et al., 2018; Tse and Pu, 2012; Wiklund-Hörnqvist et al., 2014). In contrast, Agarwal et al. (2016) found that working memory capacity did modulate the testing effect. Using relatively low-complexity factual knowledge as the learning material, they showed that learners with lower working memory capacity benefited more from testing than learners with higher working memory capacity. These inconsistent findings may reflect insufficient attention to material difficulty as a factor shaping the relationship between working memory capacity and the testing effect (Agarwal et al., 2016; Tse and Pu, 2012; Wiklund-Hörnqvist et al., 2014).

Several studies suggest a close relationship between working memory capacity and material difficulty (Li and Zhou, 2006; Song and Chen, 2010). The cognitive resources available to learners with different working memory capacities may be constrained by the complexity of the learning materials (Song and Chen, 2010). Thus, studies examining the role of working memory capacity in retrieval practice should also consider material complexity. Learners with higher working memory capacity may have sufficient cognitive resources to support learning. When materials are low in complexity, both retrieval practice and restudy may produce positive outcomes, leaving little observable difference between the two strategies and reducing the likelihood of a significant testing effect. By contrast, learners with lower working memory capacity may have more limited cognitive resources and may therefore depend more strongly on effective learning strategies, even for less complex materials. For these learners, retrieval practice may provide additional support under cognitive-resource constraints and thereby improve learning outcomes.

A resource-based account provides a further framework for understanding this interaction between working memory capacity and material demands. Zheng et al. (2023) proposed a modified source of activation confusion (SAC) model in which retrieval practice involves both a retrieval-attempt process and a post-retrieval re-encoding process, and both processes draw on working memory resources. From this perspective, the benefit of retrieval practice depends on whether the learner has sufficient resources to support both processes under the demands imposed by the material. This account offers a possible explanation for why lower-WMC learners may benefit from retrieval practice when material demands are relatively modest but may benefit less when complex materials consume more of their available resources. It does not, however, predict every nonsignificant comparison in the present study, and the resource-depletion and re-encoding mechanisms were not directly measured here.

Building on this rationale, the present study examined the testing effect for materials of different complexity among learners with varying working memory capacity. We focused on fourth-grade primary school students and used calculation rules for figure areas as the learning materials. Because working memory capacity includes verbal and visuospatial components, and visuospatial working memory is particularly relevant to learning mathematical graphs (Mammarella et al., 2013; Passolunghi and Mammarella, 2012; Verstijnen et al., 1998), the present study focused specifically on learners’ visuospatial working memory capacity.

Experiment 1 comprised two parallel sub-experiments, Experiments 1a and 1b. Experiment 1a examined the testing effect for low-complexity materials among learners with high and low working memory capacity. We hypothesized that, for low-complexity materials, a testing effect would be observed only among learners with lower working memory capacity. Experiment 1b examined the testing effect for high-complexity materials in the same learner groups. Because high-complexity materials may impose greater cognitive load on learners with lower working memory capacity, these learners may have insufficient resources to benefit from retrieval practice. We therefore hypothesized that, for high-complexity materials, a testing effect would be observed only among learners with higher working memory capacity.

If the hypotheses of Experiment 1 are supported, further research will be needed to determine how retrieval practice can be used to improve learning of high-complexity problem-solving tasks among learners with lower working memory capacity. Such tasks are central to student learning across many educational domains. Identifying ways to support learners with lower working memory capacity in acquiring complex materials, while promoting effective use of testing strategies, therefore has important practical implications.

Learners with lower working memory capacity often face difficulties when learning complex materials because such materials place greater demands on cognitive resources. These demands may leave insufficient resources for learners to use testing strategies effectively, leading to weaker learning outcomes. Consistent with this view, Leahy and Sweller (2019) showed that complex cognitive tasks can reduce available working memory resources and diminish the testing effect. They further proposed that introducing intervals between cognitive activities may help restore depleted working memory resources. By varying the intervals between retrieval-practice activities, they found that longer intervals could more effectively support the recovery of working memory resources.

Building on this evidence, Experiment 2 examined whether repeated retrieval practice under specified interval schedules could enhance the testing effect for high-complexity materials among learners with lower working memory capacity. We hypothesized that repeated retrieval, particularly under the equally spaced schedule, would enhance the benefits of retrieval practice for these learners.

Another possible reason why learners with lower working memory capacity may struggle to use testing strategies effectively for complex materials is low initial retrieval success. Complex materials often involve multiple interconnected knowledge elements that constrain and support one another. Learners with lower working memory capacity may therefore have difficulty achieving successful initial retrieval and identifying relations among these elements. This difficulty may weaken the testing effect. Increasing initial retrieval success may be one possible way to improve the effectiveness of testing strategies, but this possibility requires direct process measures.

Fiechter and Benjamin (2018) reported that diminishing-cues retrieval practice can enhance later memory performance and strengthen the association between retrieval cues and target information, particularly when conventional retrieval practice is less effective.

Building on these findings, Experiment 3 examined whether diminishing-cues retrieval practice could enhance the testing effect for high-complexity materials among learners with lower working memory capacity. We hypothesized that, for these learners, diminishing-cues retrieval practice would improve learning outcomes.

## 2 Experiment 1

### 2.1 Experiment 1a: the influence of working memory capacity on the testing effect in learners processing low-complexity materials

#### 2.1.1 Method

##### 2.1.1.1 Participants

G*Power 3.1.9.2 software (Faul et al., 2007) was used to estimate the sample size (with a minimum required number of participants per group no less than 18; power >0.80; effect size of $\eta_{p}^{2}$ = 0.25). A total of 338 fourth-grade primary school students were initially screened, all with intelligence scores within the average range (90–109). The final analysis sample comprised 90 students: 43 with low working memory capacity (24 girls, 19 boys; average age 10.21 years, SD = 0.51) and 47 with high working memory capacity (18 girls, 29 boys; average age 10.34 years, SD = 0.48). The low-working-memory-capacity group had WMC scores ranging from 2.5 to 3.0, whereas the high-working-memory-capacity group had scores ranging from 4.5 to 5.5.

In the present study, all primary school students who participated in the experiment did so with the explicit written consent of their parents or legal guardians. Prior to the commencement of the study, the research team provided detailed information to the parents or legal guardians of each participant regarding the purpose of the study, the experimental procedures, the anticipated duration, and any potential risks and benefits. This ensured that they fully understood and voluntarily agreed to their child’s participation in this research. Additionally, the study has undergone rigorous ethical review and has been formally approved by the relevant ethics committee, with the ethical review number “LL2023004.” Throughout the entire research process, the research team strictly adhered to ethical guidelines to ensure that the rights of all participants were fully protected, and that the experimental procedures were safe, transparent, and in compliance with the ethical standards of psychological research.

##### 2.1.1.2 Design

A between-subjects design was employed, with a 2 (learning method: retrieval practice vs. restudy) × 2 (working memory level: high vs. low) factorial design. The dependent variable was the final test score.

##### 2.1.1.3 Materials

The purpose of this experiment was to facilitate students’ learning by providing specific examples, enabling them to grasp the calculation rules for parallelograms, triangles, trapezoids, and diamonds. To ensure that all participants had no prior knowledge of the calculation methods for these shapes, it was necessary to select participants who were unfamiliar with those specific formulas.

*Pre-test*. The pretest materials consisted of six basic graphic area calculation formulas, including rectangles, squares, parallelograms, triangles, trapezoids, and diamonds. Mastery of basic knowledge, specifically the area formulas for rectangles and squares was necessary for smooth progression in the experiment. Therefore, students included as participants were those who correctly provided formulas for rectangles and squares but incorrectly provided formulas for parallelograms, triangles, trapezoids, and diamonds.

Corsi block-tapping test. Visual–spatial working memory capacity was assessed with a computerized mouse-click version of the Corsi block-tapping task. The task included seven span lengths (3–9 blocks), with three trials at each span length and a maximum of 21 scheduled trials. On each trial, squares changed from a neutral color (e.g., gray) to green in a randomized sequence, and participants reproduced the sequence by clicking the locations in the order presented. The task was discontinued when fewer than two of the three trials at a given span length were reproduced completely correctly. The primary spatial working-memory span score was calculated as the highest completed span minus 0.5, yielding a theoretical range of 2.5–8.5. Trial-level location accuracy was also recorded as a percentage from 0% to 100%, but was not used as the primary grouping index. Learners scoring in the top 27% of the screening sample were categorized as having high working memory capacity, whereas those in the bottom 27% were categorized as having low working memory capacity (Andersson and Lyxell, 2007). Standard Corsi task research supports its use as a measure of visuospatial short-term memory and working memory, but also documents substantial variation in stimulus layout, administration, and scoring (Berch et al., 1998; Kessels et al., 2000). Because the present study used a computerized, mouse-based, self-terminating version, psychometric coefficients from standard versions should not be assumed to apply directly. This adapted version has not undergone independent local psychometric validation.

##### 2.1.1.4 Combined raven’s test (CRT)

The test, revised by Li et al. (1988), was administered solely during participant screening to assess general nonverbal reasoning ability. The CRT contains six sections (A, AB, B, C, D, and E), with 12 items in each section. The test was administered and scored according to the procedures and norms for the revised version; the normative and psychometric information for this version is reported by Li et al. (1988), and students with standardized intelligence scores between 90 and 109 were eligible for inclusion. CRT scores were used only to define this screening criterion and were not entered as covariates or predictors in the subsequent analyses. Restricting the intelligence-score range therefore served as an inclusion criterion and did not statistically control for general intelligence.

*Learning materials*. Materials adapted from Chen et al. (2015) were divided into low- and high-element interactive materials according to the number of elements for task completion.

Low-element interactive material involved combinations of two out of the four basic geometric graphics, resulting in six potential combinations (e.g., trapezoid + diamond). Solving these problems required knowledge of each shape’s area formula and the ability to draw auxiliary lines to separate combined shapes, resulting an element interaction level of 3.

High-element interactive materials included complex geometric combinations, incorporating shaded areas, involving three basic geometric shapes (e.g., trapezoid, triangle, and parallelogram), and trapezoids as the shadow part. Participants needed to master all relevant area formulas, calculate additional dimensions (e.g., the height of a parallelogram), and use auxiliary line for separation, resulting in an element interaction level of 5. Examples of the learning materials and task requirements are provided in the Supplementary materials.

Results indicated a significant difference in average score between high-element interactive materials (*M* = 6.53, SD = 1.21) and low-element interactive materials (*M* = 2.00, SD = 1.06), *t*(38) = 12.56, *p* < 0.001, Cohen’s *d* = 3.49. There was no significant difference in perceived external cognitive load scores between high-element interactive materials (*M* = 1.65, SD = 0.69) and low-element interactive materials (*M* = 1.40, SD = 0.50).

*Test materials*. The test materials mirrored the learning materials, including three problem-solving steps, with one point for each step. The total score was 3 points.

##### 2.1.1.5 Procedure

All students in the screening sample completed the pre-test, including the Corsi block-tapping test, before any participant began the learning phase. After the screening was completed, Corsi scores were ranked within the full screening sample, and students in the top and bottom 27% were classified as having high and low working memory capacity, respectively. The interval between the pre-test and the learning phase was 2 days.

Experiments 1a, 1b, 2, and 3 used non-overlapping participant samples, and each student participated in only one experiment. Therefore, the observations across experiments were independent with respect to repeated participation by the same individuals.

The experiments were conducted in a quiet laboratory. The procedure was as follows:

*Pre-test*: Participants received the pre-test questions presented on A4 paper, accompanied by the instructions: “Please carefully read the questions provided, answer them truthfully, and do your best to provide complete answers.” Participants had 10 min to complete the pre-test.

The Corsi block-tapping test was used to assess the visual–spatial working memory capacity of the participants.

The CRT was administered during screening to evaluate participants’ general nonverbal reasoning ability. Students with standardized scores between 90 and 109 were retained. CRT scores were not included as covariates or predictors in the analyses.

At the end of the learning phase, participants completed the Cognitive Load Scale (CLS), an eight-item self-report questionnaire. Items 1–4 assessed perceived intrinsic cognitive load of the materials, including their complexity and the effort required for learning and problem solving. Items 5–8 assessed perceived external cognitive load associated with the clarity of the explanations, instructions, and material presentation. Each item was rated on a 7-point scale (1 = completely inconsistent, 7 = completely consistent). The CLS was administered immediately after the learning phase as a manipulation check, to evaluate whether the high- and low-element interactive materials elicited the intended difference in perceived cognitive load while producing comparable clarity of instructions and material presentation. The scale assessed subjective cognitive load and was not intended to directly measure working-memory resource depletion or recovery. The full questionnaire is provided in the Supplementary materials.

*Learning phase*: Both groups initially learned a worked examples for 5 min.

Subsequently, the retrieval practice group engaged in retrieval activities for 4 min after a 1-min interval, followed by feedback presented for 1 min. In contrast, the restudy group restudied the worked examples for 5 min.

*Post-test*: Two days after the learning phase, participants in both retrieval practice and restudy groups completed a 10-min assessment evaluating their retained knowledge. The sequence, materials, and timing of the Experiment 1a procedure are summarized in the flowchart below. See Figure 1.

**Figure 1:**
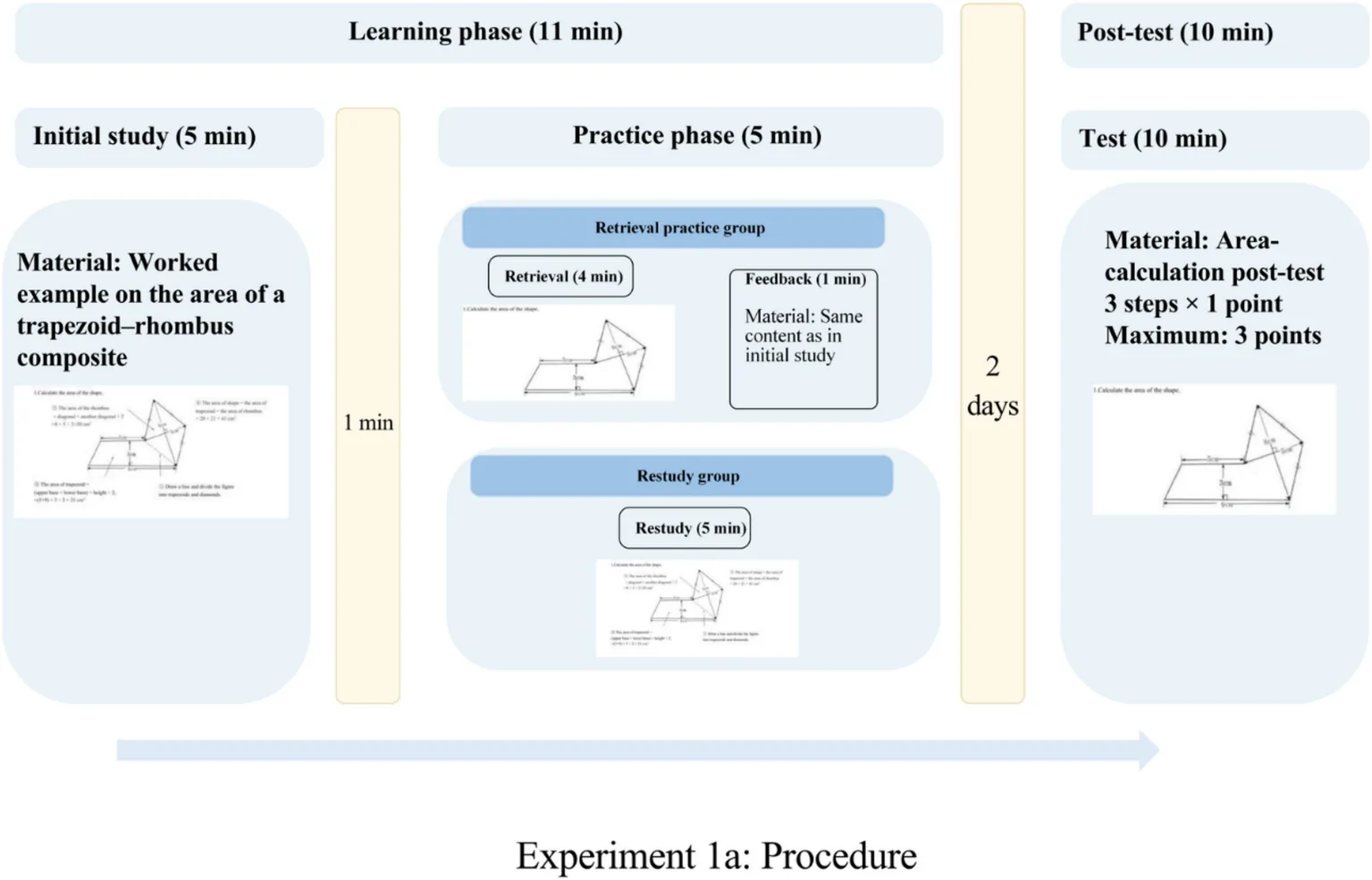
Experiment 1a procedure.

#### 2.1.2 Results

A two-factor ANOVA with working memory capacity (high, low) and learning method (retrieval practice, restudy) as independent variables, and final test score as the dependent variable was conducted. The results demonstrated significant main effects of learning method, *F*(1, 86) = 9.253, *p* < 0.05, $\eta_{p}^{2}$ = 0.097, and working memory capacity, *F*(1, 86) = 17.420, *p* < 0.001, $\eta_{p}^{2}$ = 0.168), for low-complexity materials. Additionally, the interaction effect between working memory capacity and learning method was significant, *F*(1,86) = 22.758, *p* < 0.001, $\eta_{p}^{2}$ = 0.209.

Simple effects analysis showed that for learners with low working memory capacities, there was significant difference in the final test scores between the retrieval practice group and the restudy group, with the former displaying better performance than the latter (95% CI [0.705, 1.526], *p* < 0.001, Cohen’s *d* = 7.817). However, there was no significant difference for learners with high working memory capacities, *p* = 0.214. Additionally, no significant difference was observed in the final test scores between learners with high and low working memory capacities within the retrieval practice group, *p* = 0.663. In the restudy group, learners with high working memory capacities performed significantly better than those with low working memory capacities (95% CI [0.863, 1.692], *p* < 0.001, Cohen’s *d* = 8.665). See Table 1 and Figure 2.

**Table 1:** The final test scores for low-complexity materials.

Working memory capacities	Learning method	*n*	Score (*M* ± SD)
Low	Retrieval practice	23	2.565 ± 0.788
	Restudy	20	1.450 ± 0.510
High	Retrieval practice	25	2.480 ± 0.823
	Restudy	22	2.727 ± 0.456

**Figure 2:**
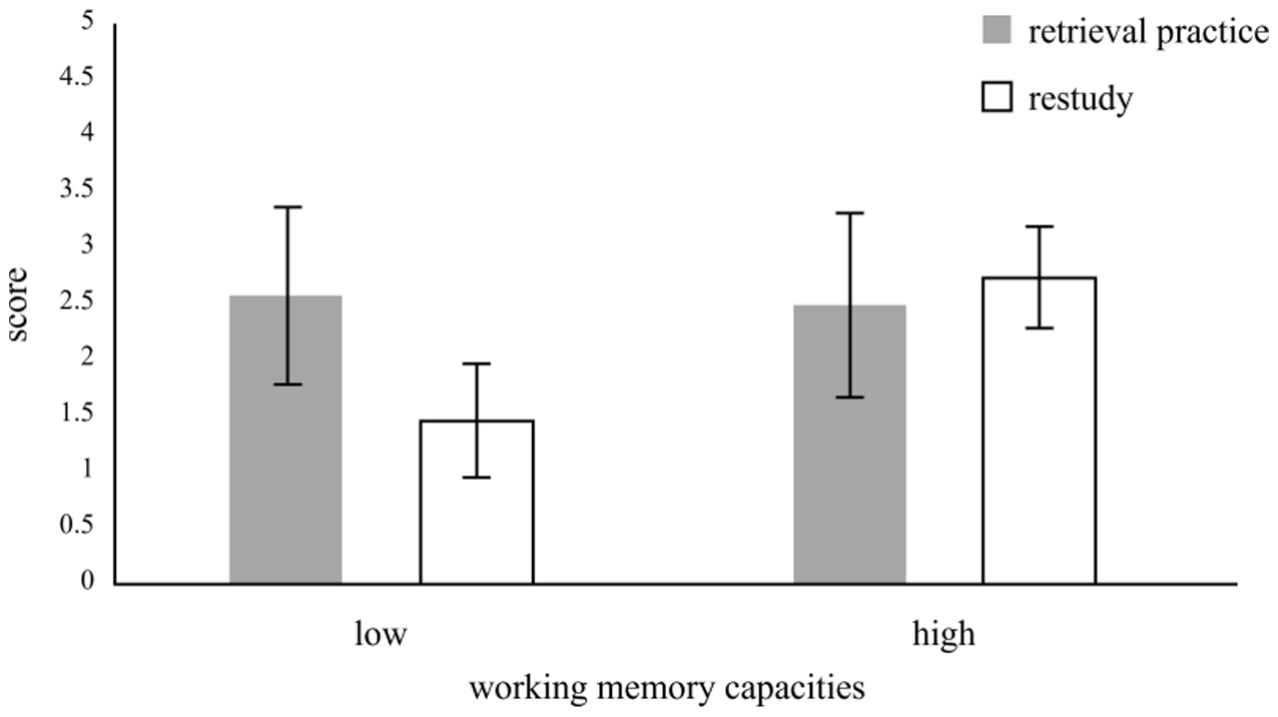
Test scores of the four groups of participants in learning low-complexity materials. Error bars represent standard deviations (SDs).

### 2.2 Experiment 1b: the impact of working memory capacity on the testing effect with high-complexity materials

#### 2.2.1 Method

##### 2.2.1.1 Participants

The selection criteria were the same as in Experiment 1a. A total of 99 students participated, including 52 students with high working memory capacity (16 female, 36 male; average age 10.25 years, SD = 0.44) and 47 students with low working memory capacity (24 female, 23 male; average age 10.26 years, SD = 0.44). WMC scores ranged from 2.5 to 3.0 in the low-working-memory-capacity group and from 4.5 to 6.5 in the high-working-memory-capacity group.

##### 2.2.1.2 Design

The Experimental design was identical to that used in Experiment 1a.

##### 2.2.1.3 Experiment materials

The materials were the same as in Experiment 1a. However, the post-test material differed by including five problem-solving steps, each awarded one point for correct answer. The total score was five points.

##### 2.2.1.4 Procedure

The experimental procedure was identical to Experiment 1a.

#### 2.2.2 Results

A two-factor ANOVA with working memory capacity (high, low) and learning method (retrieval practice, restudy) as independent variables, and final test score as the dependent variable was conducted. For high-complexity materials, significant main effects were found for learning method, *F*(1,95) = 23.867, *p* < 0.001, $\eta_{p}^{2}$ = 0.201, and working memory capacity, *F*(1,95) = 19.707, *p* < 0.001, $\eta_{p}^{2}$ = 0.172. The interaction effect between working memory capacities and learning method was also significant, *F*(1,95) = 44.467, *p* < 0.001, $\eta_{p}^{2}$ = 0.319.

Simple effect analysis revealed that learners with high working memory capacities performed significantly better in the retrieval practice group than in the restudy group (95% CI [1.716, 2.785], *p* < 0.001, Cohen’s *d* = 11.194). In contrast, learners with low working memory capacities showed no significant difference between groups (*p* = 0.2). Additionally, within the retrieval practice group, learners with high working memory capacities exceeded those with low working memory capacities (95% CI [1.611, 2.716], *p* < 0.001, Cohen’s *d* = 10.987). There was no significant difference in the restudy group between learners with high and low working memory capacities. See Table 2 and Figure 3.

**Table 2:** The mean and standard deviation of the final test scores of high-complexity materials.

Working memory capacities	Learning method	*n*	Score (*M* ± SD)
Low	Retrieval practice	25	1.881 ± 1.054
	Restudy	22	2.227 ± 0.922
High	Retrieval practice	23	4.043 ± 0.976
	Restudy	29	1.793 ± 0.902

**Figure 3:**
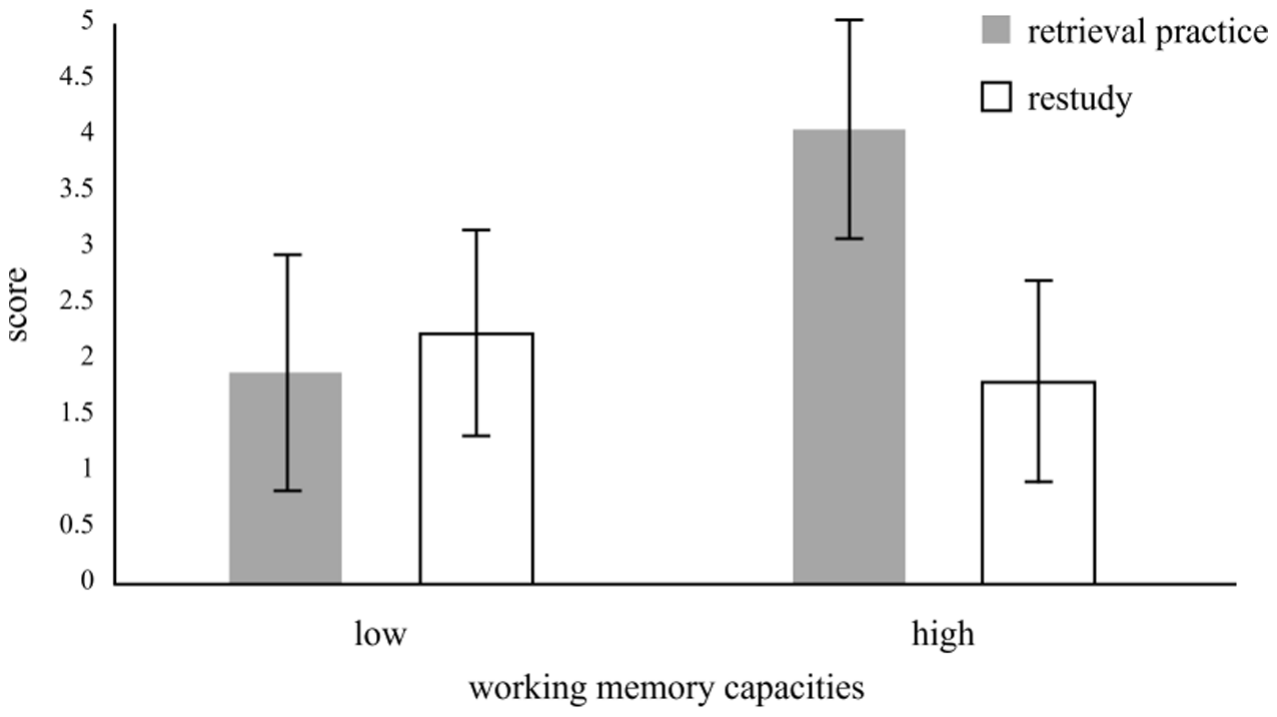
Test scores of the four groups of participants in studying high-complexity materials. Error bars represent standard deviations (SDs).

### 2.3 Discussion

Experiment 1a showed that a testing effect emerged among learners with lower working memory capacity, but not among those with higher working memory capacity, when learning low-complexity materials. In contrast, Experiment 1b showed that, for high-complexity materials, the testing effect emerged among learners with higher working memory capacity but not among those with lower working memory capacity.

The results of Experiment 1a support and extend previous findings by Agarwal et al. (2016). This pattern may reflect differences in available working memory resources during learning. For learners with higher working memory capacity, low-complexity materials, characterized by limited interelement interactivity, may place relatively modest demands on cognitive resources. These learners may therefore process the material effectively through either retrieval practice or restudy. In contrast, despite the apparent simplicity of the materials, learners with lower working memory capacity may still require more effective learning strategies because their cognitive resources are more limited.

Experiment 1a also showed only small differences in final test performance between learners with higher and lower working memory capacity. Notably, learners with lower working memory capacity performed particularly well after retrieval practice, even outperforming their higher-capacity counterparts. This pattern may reflect the lower complexity of the materials, which may have supported successful retrieval for these learners. By contrast, learners with higher working memory capacity appeared to benefit more from restudy, possibly because they could consolidate knowledge more effectively during repeated exposure. Learners with lower working memory capacity may have allocated more cognitive resources during initial learning, leaving fewer resources available for efficient restudy. Future studies should examine these differences more directly, particularly in relation to cognitive load.

The results of Experiment 1b were inconsistent with those of Minear et al. (2018). This discrepancy may partly reflect methodological differences. Participants in Minear et al. (2018) may have used additional learning strategies, such as keyword mnemonics, alongside restudy and retrieval practice. Such strategy use could have masked the influence of working memory capacity on the testing effect. In the present study, by contrast, participants appear to have followed the prescribed strategies, as indicated by post-experiment interviews. A further difference concerns participant characteristics. Minear et al. (2018) examined college students, who may have developed more stable and individualized learning strategies than younger learners and may have integrated these strategies into their study practices.

Nevertheless, Experiment 1b showed no observable testing effect among learners with lower working memory capacity when they learned high-complexity materials. This absence may be explained by two related factors. First, high-complexity materials may have imposed greater cognitive load on these learners, limiting effective encoding and subsequent retrieval. Second, their more limited cognitive resources may have made it difficult to process multiple elements and their interrelations during encoding. This difficulty may have reduced initial retrieval success. According to the bifurcation model proposed by Kornell et al. (2011), initial retrieval success is a key determinant of the testing effect.

In light of these considerations, the subsequent experiments examined alternative forms of retrieval practice designed to support learners with lower working memory capacity when learning complex materials. Experiment 2 investigated repeated retrieval under specified interval schedules, whereas Experiment 3 examined diminishing-cues retrieval practice as a way to support successful retrieval.

## 3 Experiment 2: the effect of repeated retrieval under specified interval schedules for low-working-memory-capacity learners using complex materials

### 3.1 Method

#### 3.1.1 Participants

The sample size was estimated using G*Power 3.1, indicating that at least 22 participants were required per group. A total of 240 fourth-grade students with standardized intelligence scores between 90 and 109 were initially screened. From these students, 66 students (27 girls, 39 boys) with low working memory capacity were identified, with an average age of 10.18 years (SD = 0.61). The WMC scores of the low-working-memory-capacity participants ranged from 2.5 to 3.0. High-working-memory-capacity students did not participate in Experiment 2.

The same ethical considerations as in Experiment 1 were applied.

#### 3.1.2 Design

A single-factor between-subject experimental design was adopted. The independent variable was learning method, consisting of three conditions: equal-interval retrieval practice, extended-interval retrieval practice and restudy. The dependent variable was the final test score.

#### 3.1.3 Experiment material

The pre-test and learning material was identical to those used in Experiment 1. The final test material included problems of comparable quality to the learning material, including five steps. Participants received one point for each correct step, resulting in a maximum score of five points.

#### 3.1.4 Procedure

For this experiment, all students in the screening sample completed the pre-test, including the Corsi block-tapping test, before the learning phase began. The Corsi scores were then ranked within the screening sample, and the low-working-memory-capacity participants were selected from the bottom 27%. The interval between the pre-test and the learning phase was 2 days.

The pre-test was identical to Experiment 1.

During the learning phase, all three groups studied the examples for 8 min.

The equal-interval retrieval practice group engaged in retrieval practice for 5 min following a 3-min interval. This was followed by 1 min feedback. This retrieval-feedback cycle was repeated twice.

The extended-interval retrieval practice group retrieved the learned content initially after a 1-min interval, followed by retrieval practice at progressively longer intervals (3 min, then 5 min). Each retrieval session lasted 5 min and was followed by 1 min of feedback.

The restudy group restudied the materials for 6 min after a 3-min interval, and then repeated the process twice.

All participants completed the final test 2 days after the learning phase.

### 3.2 Results

A one-way ANOVA was conducted with learning method as the independent variable and the final test score as the dependent variable. Results showed a significant main effect of learning method, *F*(2,63) = 10.246, *p* < 0.001, $\;\eta_{p}^{2}$ = 0.245. Post-hoc comparisons showed that both the extended-interval retrieval practice group and the equal-interval retrieval practice group performed significantly better than the restudy group (95% CI [0.54, 1.92], *p* < 0.01, Cohen’s *d* = 1.104; 95% CI [0.76, 2.15], *p* < 0.001, Cohen’s *d* = 1.379) (see Table 3). However, there was no significant difference in the final test scores between the extended-interval and equal-interval retrieval practice groups, *p* = 0.513. The differences in test performance are shown in Figure 4.

**Table 3:** Mean and standard deviation of the final test scores for high-complexity materials.

Learning method	*n*	Score (*M* ± SD)
Equal-interval	22	3.500 ± 1.225
Extended-interval	22	3.272 ± 1.316
Restudy	22	2.045 ± 0.844

**Figure 4:**
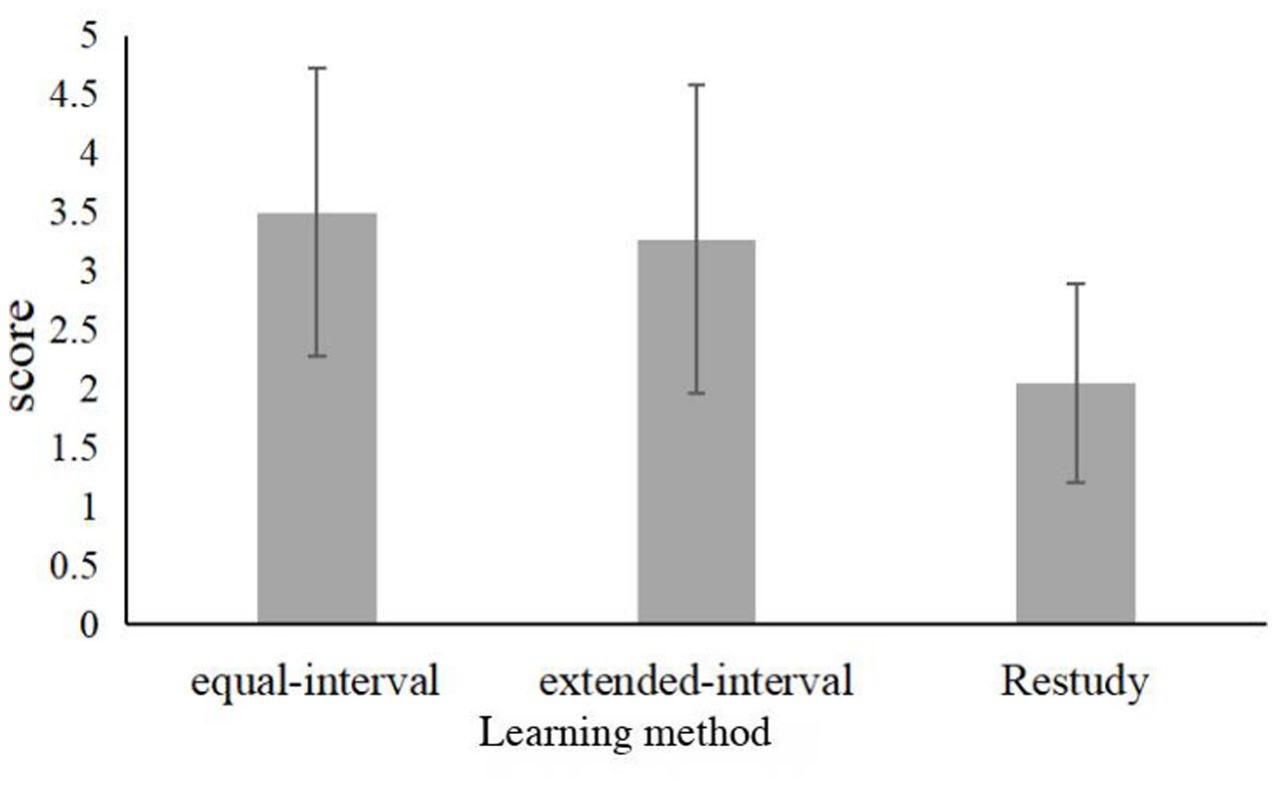
Comparison of final test scores among three groups in learning high-complexity materials. Error bars represent standard deviations (SDs).

### 3.3 Discussion

The findings of Experiment 2 indicate that, under the repeated-retrieval schedules tested here, retrieval practice enhanced delayed-test performance among learners with lower working memory capacity who studied complex materials. Across the equally spaced and expanding schedules, learners in the retrieval-practice conditions achieved higher final-test scores than those in the restudy condition. Because the experiment did not include a massed-retrieval control condition, these results do not isolate an independent benefit of spacing from the effects of repeated retrieval and its accompanying feedback.

Consistent with the prediction outlined in the Introduction, these results are compatible with the resource-based account proposed by Leahy and Sweller (2019). However, the present study did not directly measure resource depletion or recovery. Intervals between retrieval-practice activities may have allowed learners with lower working memory capacity to use repeated retrieval more effectively, but this interpretation remains tentative.

In this experimental context, both repeated-retrieval schedules produced higher final-test performance than restudy. The absence of a significant difference between the two schedules does not establish that one interval configuration was superior, nor does it demonstrate that spacing itself caused the observed benefit or that working-memory resources recovered during the intervals.

## 4 Experiment 3: the influence of diminishing-cues retrieval practice on learning high-complexity materials among learners with low working memory capacities

### 4.1 Method

#### 4.1.1 Participants

Participants consisted of 66 students with low working memory capacity, including 31 girls and 35 boys, with an average age of 10.21 years (SD = 0.41). Only students with low working memory capacity participated in this experiment. Their WMC scores ranged from 2.5 to 3.0.

#### 4.1.2 Design

A single-factor between-subjects design was adopted. The independent variable was the learning method, consisting of three conditions: diminishing-cues retrieval practice, cue-free retrieval practice, and restudy. The dependent variable was the final test score.

#### 4.1.3 Experiment material

The experimental materials were identical to those used in Experiment 2.

#### 4.1.4 Procedure

For this experiment, all students in the screening sample completed the pre-test, including the Corsi block-tapping test, before the learning phase began. The Corsi scores were then ranked within the screening sample, and the low-working-memory-capacity participants were selected from the bottom 27%. The interval between the pre-test and the learning phase was 2 days.

The pre-test phase was identical to that described in Experiment 1.

Learning phase: Initially, participants in all three groups studied the samples for 8 min.

Diminishing-cues retrieval practice group: After a 3-min interval, participants engaged in retrieval practice using diminishing cues for 6 min. This retrieval process was repeated twice, with the cues gradually reduced each time. For example, during the first retrieval session, participants received cues detailing calculation methods for the height and area of the parallelogram, trapezoid, and shadow parts. In the second retrieval, cues were provided only for the calculation methods of the trapezoid and shaded areas. No cues were presented during the third retrieval session.

Cue-free retrieval group: Participants retrieved the learned content without cues for 5 min after a 3-min interval, followed by feedback lasting 1 min. This retrieval-feedback cycle was repeated twice.

Restudy group: Participants restudied the material for 6 min, after a 3-min interval, with this restudy session repeated twice.

All participants completed the final test 2 days after the learning phase.

### 4.2 Results

A one-way ANOVA was conducted with learning method as the independent variable and the final test score as the dependent variable. Results showed a significant main effect of learning method, *F*(2,63) = 34.464, *p* < 0.001, η_p_^2^ = 0.522. Post-hoc analysis revealed that the final test scores of the diminishing-cues retrieval practice group were significantly higher than those of the cue-free retrieval group, 95% CI [0.39, 1.52], *p* < 0.05, Cohen’s *d* = 0.970. Additionally, the diminishing-cues retrieval practice group outperformed the restudy group, 95% CI [1.76, 2.88], *p* < 0.001, Cohen’s *d* = 2.300. Furthermore, the cue-free retrieval practice group achieved significantly higher final test scores than the restudy group, 95% CI [0.80, 1.92], *p* < 0.001, Cohen’s *d* = 1.335. These results are summarized in Table 4 and visualized in Figure 5.

**Table 4:** Mean and standard deviation of final test scores for high-complexity materials.

Learning method	*n*	Score (*M* ± SD)
Diminishing cue	22	4.409 ± 0.734
Cue-free	22	3.454 ± 1.184
Restudy	22	2.090 ± 0.811

**Figure 5:**
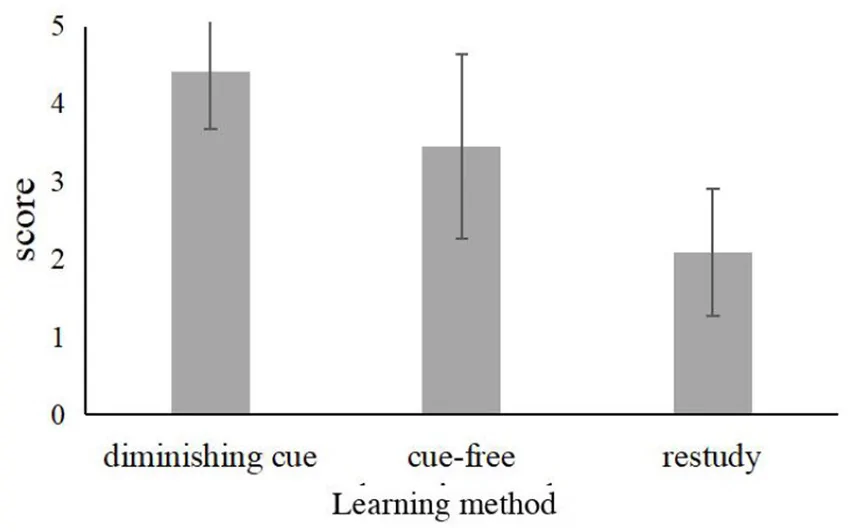
Comparison of post-test scores among the three groups. Error bars represent standard deviations (SDs).

### 4.3 Discussion

The results of Experiment 3 showed that diminishing-cues retrieval practice produced better learning outcomes than cue-free retrieval practice for learners with lower working memory capacity. Moreover, both retrieval-practice conditions outperformed the restudy condition, indicating a robust testing effect.

Because complex materials involve high element interactivity, learners must manage multiple variables simultaneously in working memory to achieve successful learning outcomes, which increases cognitive burden (Sweller and Chandler, 1994). This challenge may be particularly pronounced for learners with lower working memory capacity, whose initial retrieval success is often lower. As a result, these learners may find it difficult to engage fully with retrieval practice strategies.

Diminishing-cues retrieval practice may have served as a scaffold that reduced cognitive load while supporting successful initial retrieval. As the cues were gradually withdrawn, learners with lower working memory capacity may have internalized the learning material more effectively and become better able to solve problems independently. Because first-attempt responses were not retained, this proposed mechanism cannot be directly evaluated in the present study.

Relationship between Experiments 2 and 3. The diminishing-cues and cue-free retrieval conditions in Experiment 3 followed the basic timing structure of the equally spaced retrieval condition in Experiment 2. Experiment 3 therefore extended the repeated-retrieval procedure by examining whether progressively reduced cues could further support low-working-memory-capacity learners studying high-complexity materials. Descriptively, mean final-test performance increased from 3.500 in the equally spaced retrieval condition of Experiment 2 to 4.409 in the diminishing-cues condition of Experiment 3, a difference of 0.909 points, or approximately 26.0%. The cue-free condition in Experiment 3 had a mean of 3.454, close to the Experiment 2 equally spaced condition. This pattern is consistent with the possibility that adding diminishing cues to equally spaced repeated retrieval may provide additional learning support.

These cross-experiment comparisons are descriptive because the samples and implementation differed. The more direct evidence for the benefit of diminishing-cues retrieval comes from the within-experiment comparisons in Experiment 3, where the diminishing-cues condition produced significantly higher final-test scores than both cue-free retrieval and restudy. Diminishing cues may scaffold early retrieval while progressively increasing the demand for independent retrieval across practice cycles. However, because initial retrieval data were not retained, the present study cannot determine whether increased initial retrieval success explains the final-test advantage.

## 5 General discussion

Across four experiments, this study examined how working memory capacity shapes the testing effect when learners study materials that vary in complexity. The findings also showed that repeated retrieval under the schedules tested in Experiment 2 and diminishing-cues retrieval practice in Experiment 3 facilitated learning of high-complexity materials among learners with lower working memory capacity.

### 5.1 The influence of material complexity on the effect of working memory capacities on the testing effect

The findings of Experiment 1 show that the benefits of retrieval practice depend on both learners’ working memory capacity and the complexity of the learning materials. For low-complexity materials, learners with lower working memory capacity showed greater testing benefits than those with higher working memory capacity. In contrast, for high-complexity materials, learners with higher working memory capacity benefited more from retrieval practice than their lower-capacity counterparts. These results are consistent with Agarwal et al. (2016) and support the hypothesis that material complexity moderates the relation between working memory capacity and the testing effect.

The contrasting patterns in Experiments 1a and 1b can also be considered in light of this resource-based account. In Experiment 1b, the absence of a significant retrieval benefit among lower-WMC learners is compatible with the possibility that high-complexity materials placed demands on working memory that left insufficient resources for post-retrieval re-encoding. In Experiment 1a, the benefit observed among lower-WMC learners is consistent with the possibility that lower-complexity materials imposed fewer such demands. However, this interpretation does not directly explain the absence of a significant retrieval benefit among higher-WMC learners in Experiment 1a, and a nonsignificant effect should not be interpreted as evidence that retrieval practice was ineffective for that group. More broadly, Lima and Buratto (2024) noted that individual-difference findings in retrieval-practice research are difficult to compare because of methodological heterogeneity. The present experiments used different samples, material-complexity levels, and score ranges, so their contrasting patterns should not be attributed exclusively to material complexity.

In Experiment 1a, learners with higher and lower working memory capacity studied low-complexity materials involving three core elements: the formula for calculating the area of a rhombus, the formula for calculating the area of a trapezoid, and the distinction between auxiliary lines for the two figures. Regardless of learning strategy, learners with higher working memory capacity appeared able to memorize the target knowledge and understand the steps and principles illustrated in the examples, leading to relatively strong learning outcomes. For learners with lower working memory capacity, however, even these low-complexity materials may have imposed a substantial cognitive burden. Under these conditions, restudy alone may have been insufficient to support effective assimilation of the learning materials. Retrieval practice, by contrast, may have facilitated more effective knowledge acquisition, leading to the observed testing effect.

In Experiment 1b, the high-complexity materials required learners to master additional elements, including area calculations for trapezoids, triangles, and parallelograms, as well as determining the height of parallelograms and sketching auxiliary lines to distinguish shapes. The greater number of interacting elements increased cognitive demands, which may have disproportionately affected learners with lower working memory capacity.

### 5.2 Promoting the testing effect for learning complex materials in learners with low working memory capacities

Building on the findings of Experiment 1, the subsequent experiments examined interventions designed to support learners with lower working memory capacity in learning complex materials. Two factors may explain the difficulties these learners faced. First, the high cognitive demands of complex materials may have consumed a substantial portion of their already limited working memory resources. Second, learners with lower working memory capacity may have had difficulty processing multiple elements simultaneously, which may have reduced their initial retrieval success.

To address these challenges, we tested two practice procedures: repeated retrieval under specified schedules and diminishing-cues retrieval practice. The findings support benefits of these tested procedures for final-test performance, but they do not directly establish working-memory resource recovery or increased initial retrieval success.

Repeated retrieval with intervals facilitated the use of retrieval practice among learners with lower working memory capacity. Intervals may have provided opportunities for recovery, but because no massed-retrieval control or direct resource measure was included, this remains a possible explanation rather than a mechanism established by the present study (Karpicke and Bauernschmidt, 2011).

In this study, we compared two repeated-retrieval schedules: equally spaced retrieval and expanding retrieval. Both schedules produced higher final-test performance than restudy, but the absence of a massed-retrieval condition means that the independent contribution of spacing cannot be determined.

Feedback provided after the two retrieval intervals may have further supported the role of retrieval practice in promoting comprehension of the learning materials. This feedback may have served as a reinforcement mechanism, helping learners perform subsequent cognitive tasks more effectively. Prior research similarly highlights the importance of feedback in supporting deeper comprehension and providing learners with the information needed to complete complex tasks (Ossenberg et al., 2019).

The findings of Experiment 3 indicate that diminishing-cues retrieval practice strengthened the testing effect among learners with lower working memory capacity, outperforming both cue-free retrieval practice and restudy. Together, the findings suggest that repeated retrieval under the schedules tested in Experiment 2 and diminishing-cues retrieval practice can support learners with lower working memory capacity in mastering complex learning materials.

A limitation concerns the interpretation of Experiment 2. Because the design did not include a massed-retrieval control condition, the contribution of spacing cannot be separated from the effects of repeated retrieval and feedback. The equally spaced and expanding schedules also did not differ significantly, so the findings do not establish that one spacing configuration was superior. In addition, the study did not directly measure working-memory resource depletion or recovery. Future research should compare matched massed and spaced retrieval schedules and include direct measures of resource-related processes (Karpicke and Bauernschmidt, 2011).

A further limitation is that the first-attempt retrieval responses were not retained. We therefore could not calculate initial retrieval-success rates across the experiments or determine whether the diminishing-cues procedure improved first-attempt performance. Informal observations during implementation suggested that cues may have helped some participants retrieve the material, but these observations were not systematically recorded or analyzed and should not be treated as statistical evidence. Future research should retain responses before feedback and across subsequent retrieval attempts to test whether changes in initial retrieval success are associated with later learning outcomes.

## 6 Conclusion

1. When learning low-complexity materials, learners with low working memory capacities benefited more from retrieval practice than from restudy. However, retrieval practice and restudy yielded similar outcome for learners with high working memory capacities.
2. When learning high-complexity materials, learners with high working memory capacities performed better with retrieval practice than with restudy. However, for learners with low working memory capacities, restudy and retrieval practices led to comparable learning outcomes.
3. When learning high-complexity materials, repeated retrieval under the schedules tested in Experiment 2 and diminishing-cues retrieval practice successfully promoted the testing effect among learners with low working memory capacity. The independent contribution of spacing cannot be determined from the present design.

## Data Availability

The original contributions presented in the study are included in the article/Supplementary material, further inquiries can be directed to the corresponding author/s.
