# Supplementary material for: The influence of working memory capacity and material complexity on the testing effect
Source: Front Psychol. 2026 Oct 30;17:1952951. doi: 10.3389/fpsyg.2026.1952951 (PMC13759727; doi:10.3389/fpsyg.2026.1952951)

CLS

Name:
Gender: boy girl
Age:

In the following options, 1= completely inconsistent, 2 = relatively inconsistent, 3 = somewhat inconsistent, 4 = neutral, 5 = somewhat consistent, 6 = relatively consistent, and 7 = completely consistent.

1. The learning content of this study just now is very complex.
   1 2 3 4 5 6 7

2. The problems involved in the study just now are very complex.
   1 2 3 4 5 6 7

3. The very complex concepts were mentioned in the study just now.
   1 2 3 4 5 6 7

4. The complex content and topics made me put a lot of energy into my study and doing problems.
   1 2 3 4 5 6 7

5-8 Investigate whether the presentation of instructions and materials is clear.

5. The explanation and guidance in the study are very unclear.
   1 2 3 4 5 6 7

6. The explanation and instruction used in the study is full of vague and difficult words to understand.
   1 2 3 4 5 6 7

7. In terms of the learning effect, the explanation and guidance in the study are very poor.
   1 2 3 4 5 6 7

8. In the study just now, the unclear explanation and guidance effect is very poor.
   1 2 3 4 5 6 7

Pre-test

Pre-test：

The area of the rectangle:______________________________

The area of the square: _______________________________

The area of the parallelogram:_________________________

The area of a rhombus: _______________________

The area of the trapezoid: _____________________________

The area of the triangle:________________________________

Learning

Simple materials (high element interactivity) :

1.Calculate the area of the shape.

③ The area of the rhombus
= diagonal × another diagonal ÷ 2
$=8 \times 5 \div 2=20\ cm^2$

④ The area of shape = the area of trapezoid + the area of rhombus
$= 20 + 21 = 41\ cm^2$

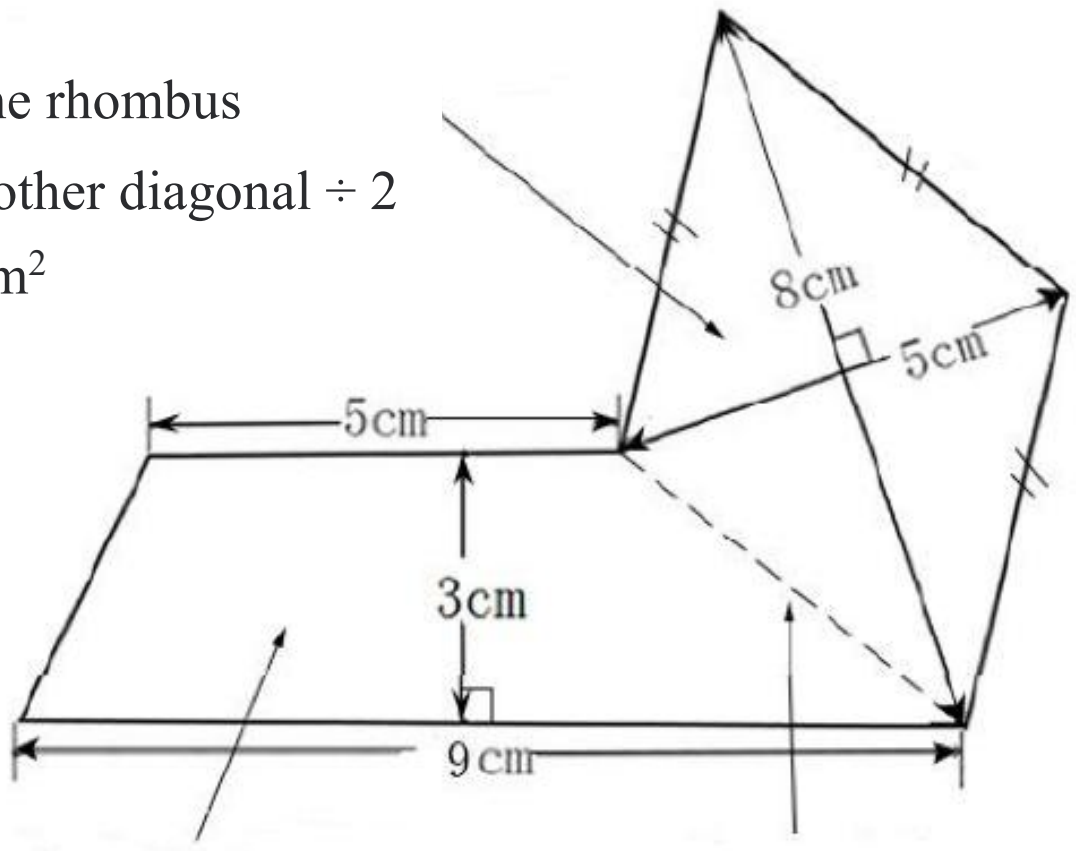

② The area of trapezoid =
(upper base + lower base) × height ÷ 2,
$=(5+9) \times 3 \div 2 = 21\ cm^2$

① Draw a line and divide the figure into trapezoids and diamonds.

Complex materials (low element interactivity) :

1.Calculate the area of the shaded part.

③ Area of the parallelogram =
base × height = $9 \times 6 = 54\ cm^2$

④ The area of a triangle = base × height ÷2
$= 6 \times 5 \div 2 = 15\ cm^2$

② The height of the parallelogram
= 3 + 3 = 6 cm

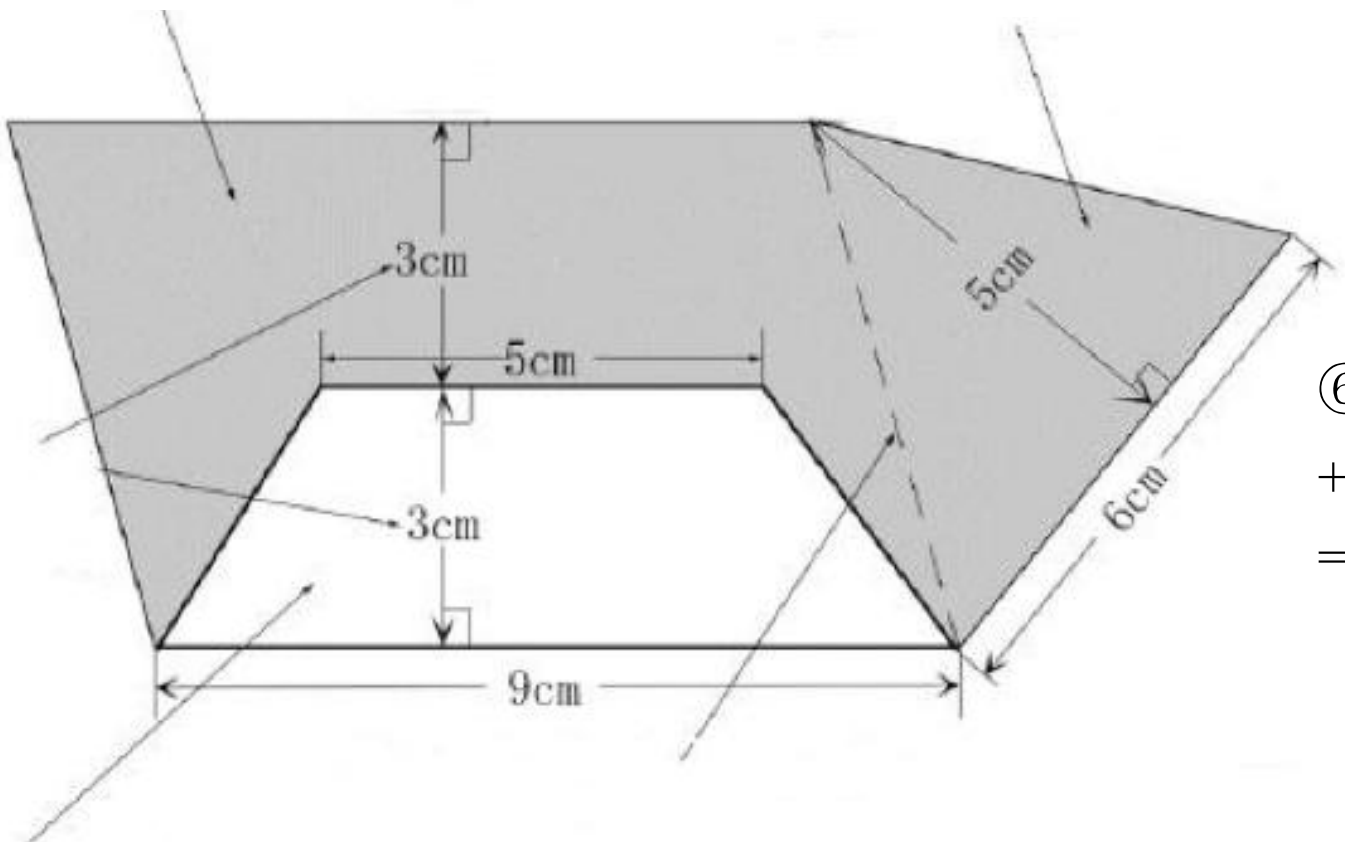

⑥ Shadow area = parallelogram area + triangle area - trapezoidal area
$= 54 + 15 - 21 = 48\ cm^2$

⑤ The area of trapezoid =
(upper base + lower base) × height ÷ 2,
$=(5+9) \times 3 \div 2 = 21\ cm^2$

① Draw a line to divide the figure into parallelograms and triangles.

Post-test material

Low-element interactive material:

1.Calculate the area of the shape.

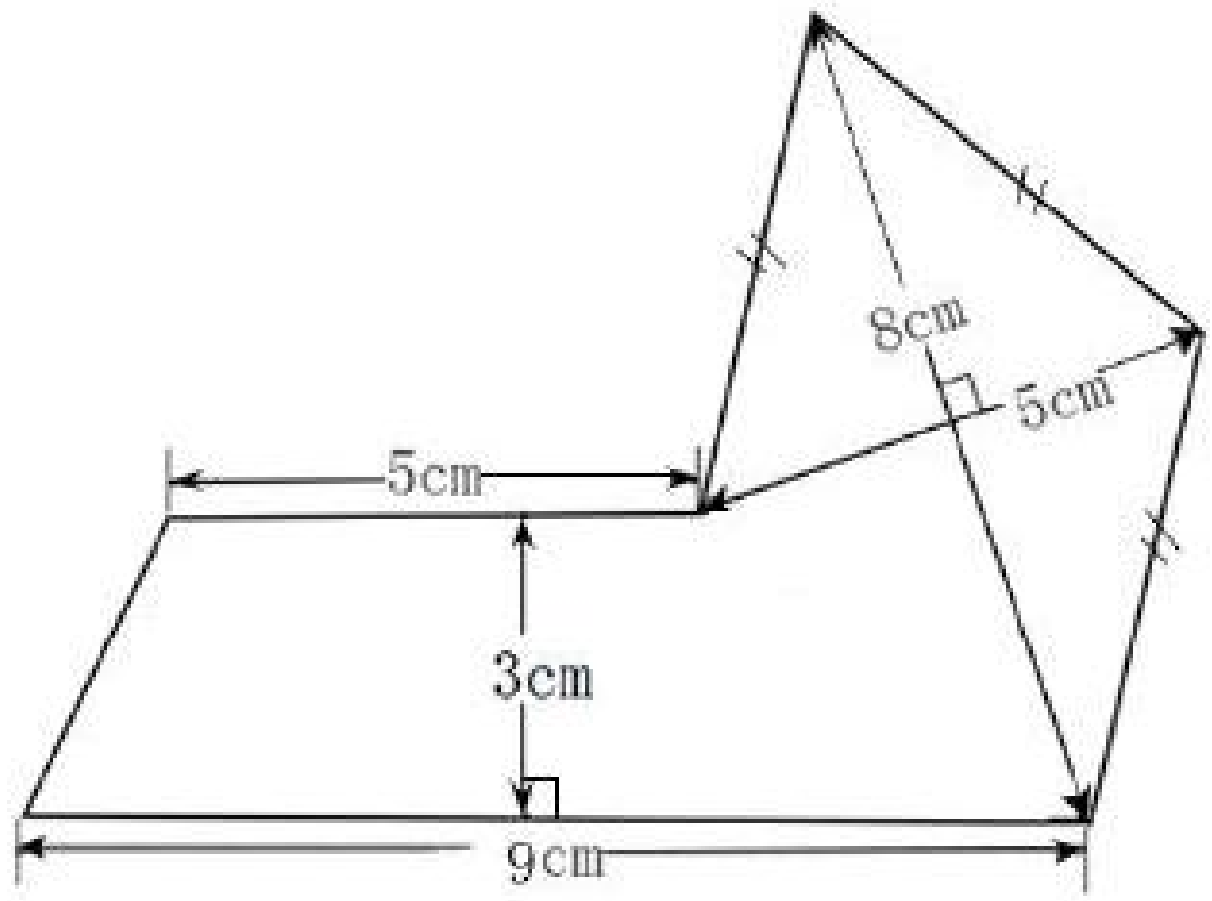

High-element interactive materials :

1.Calculate the area of the shaded part.

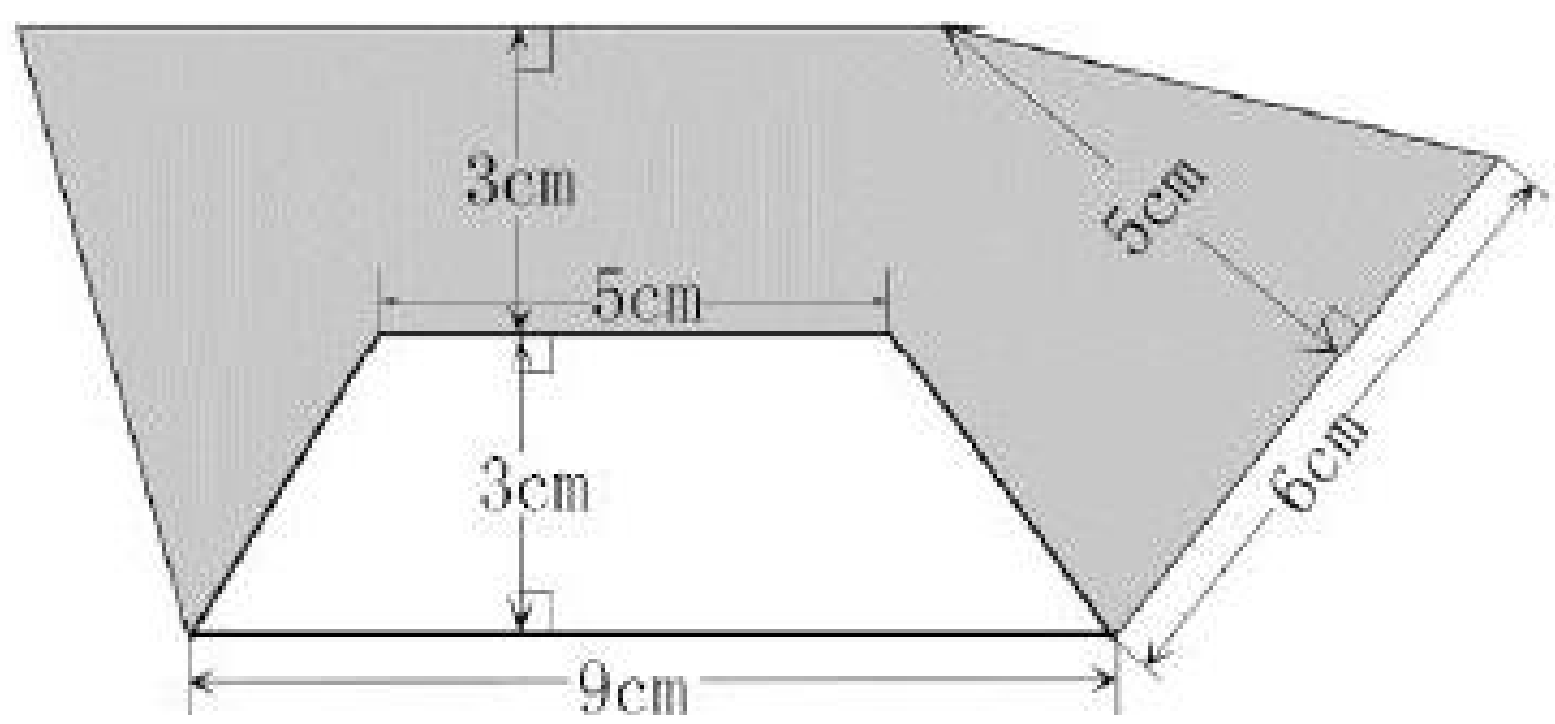

Supplement: Supplementary file 1 [file Data_Sheet_1.PDF]
