# Supplementary material for: The influence of working memory capacity and material complexity on the testing effect
Source: Front Psychol. 2026 Oct 30;17:1952951. doi: 10.3389/fpsyg.2026.1952951 (PMC13759727; doi:10.3389/fpsyg.2026.1952951)
Supplement: Supplementary file 2 [file Data_Sheet_2.PDF]

A

The Combined Raven’s Test (CRT)

Name

Gender　　boy　girl

Age

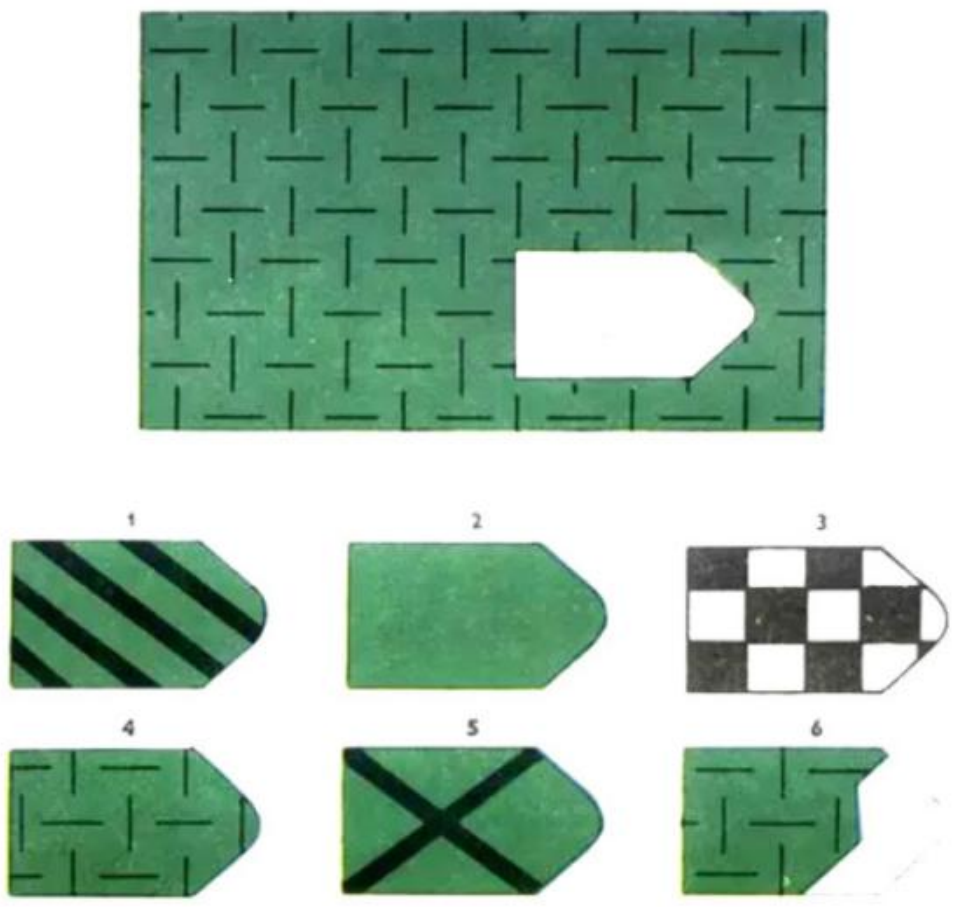

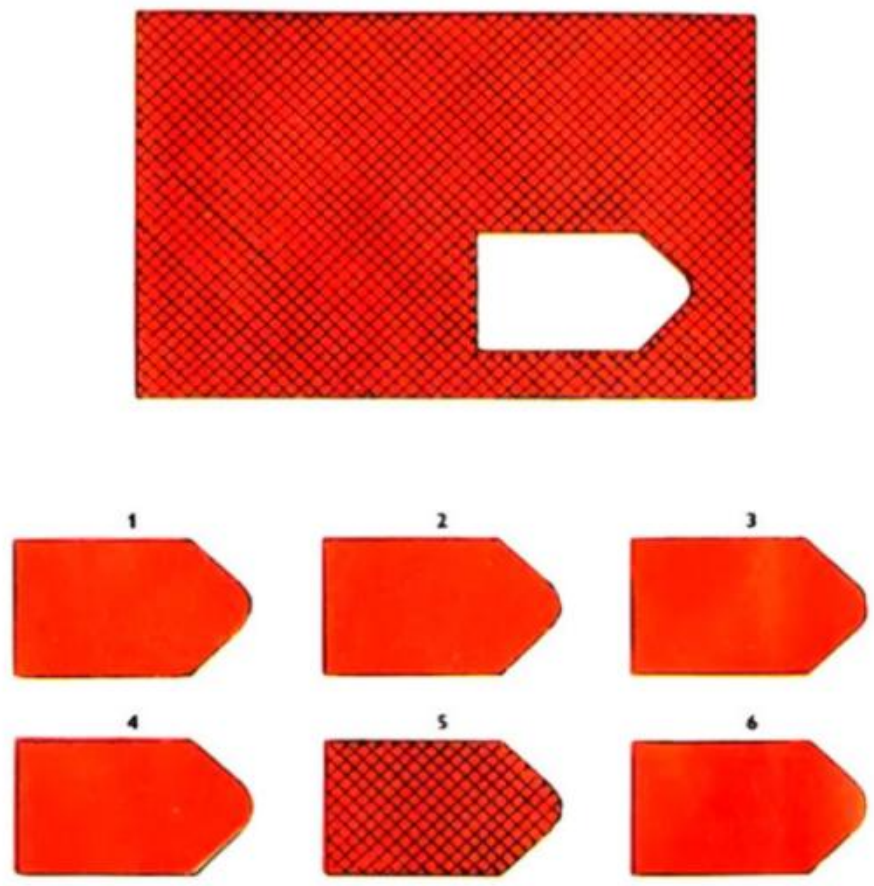

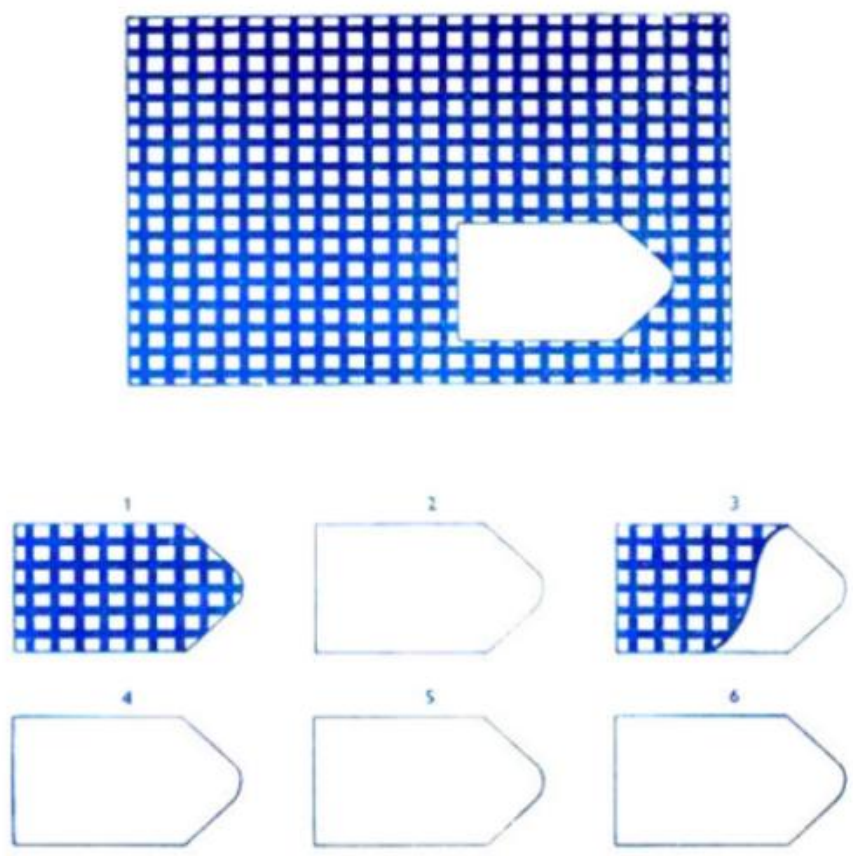

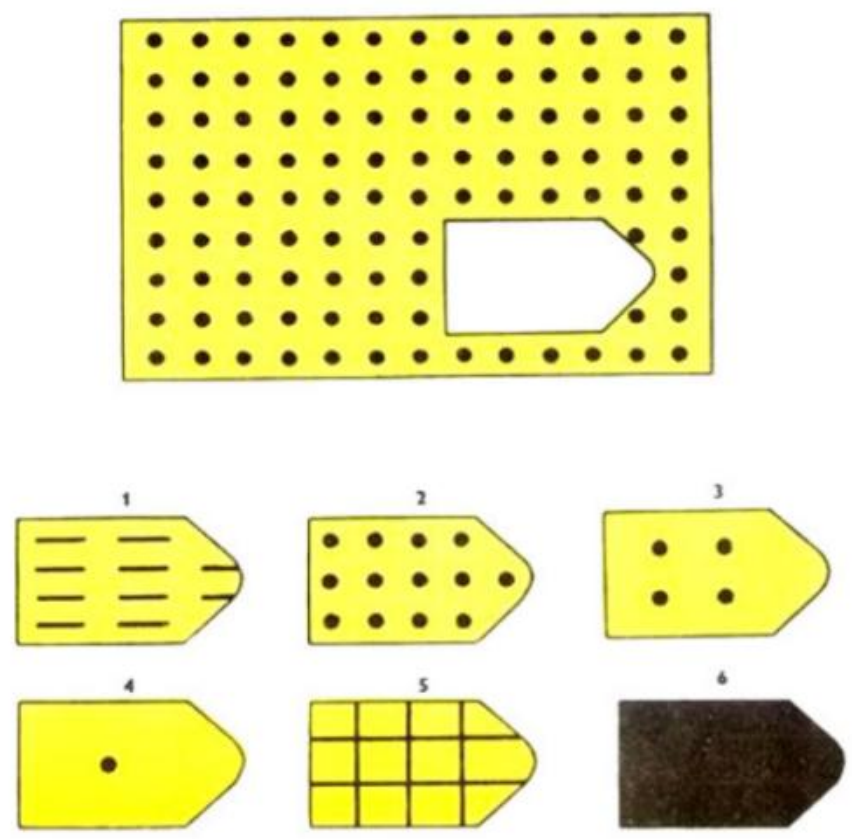

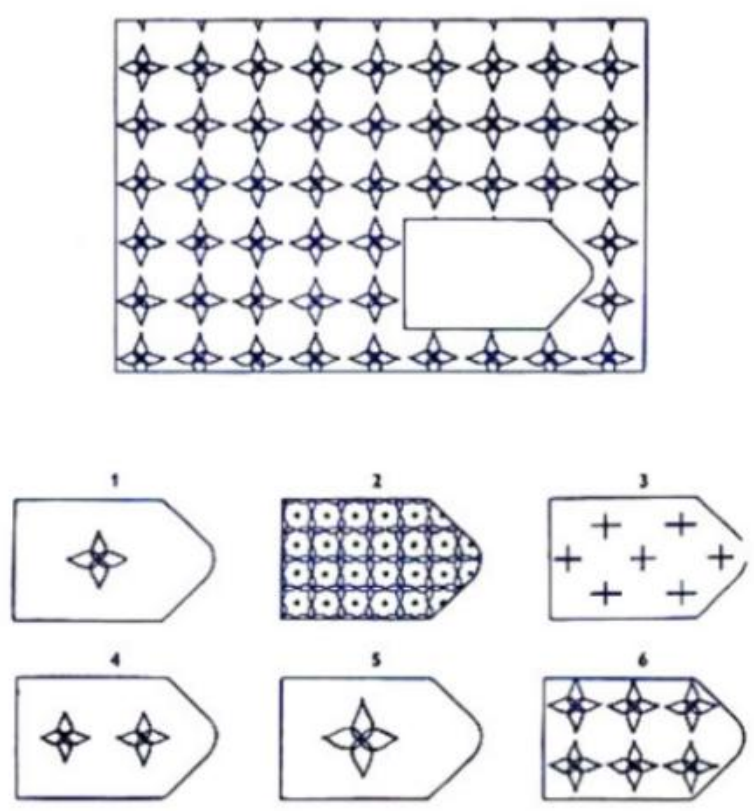

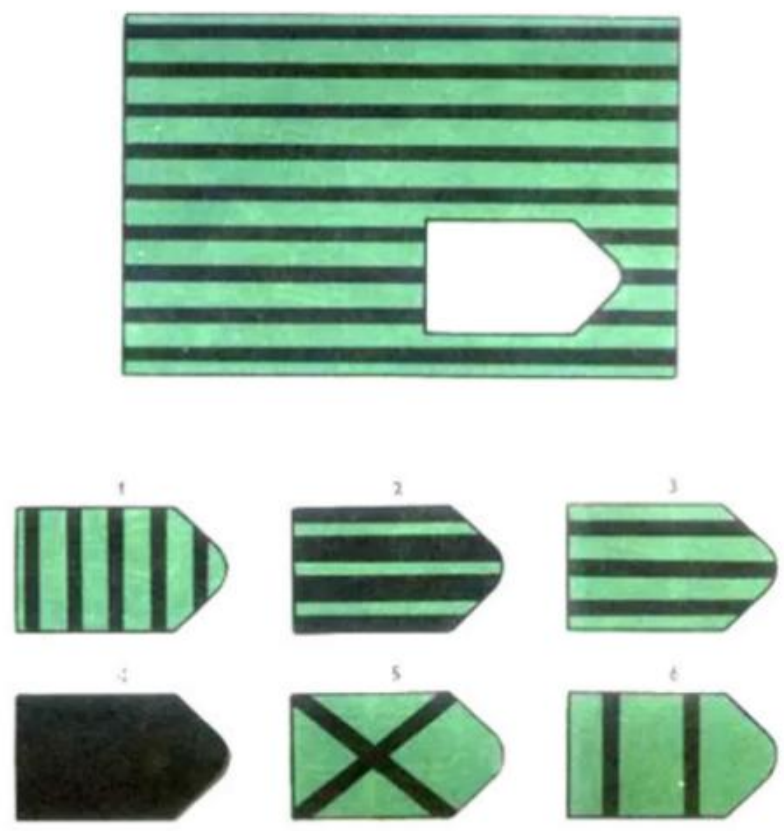

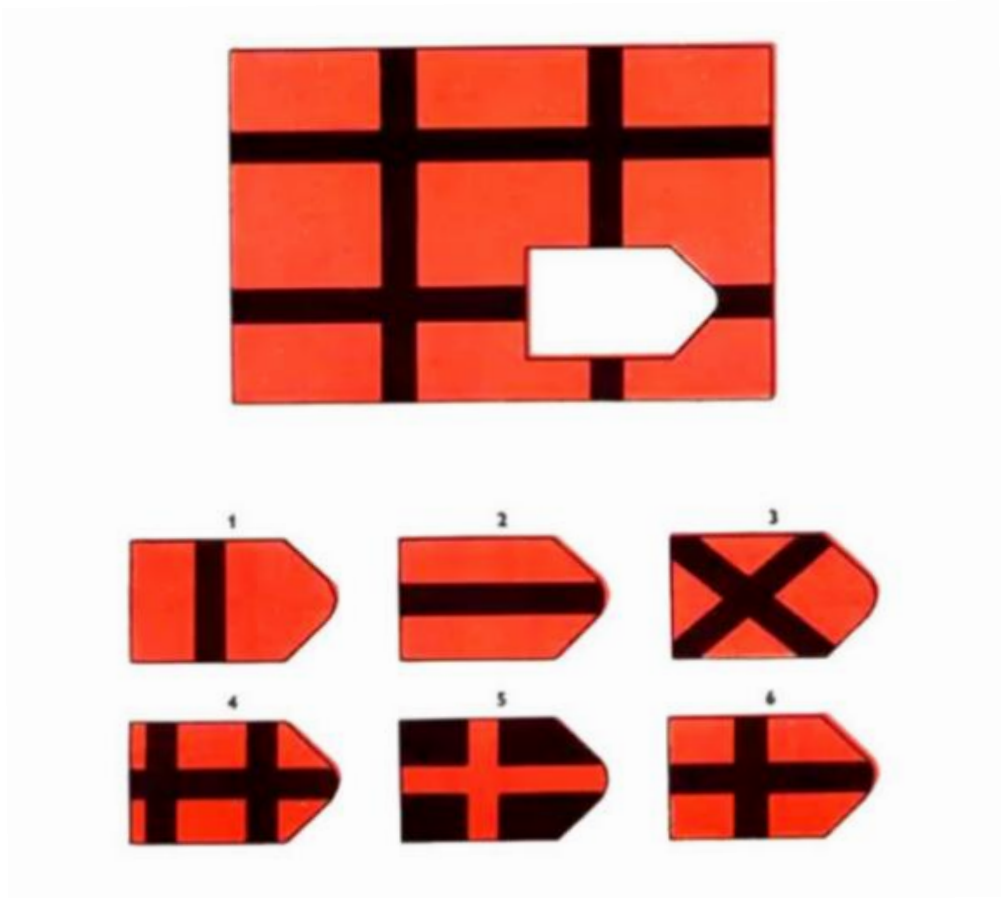

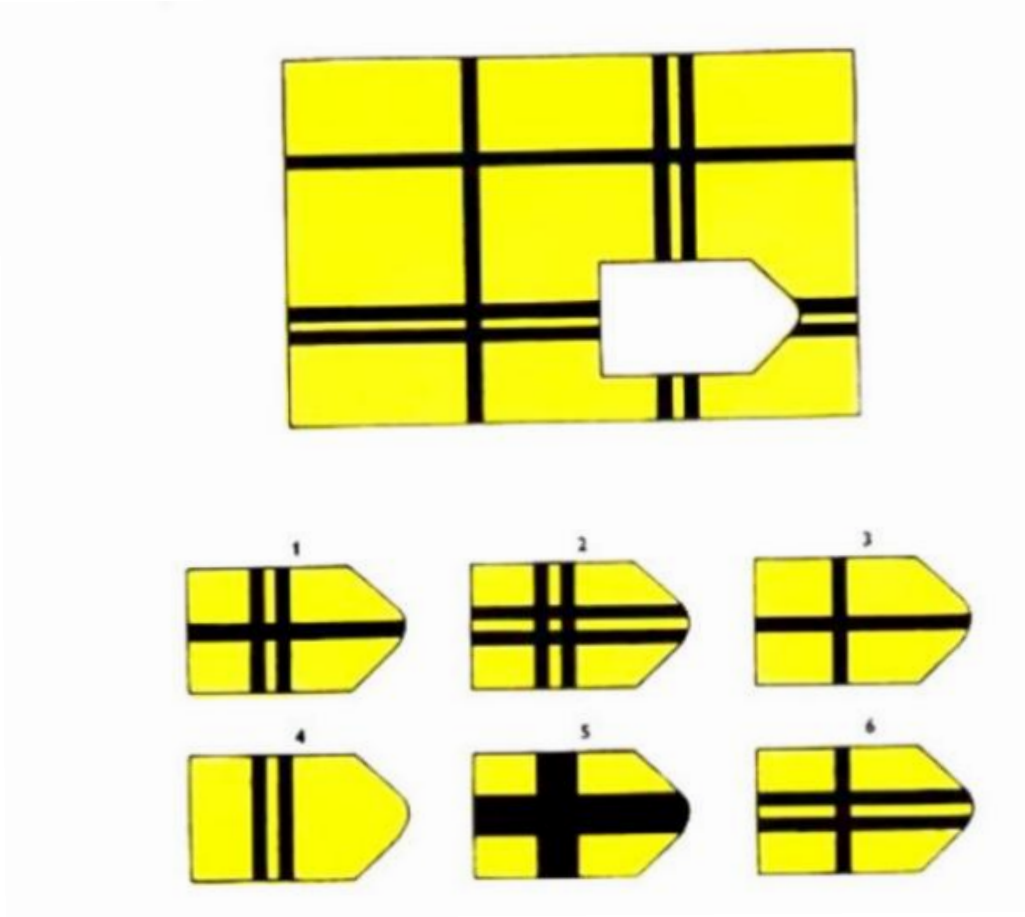

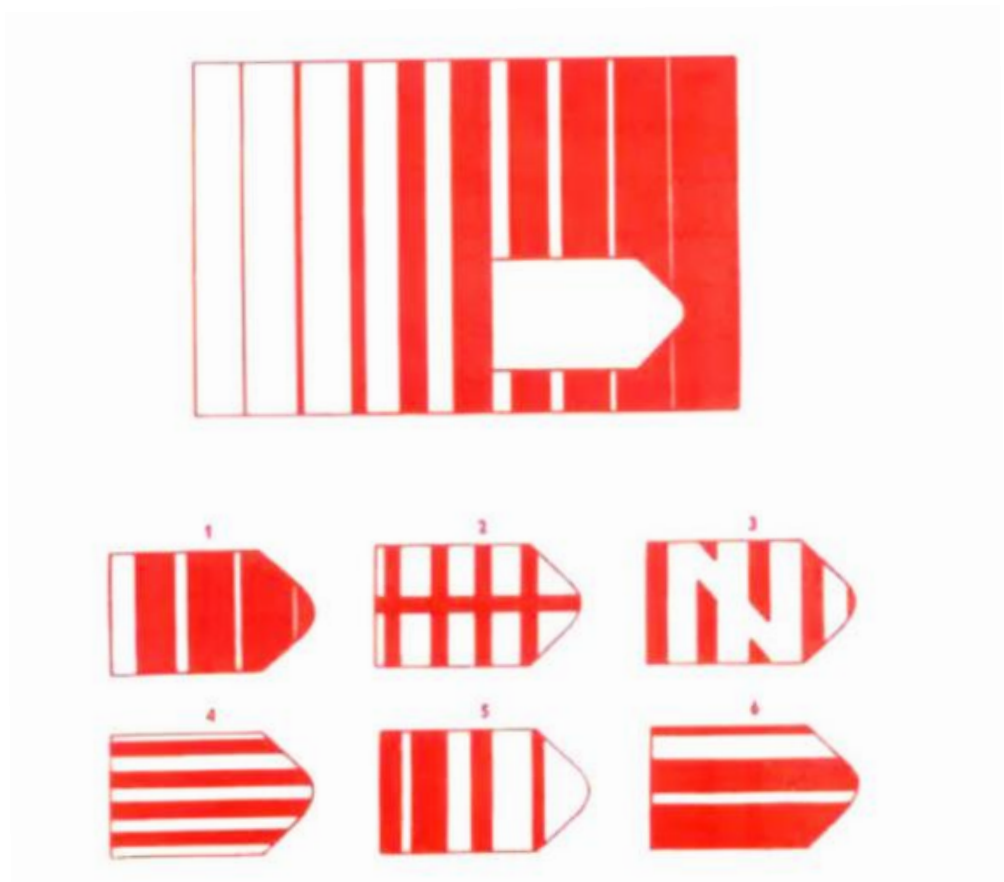

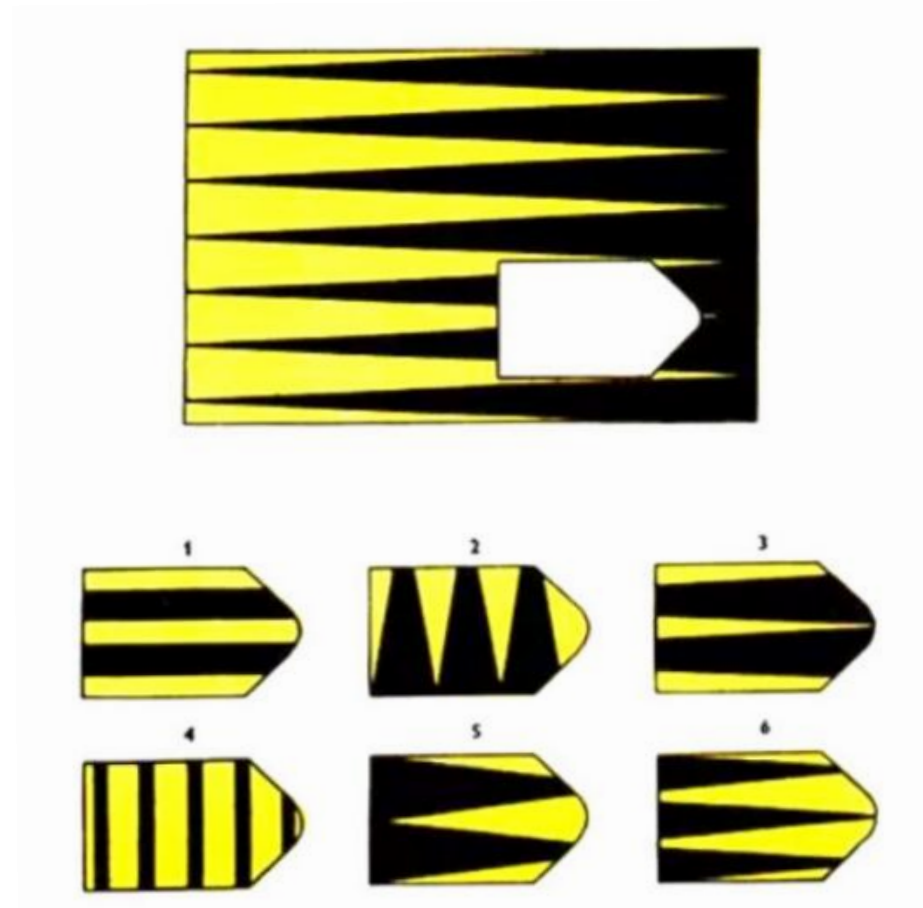

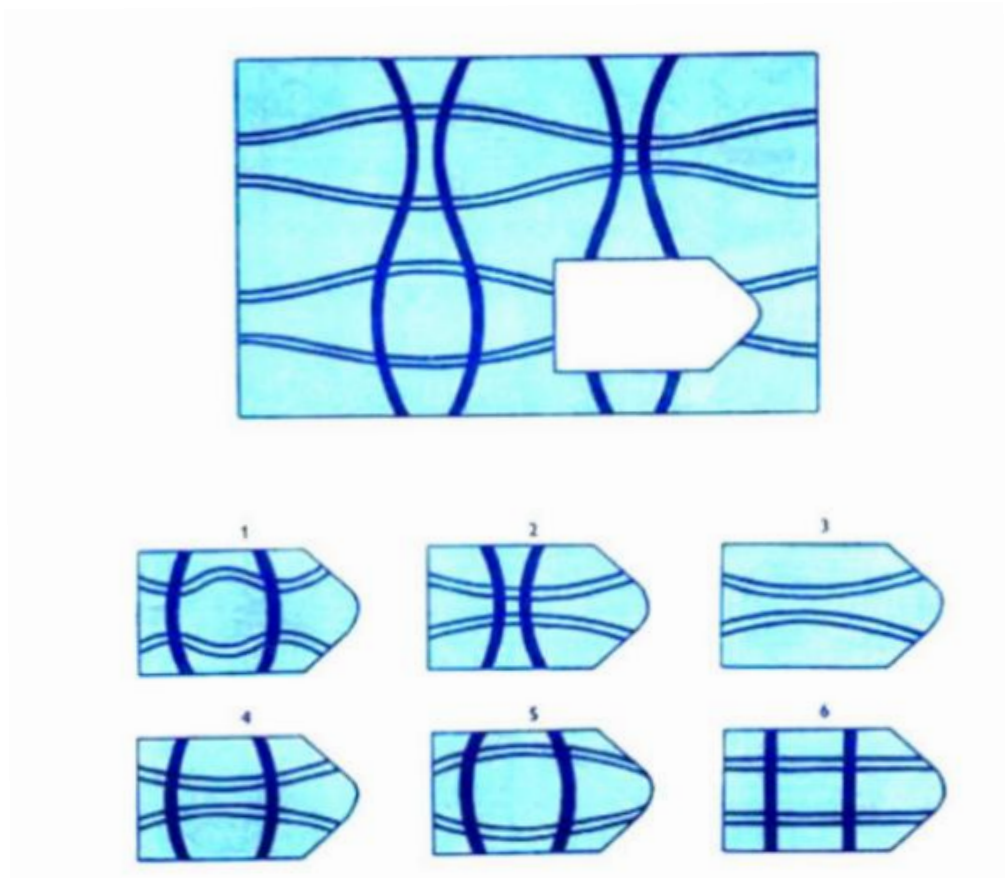

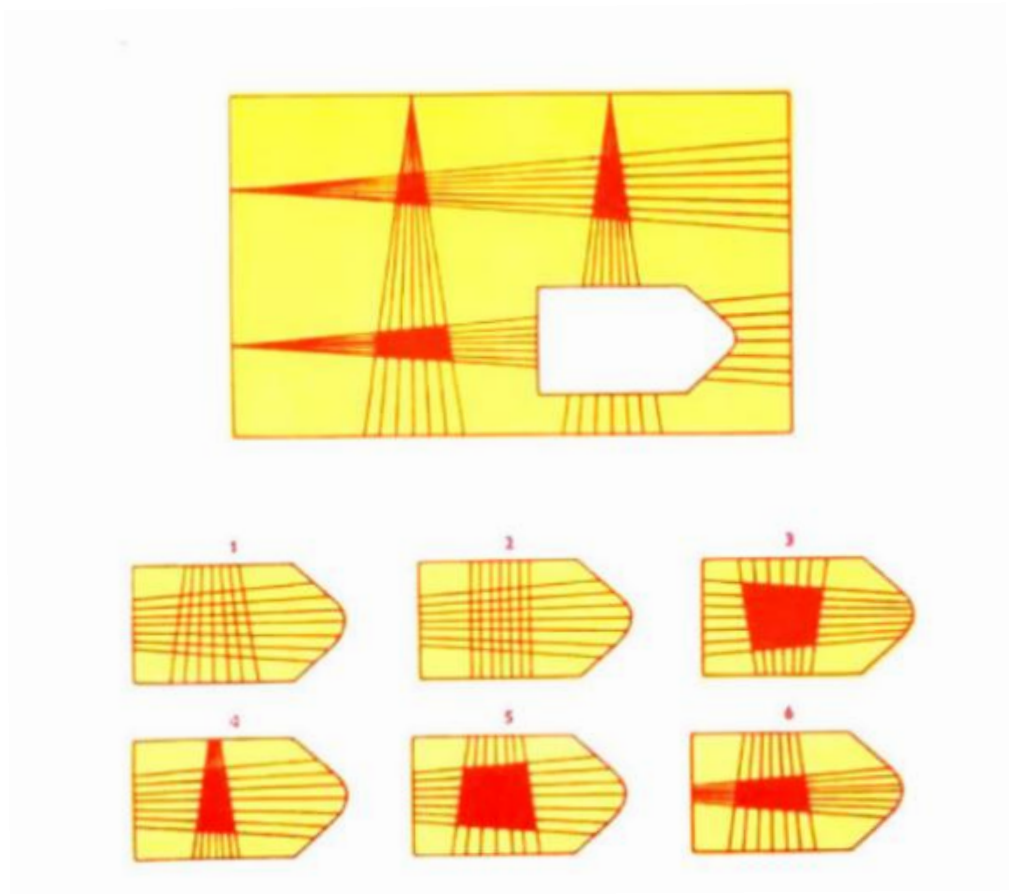

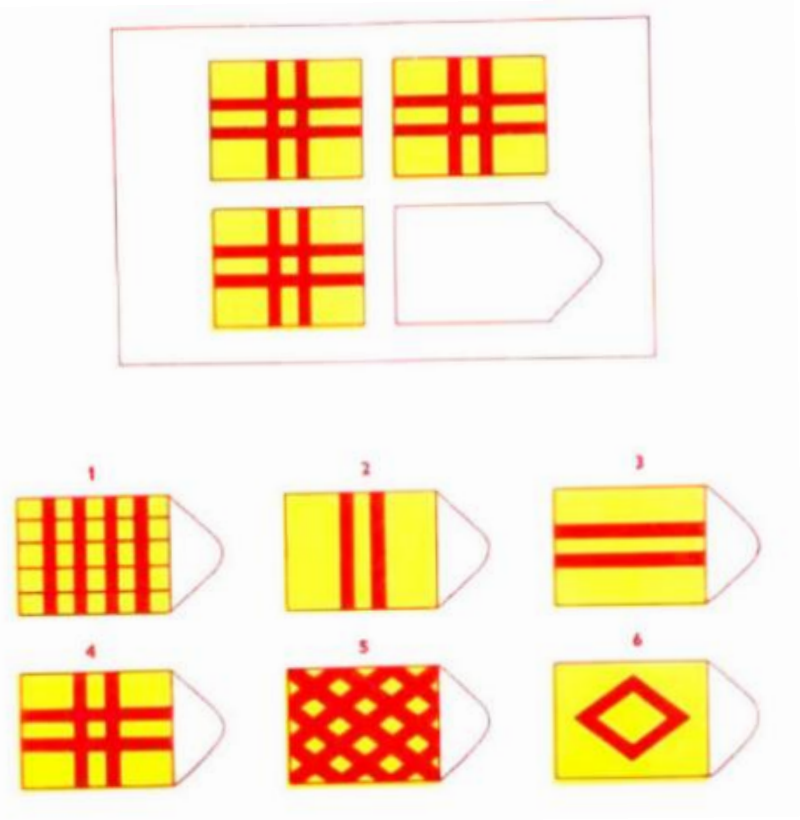

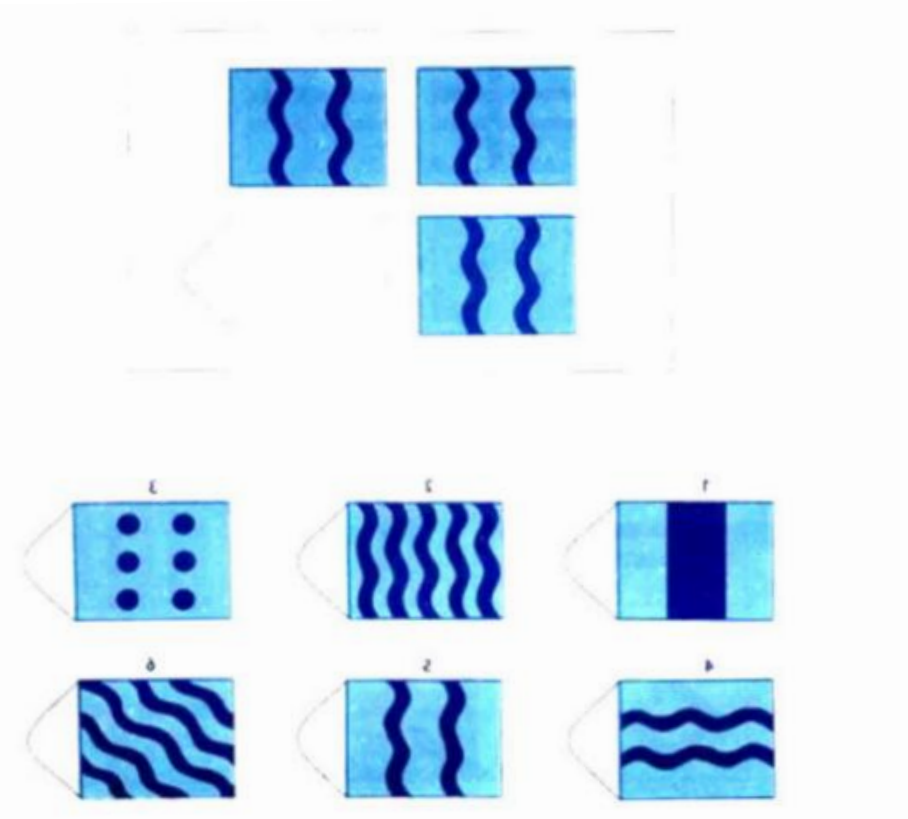

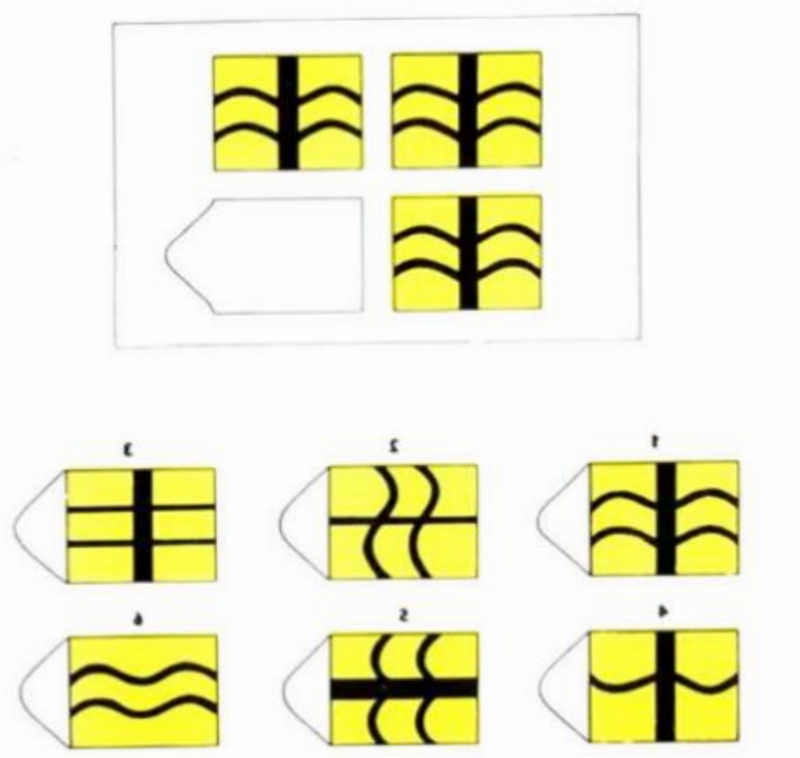

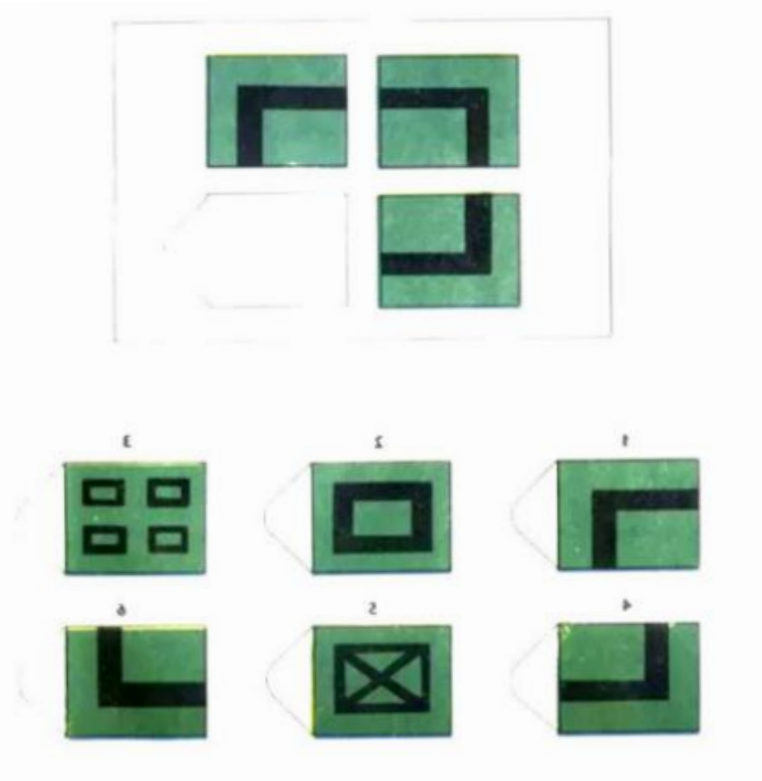

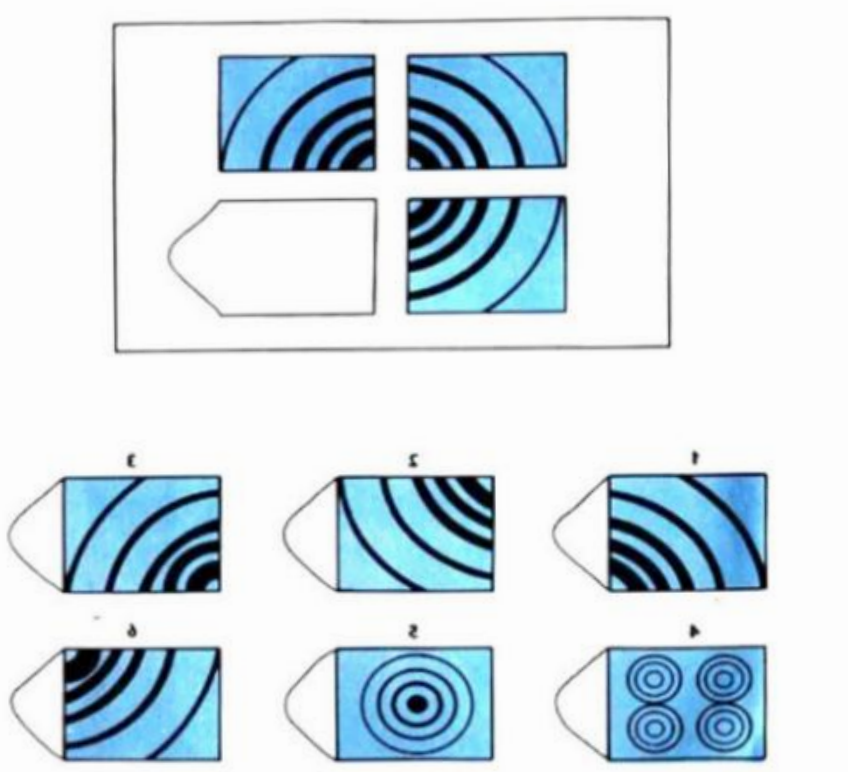

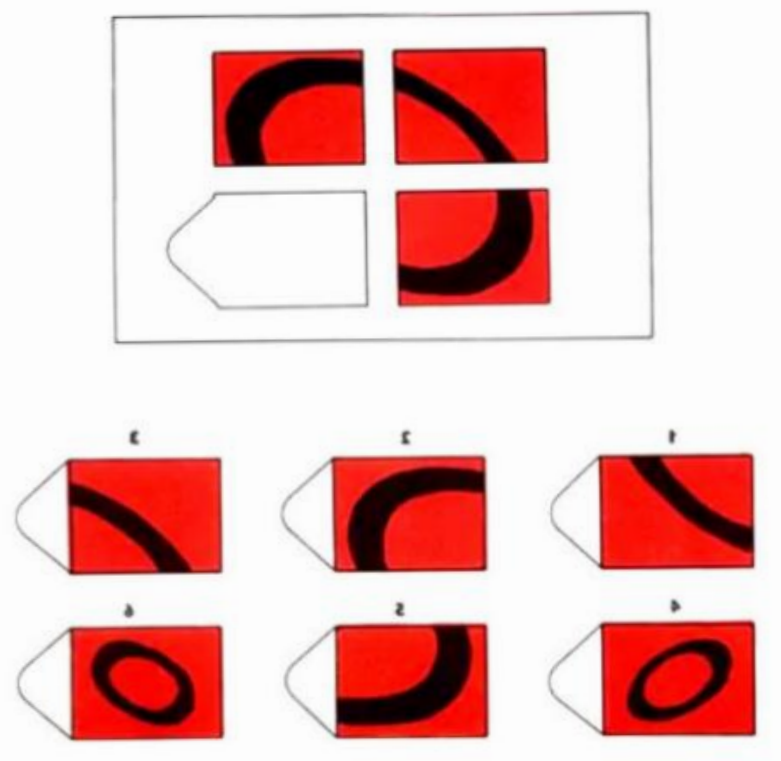

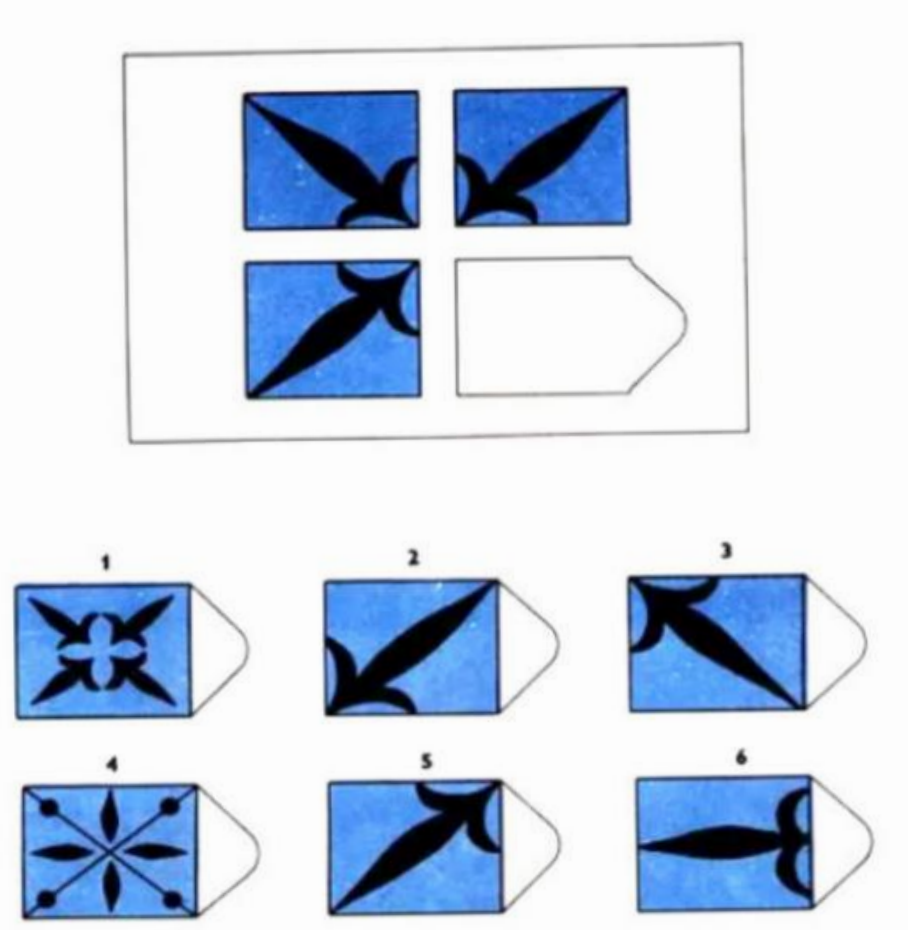

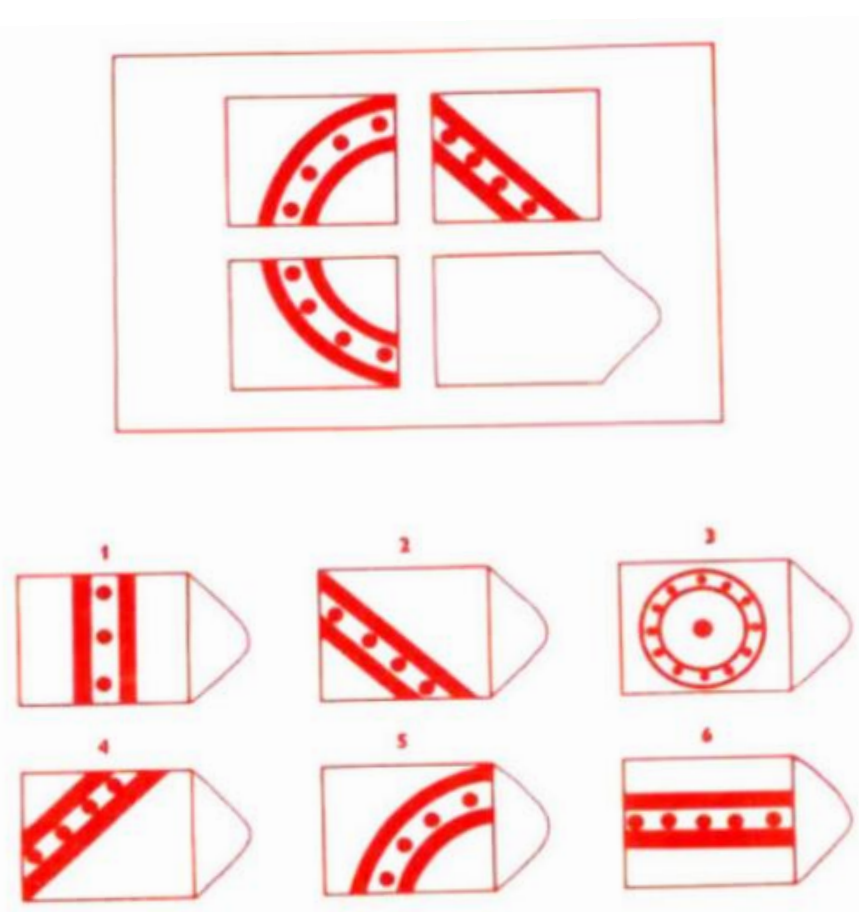

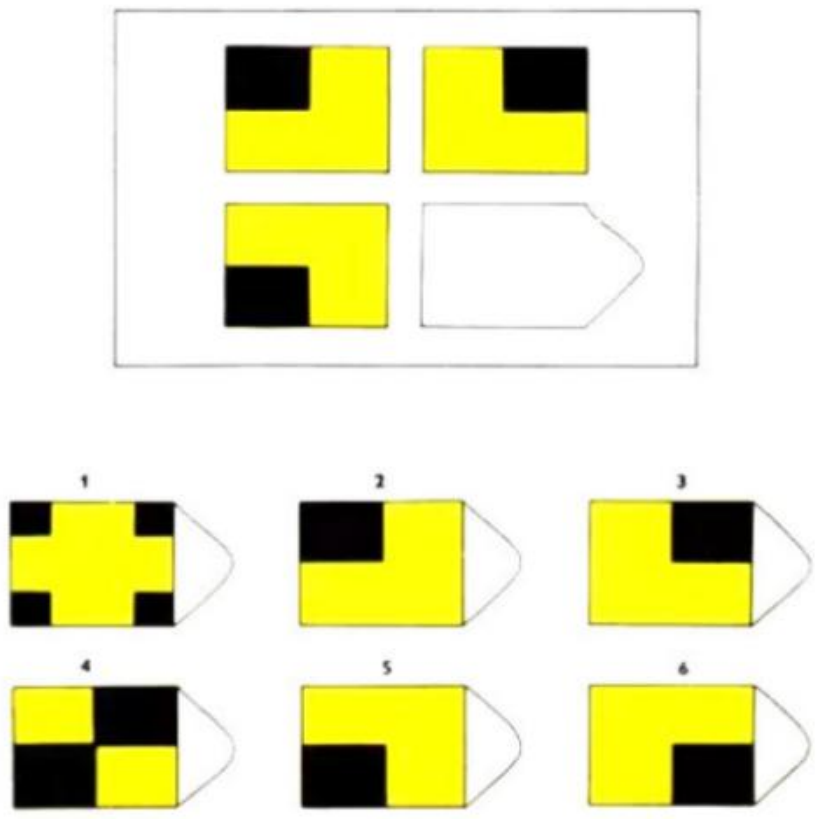

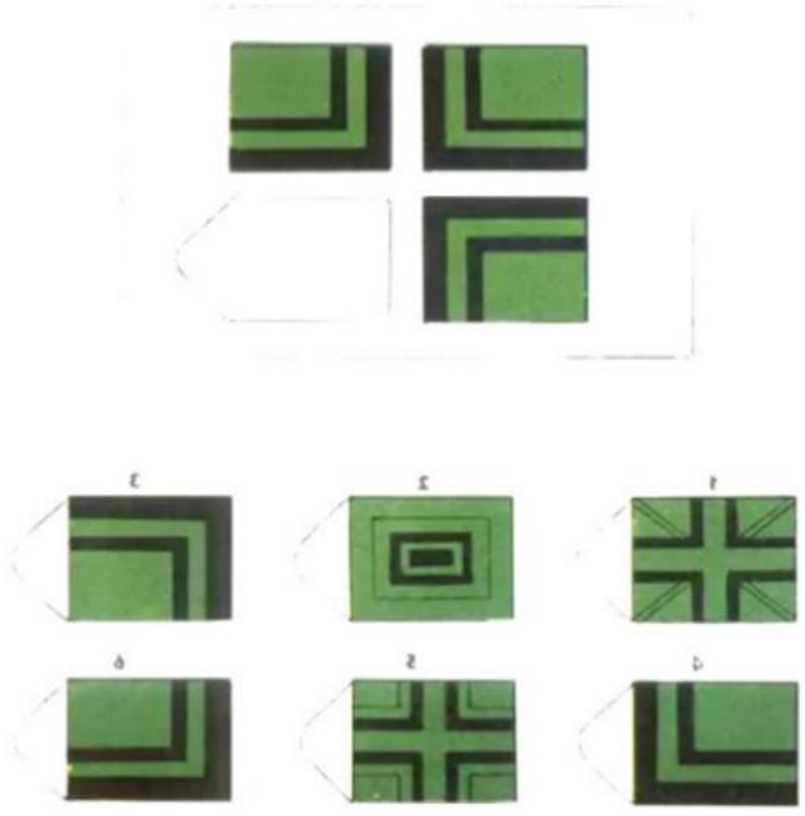

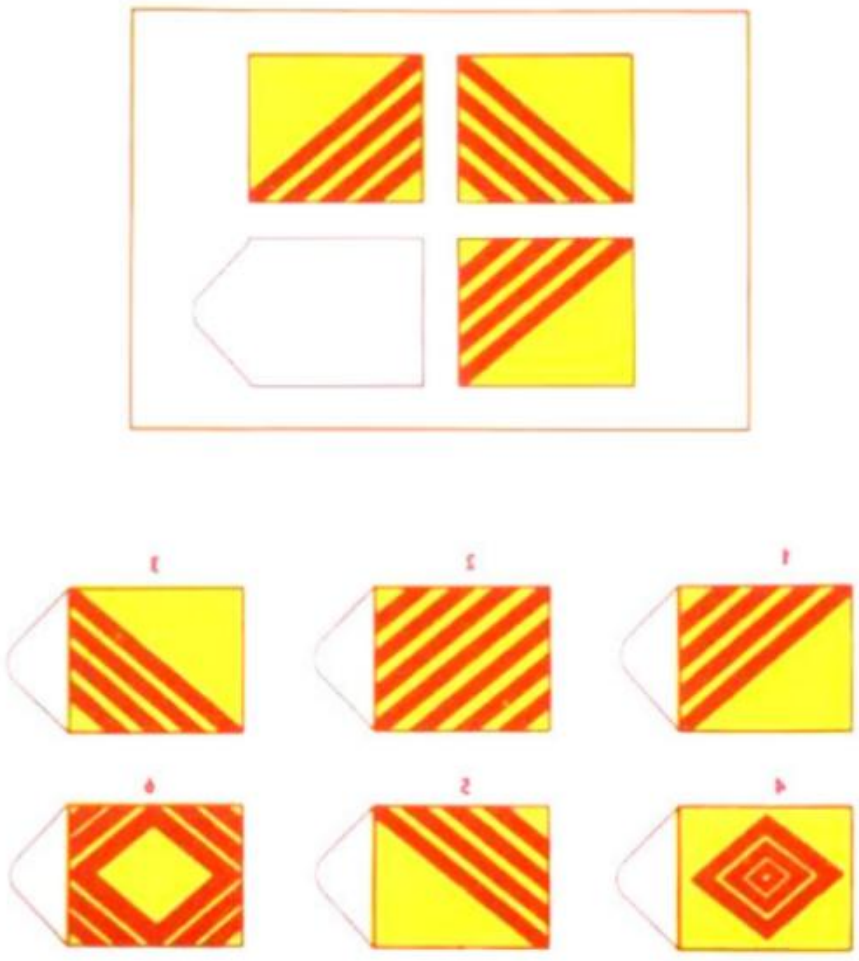

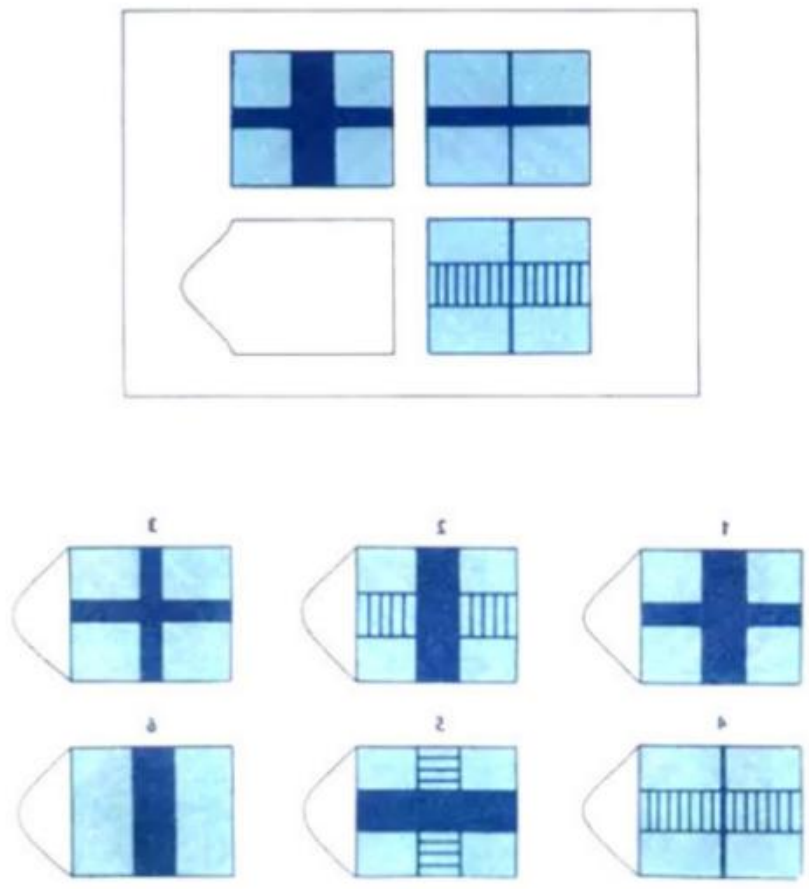

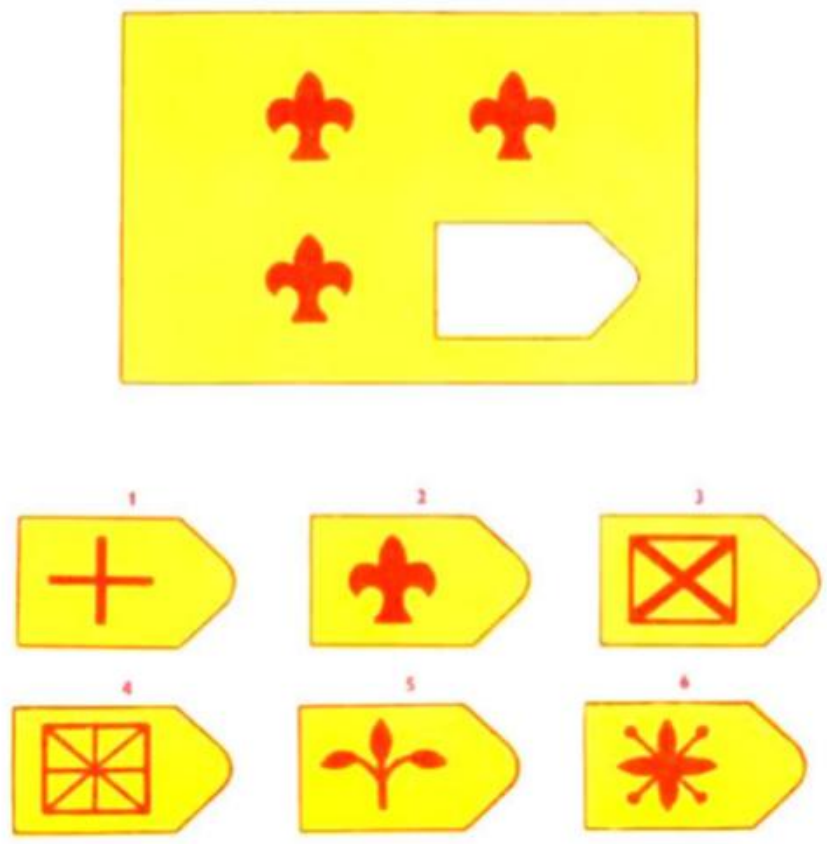

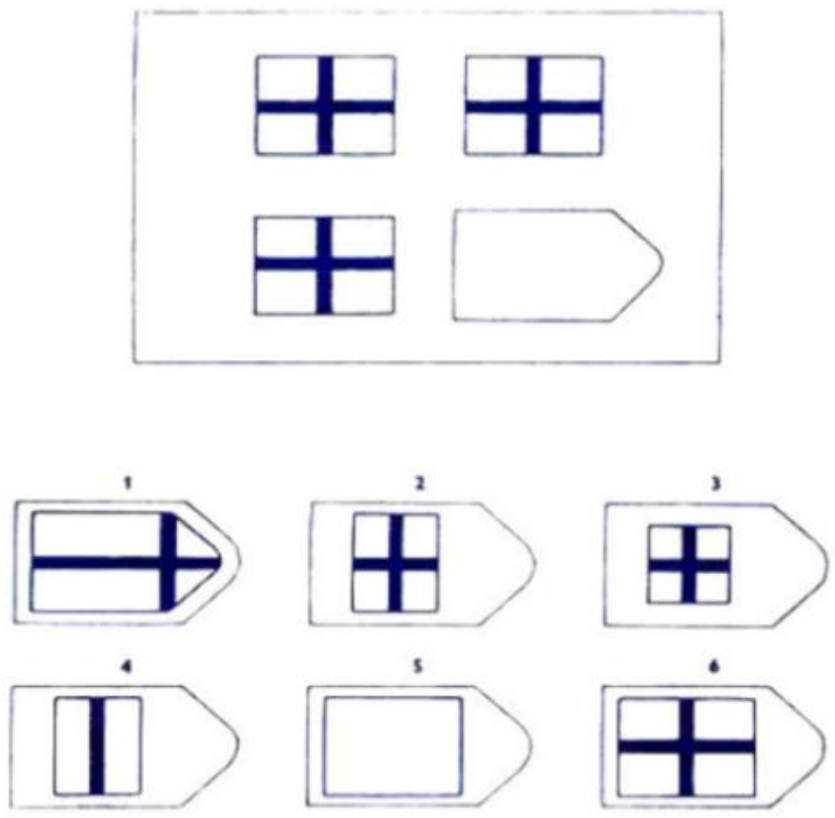

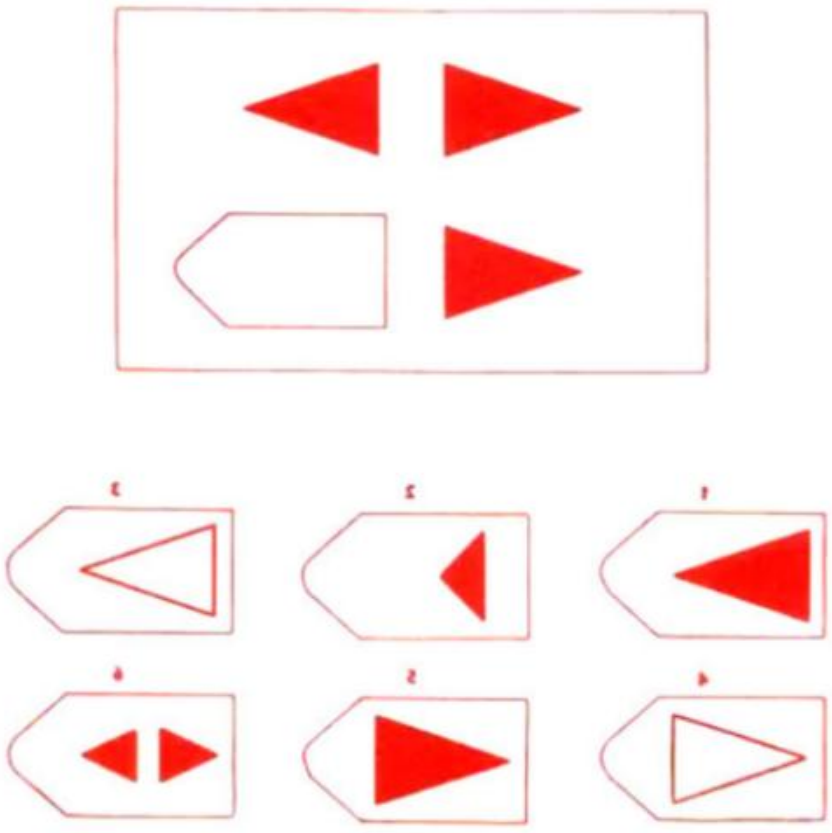

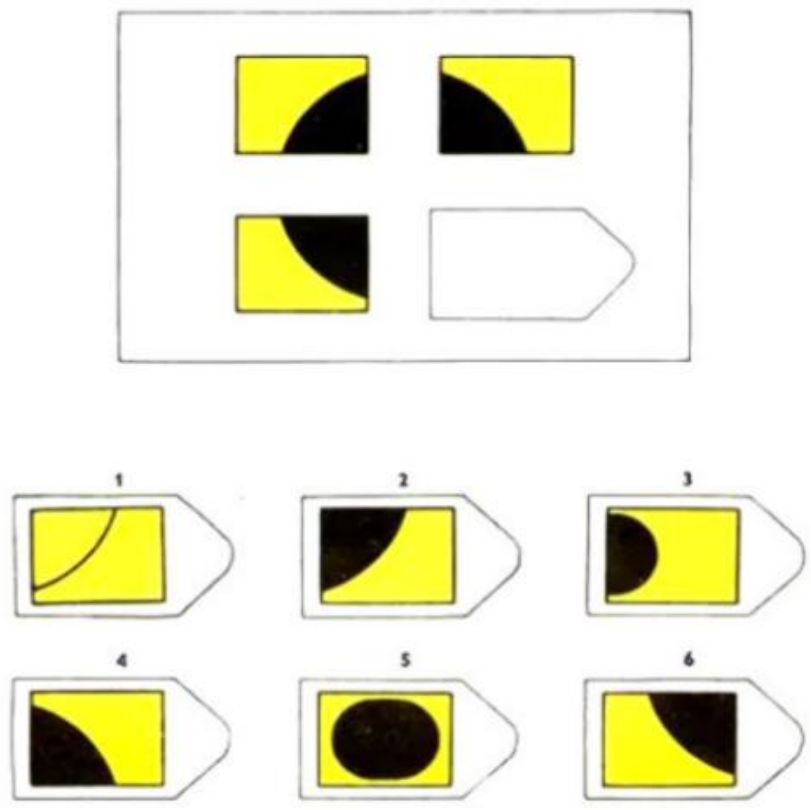

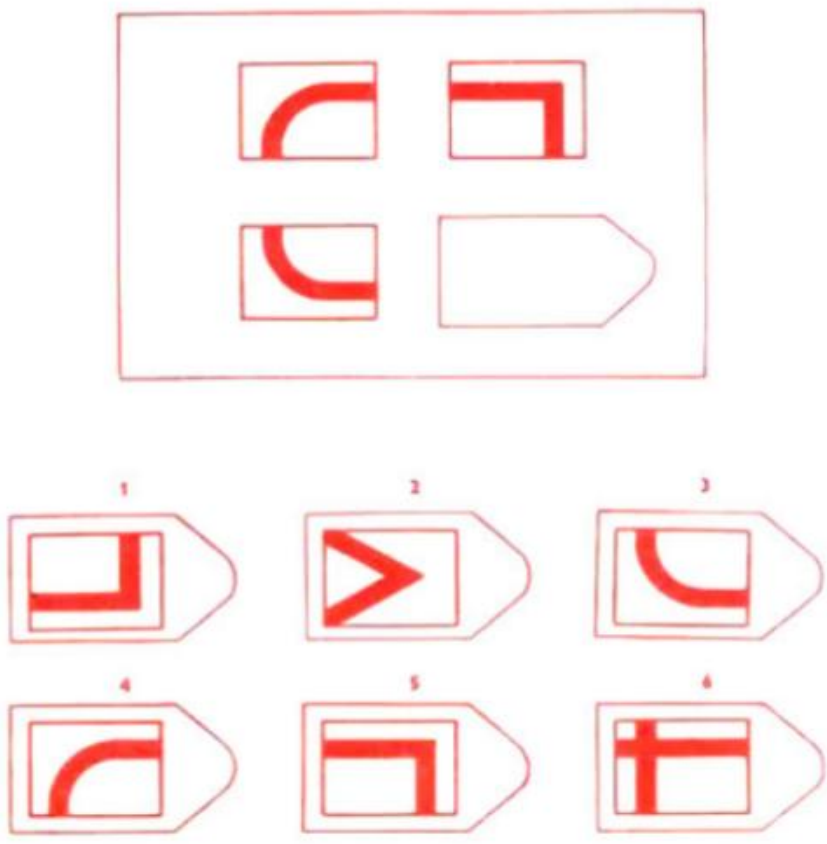

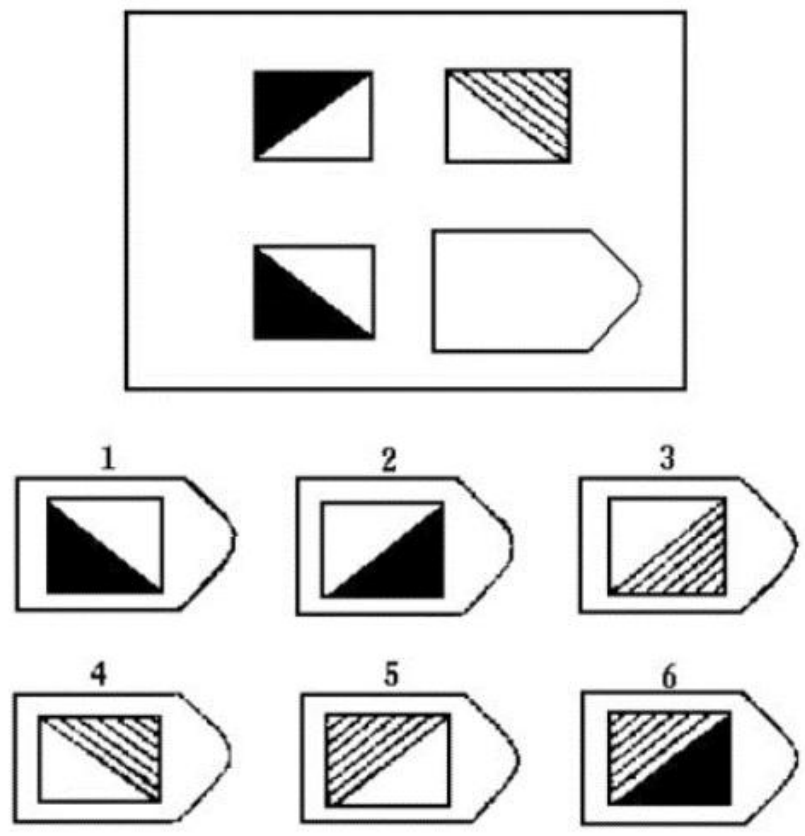
1
2
3
4
5
6

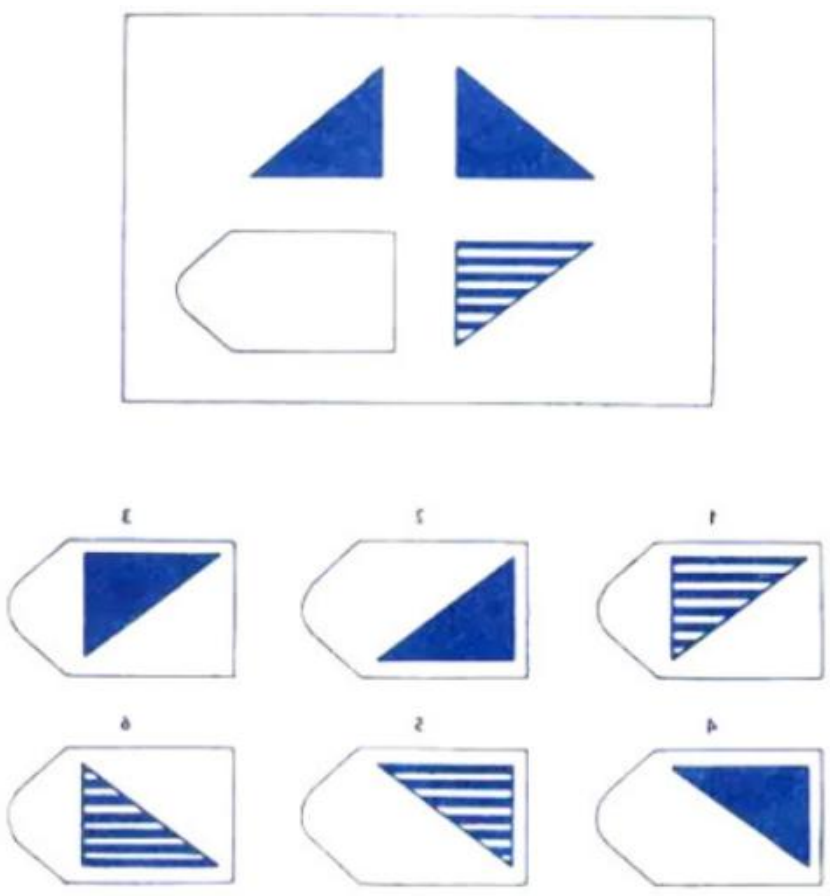

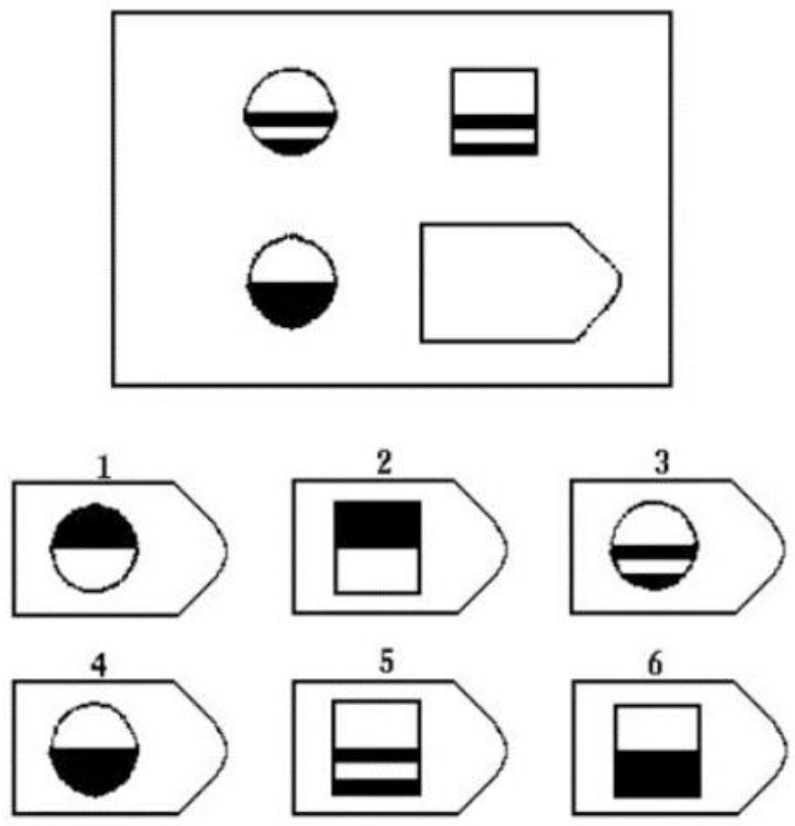
1
2
3
4
5
6

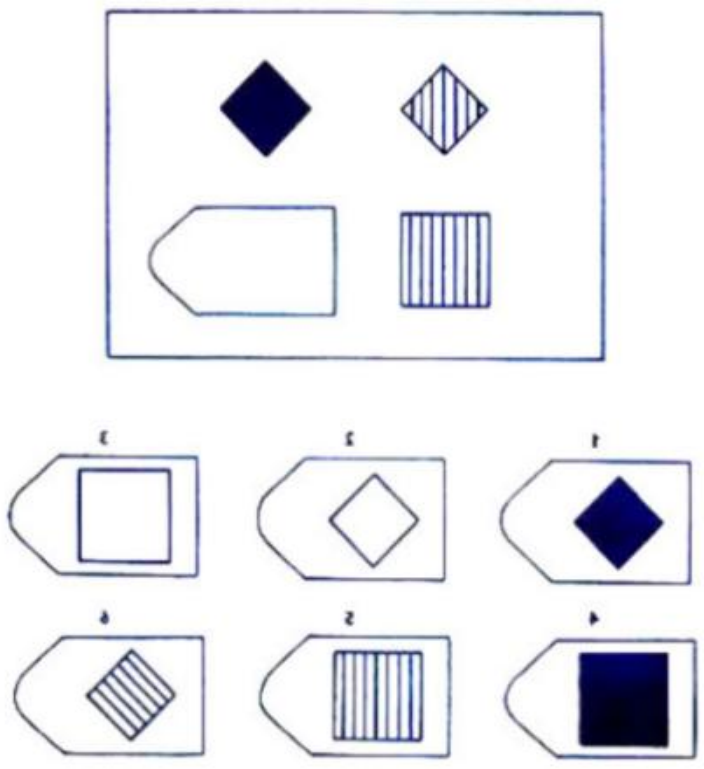

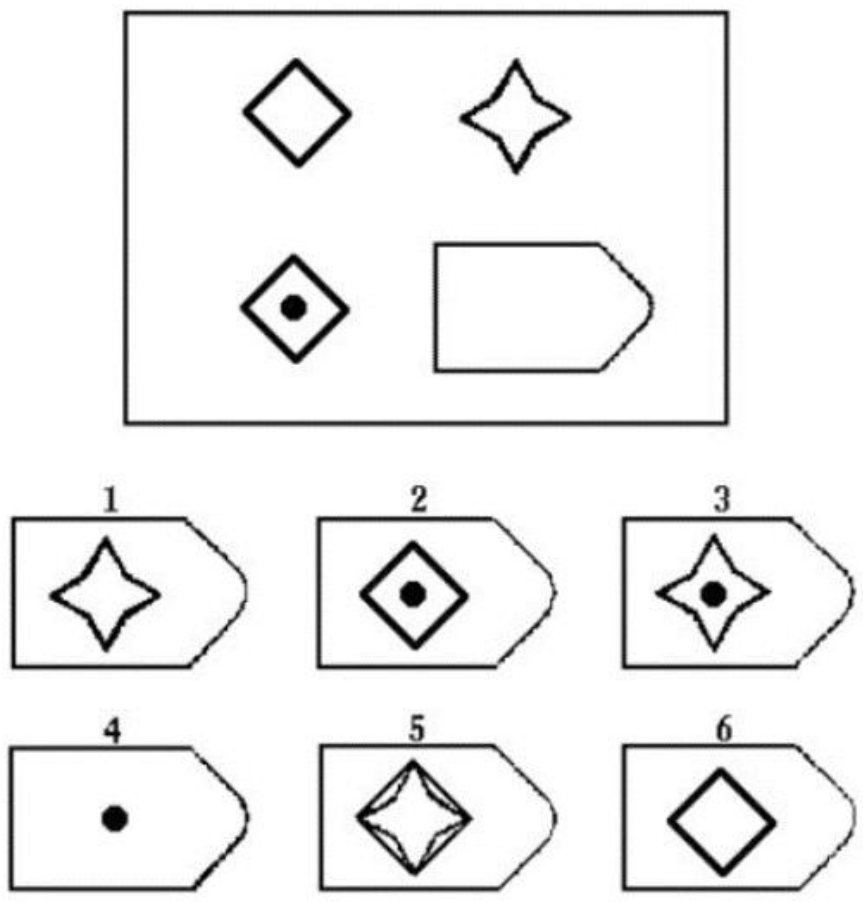
1
2
3
4
5
6

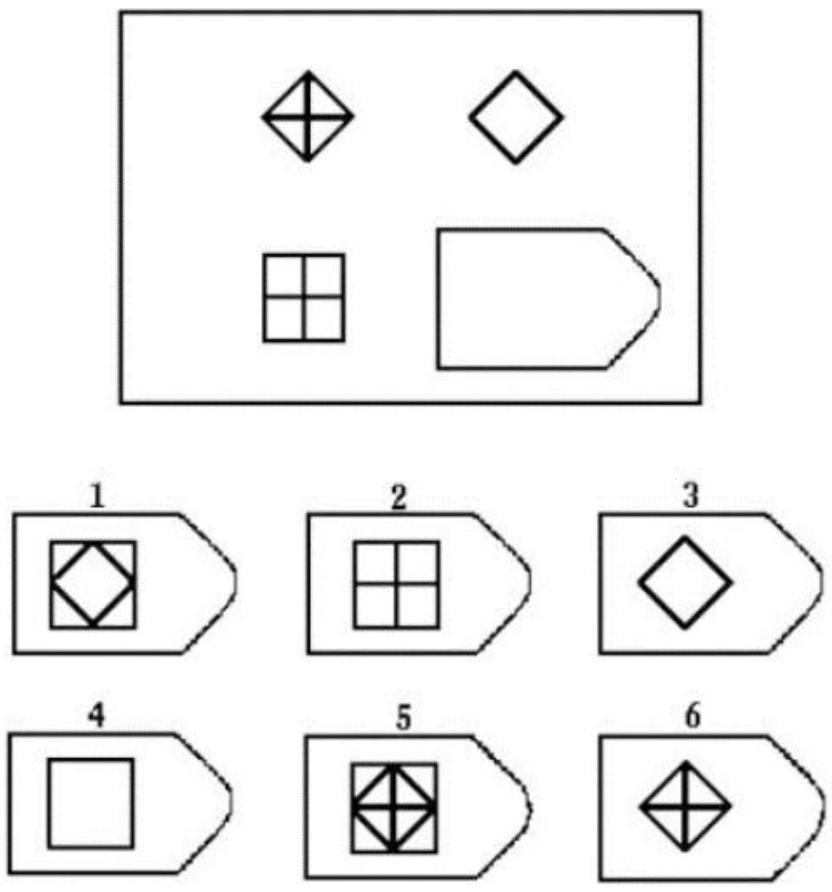
1
2
3
4
5
6

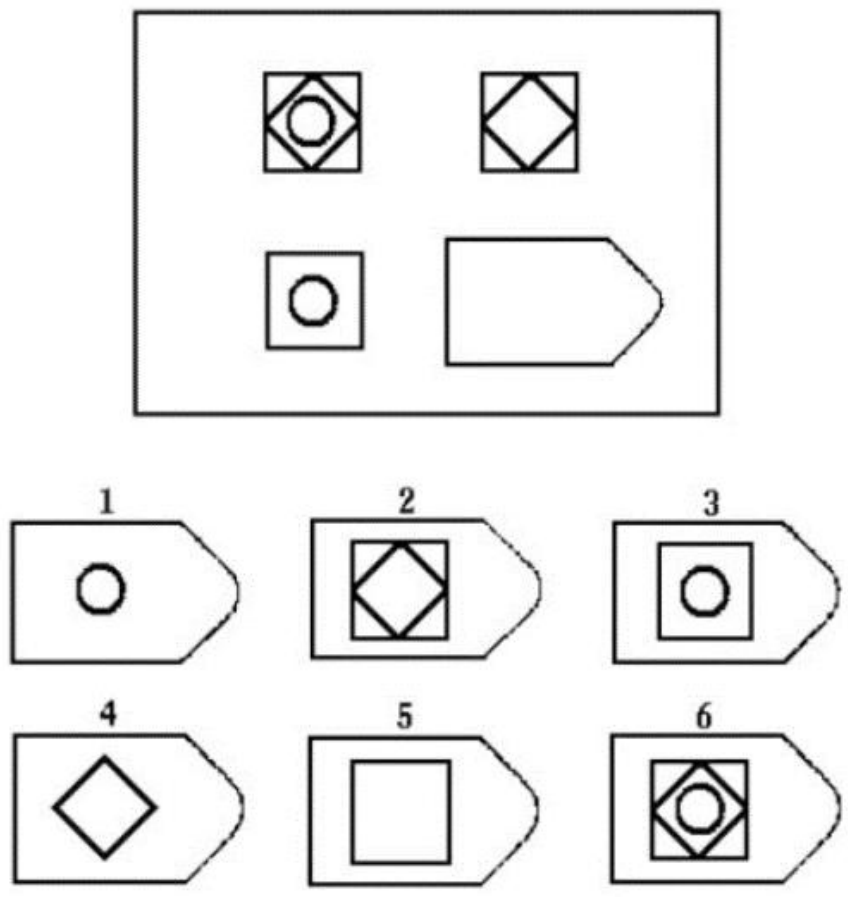
1
2
3
4
5
6

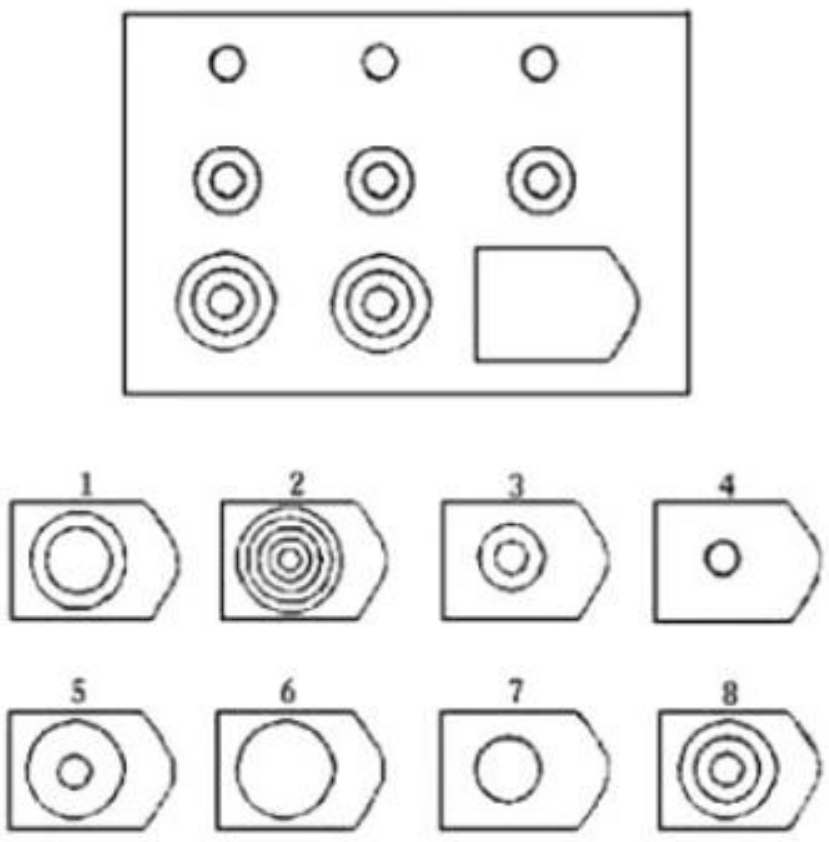

C1

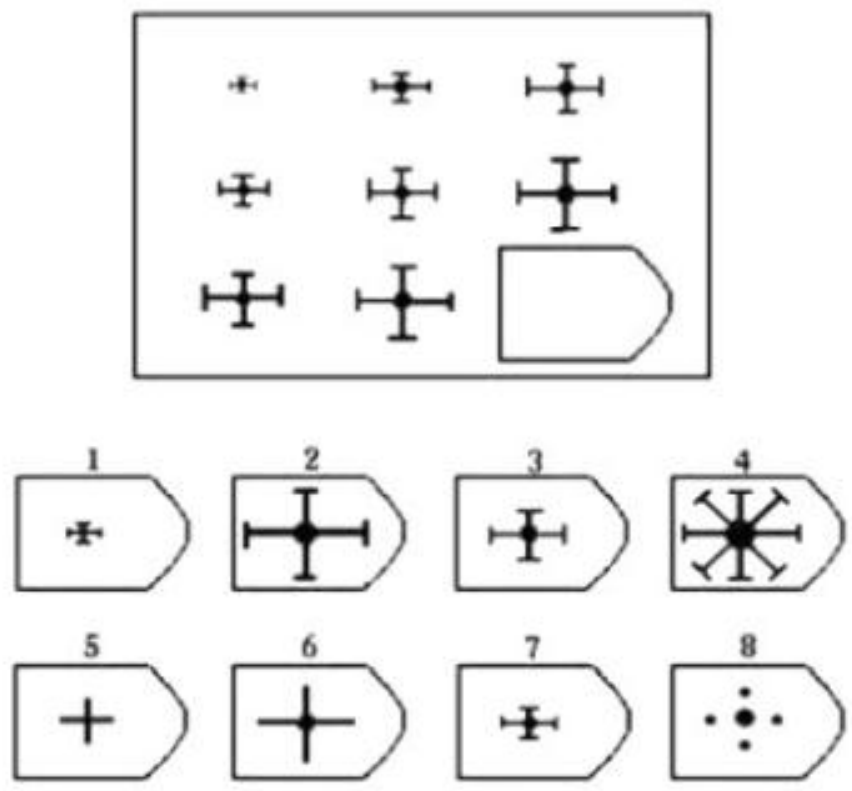

C2

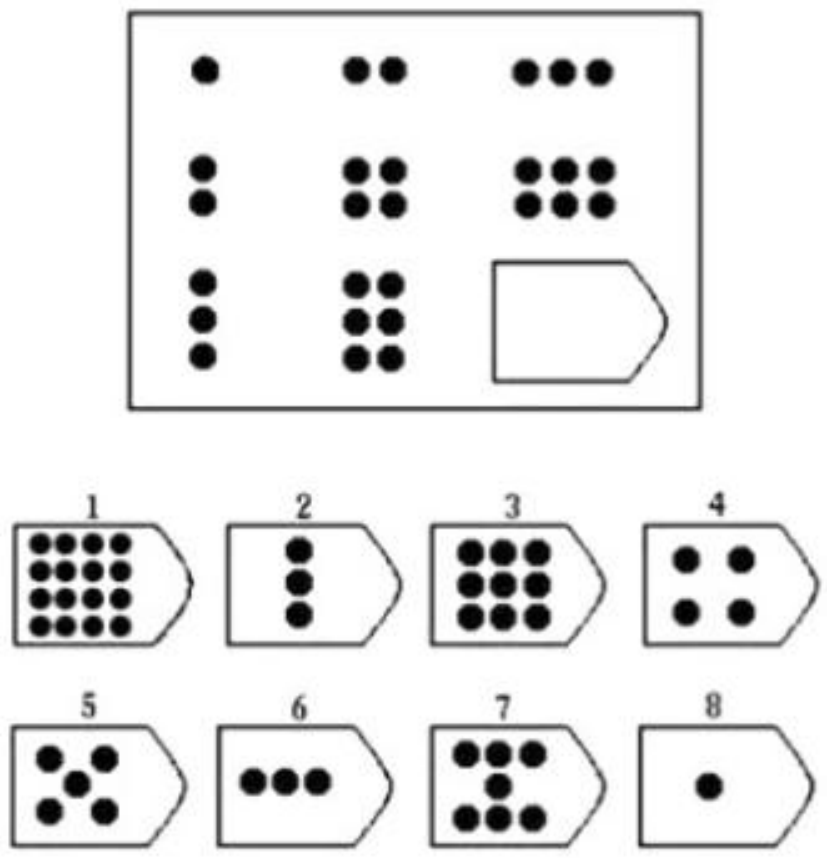

C3

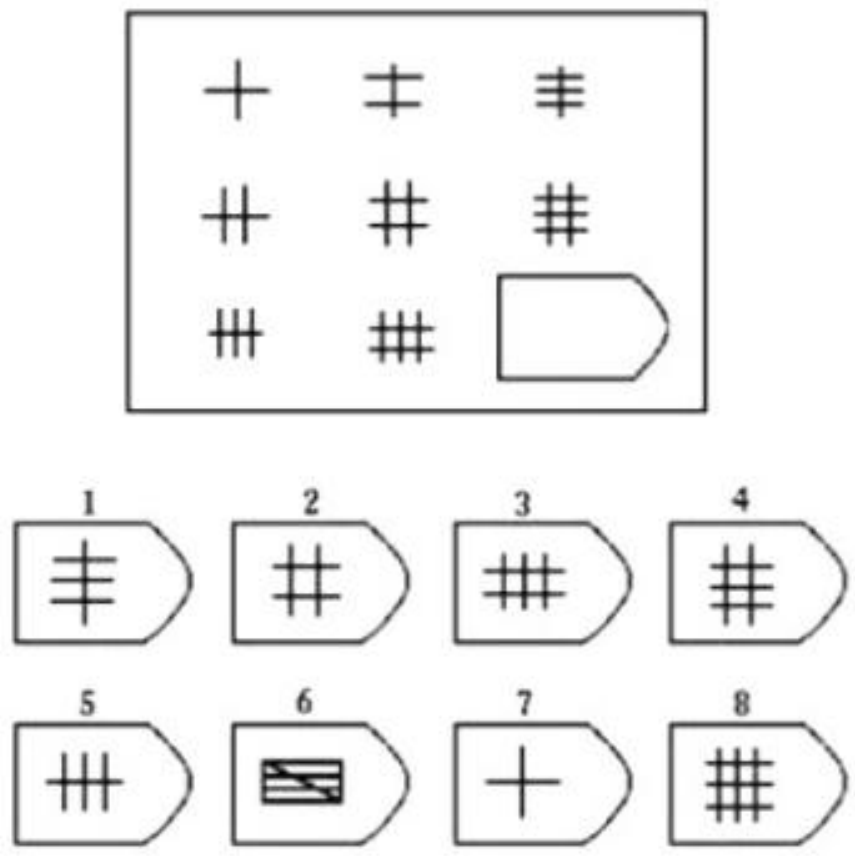

C4

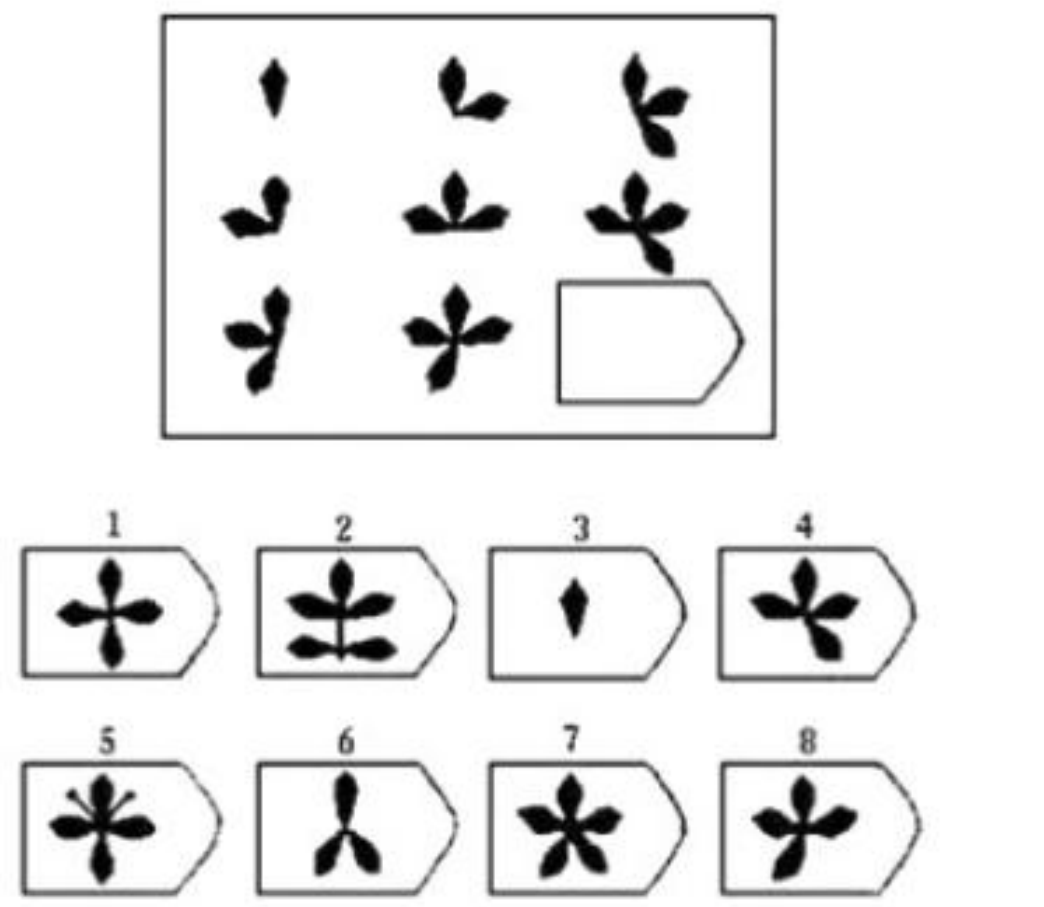

C5

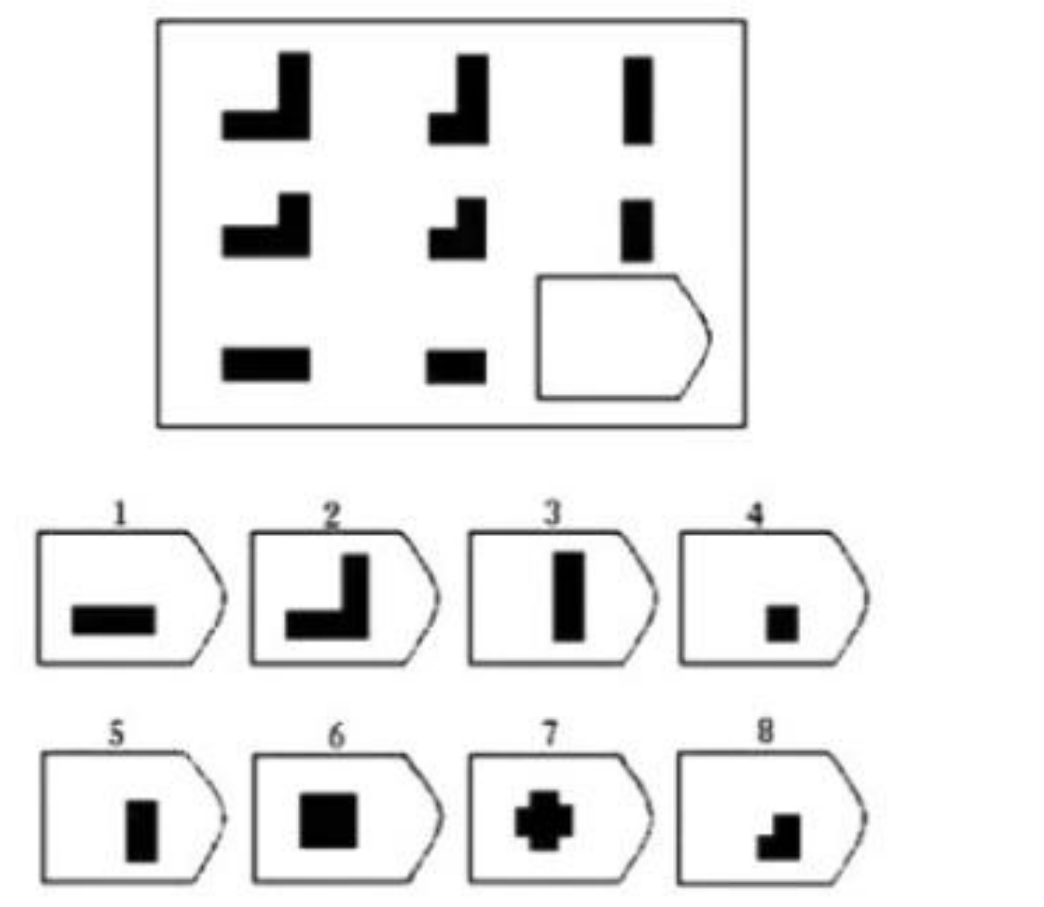

C6

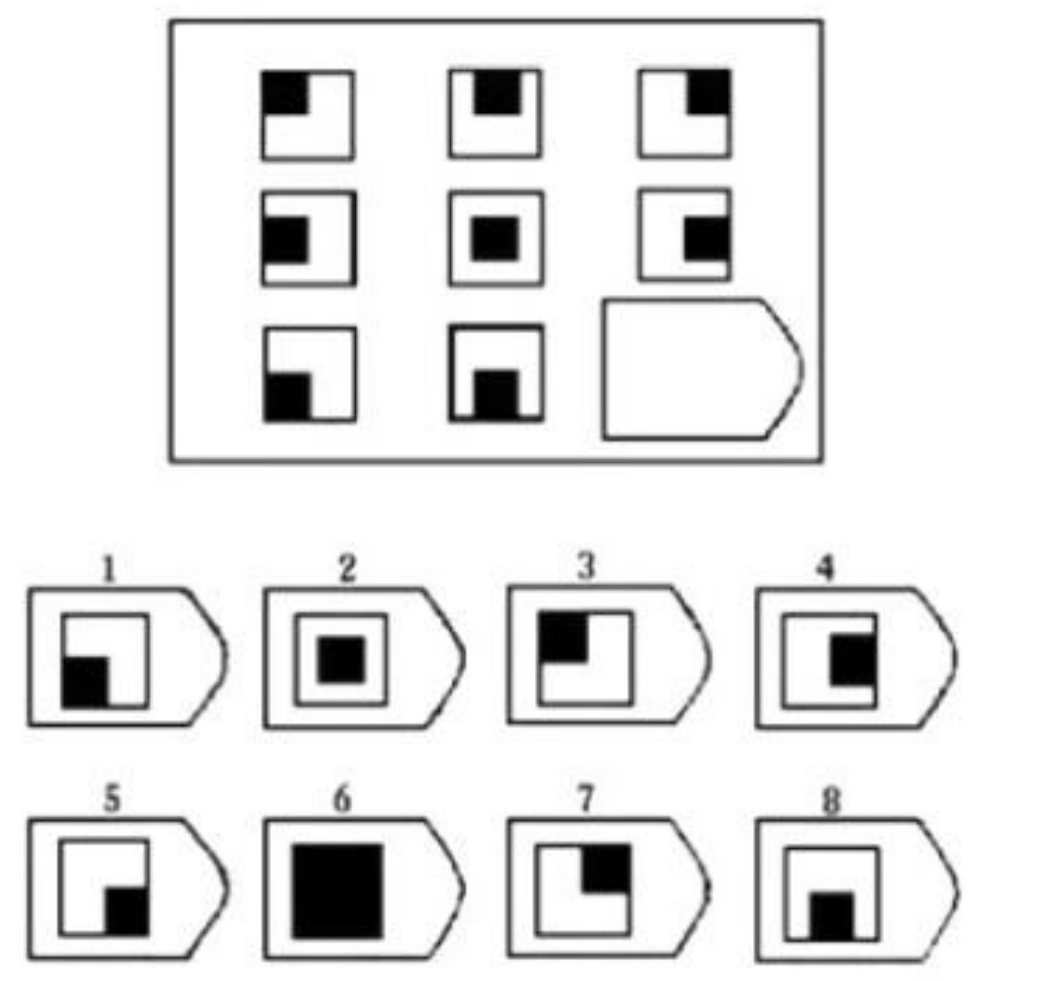

C7

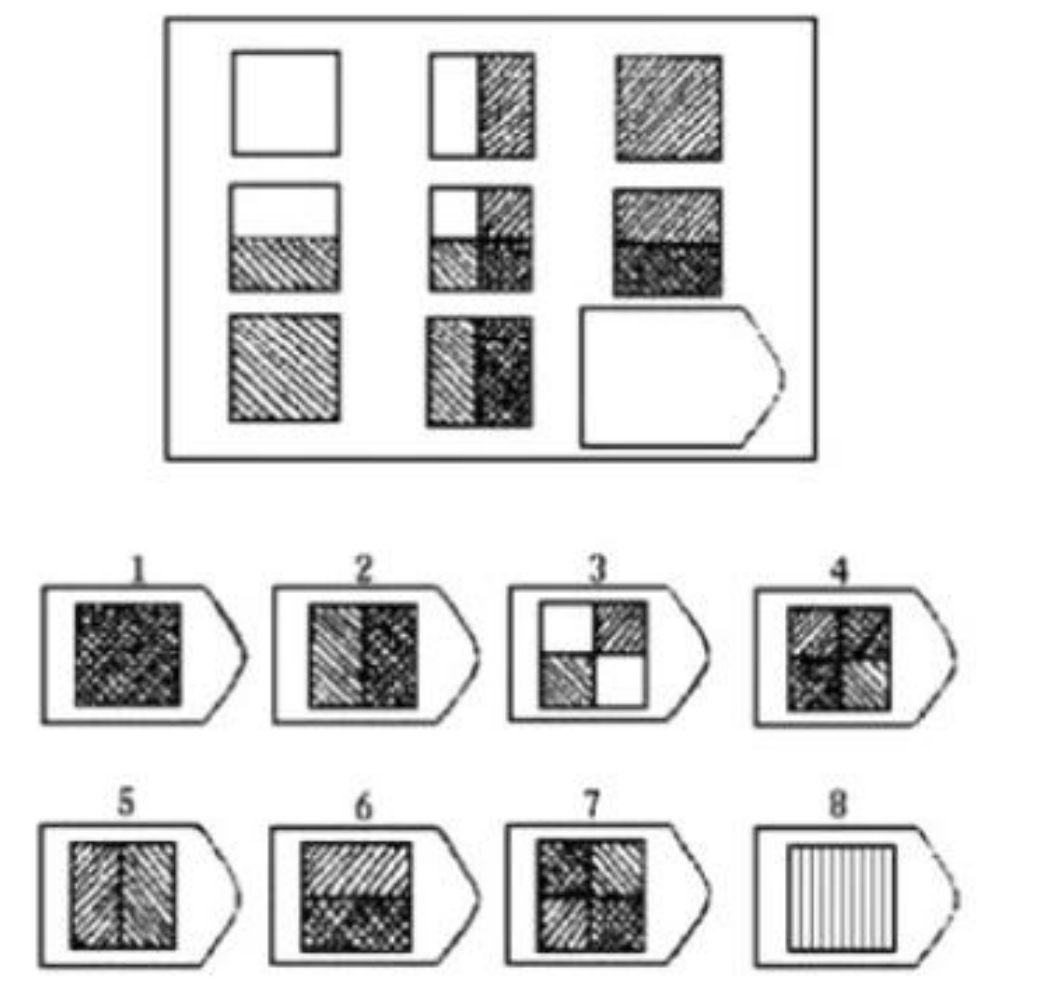

C8

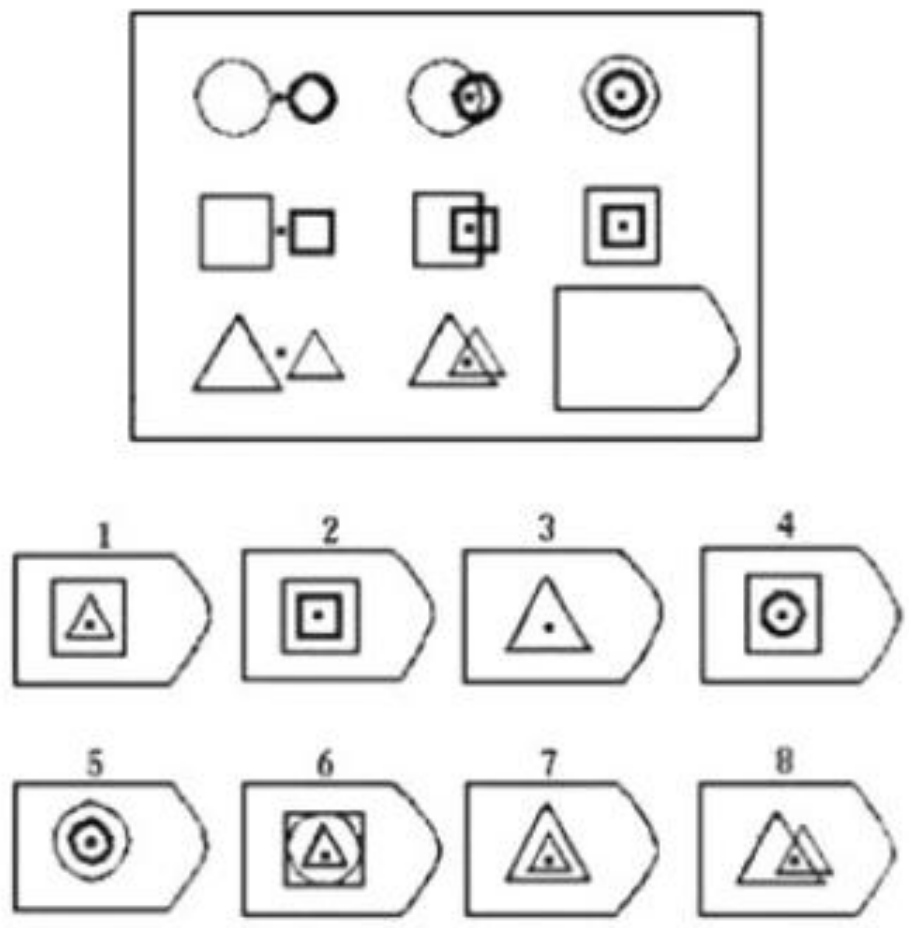

C9

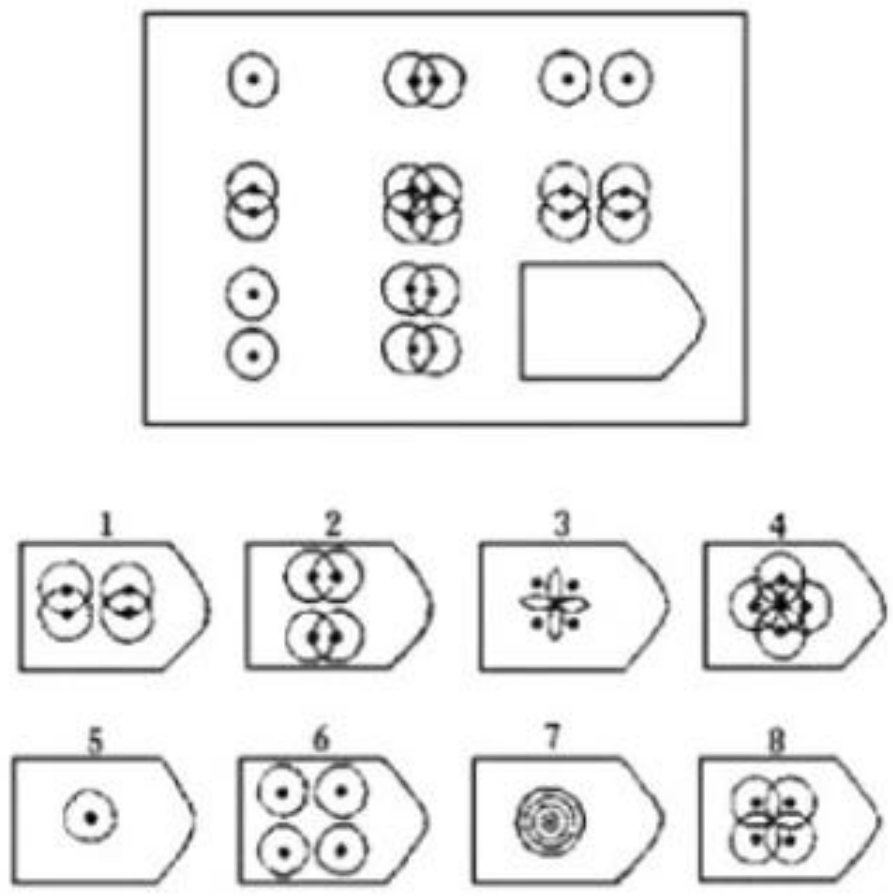

C10

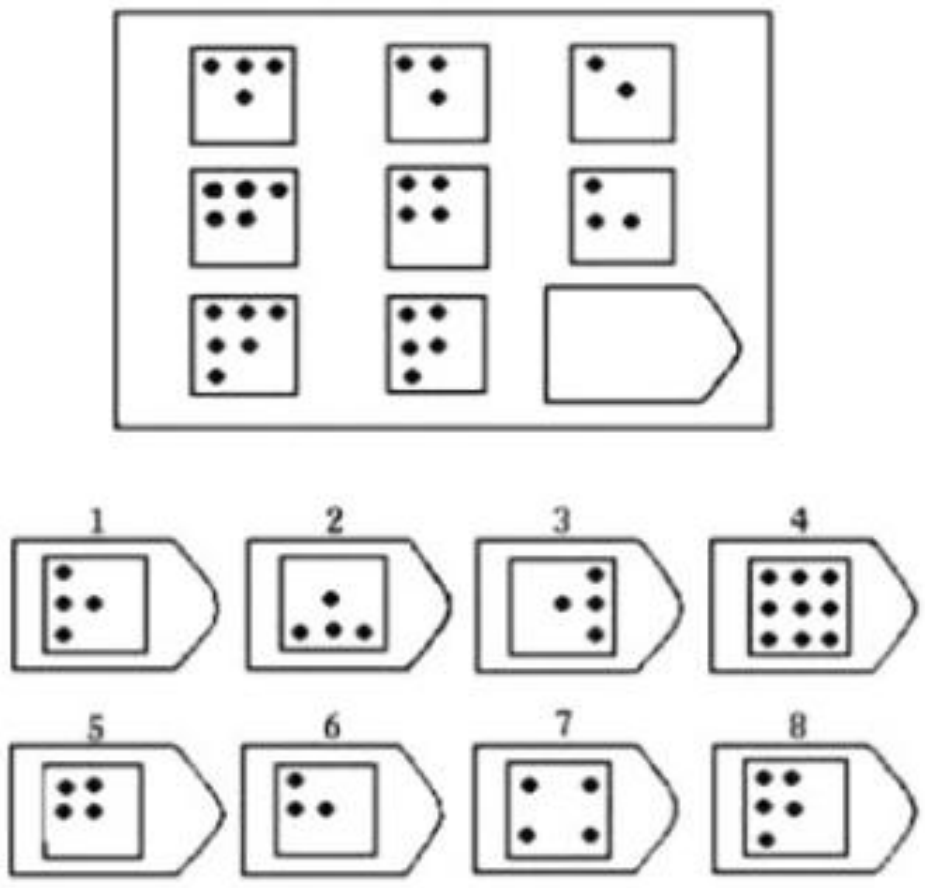

C11

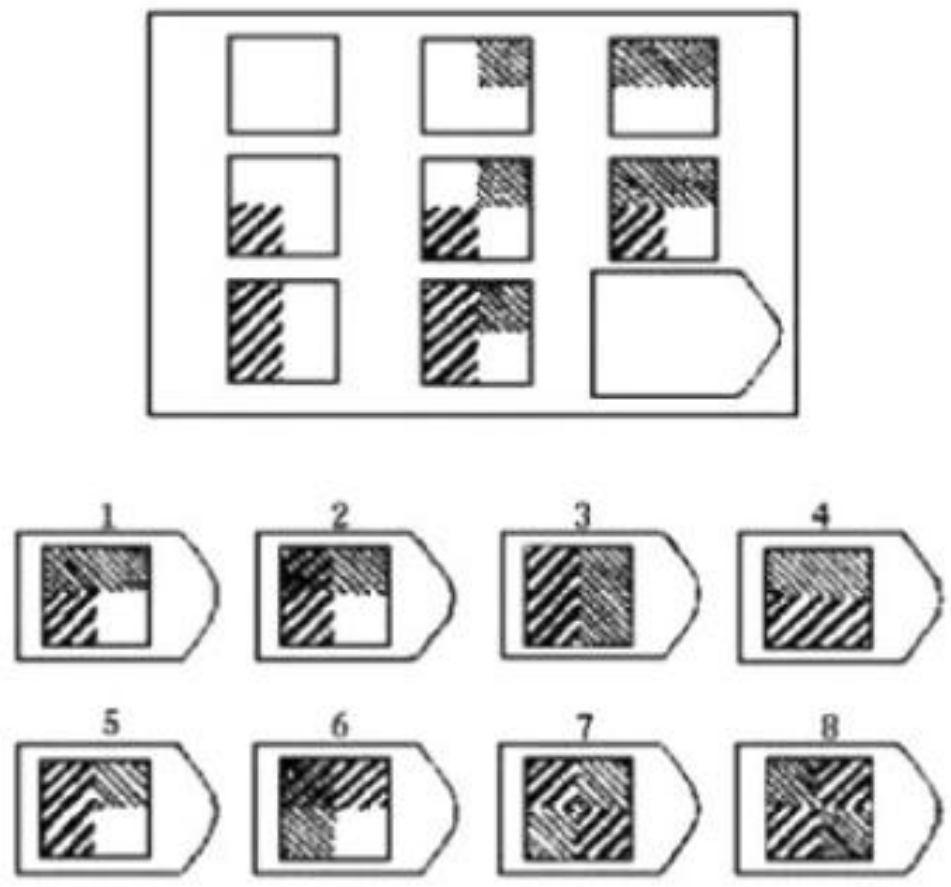

C12

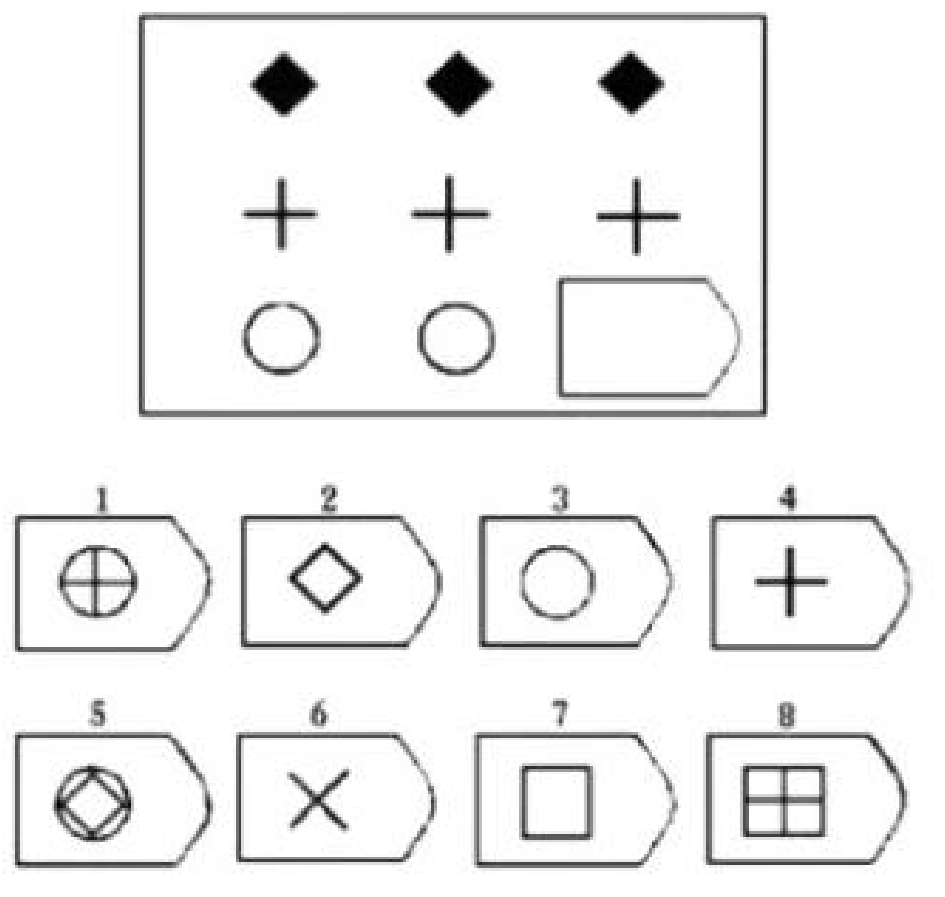

D1

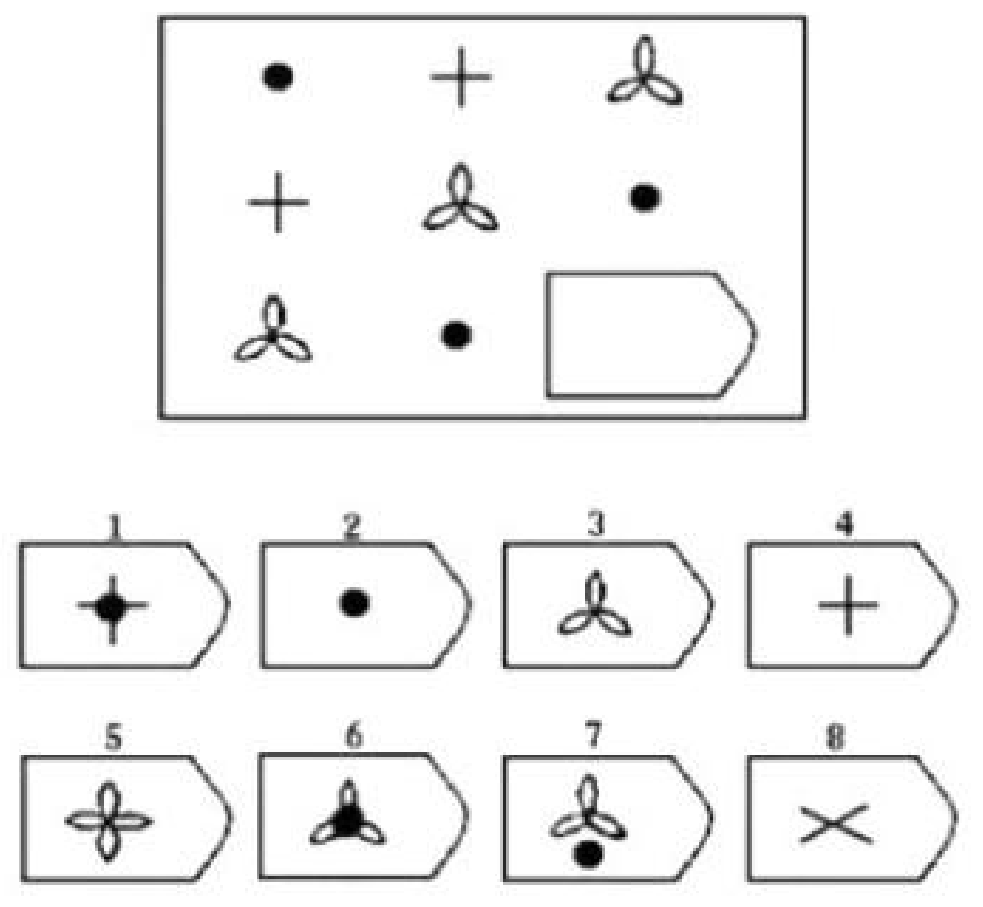

D2

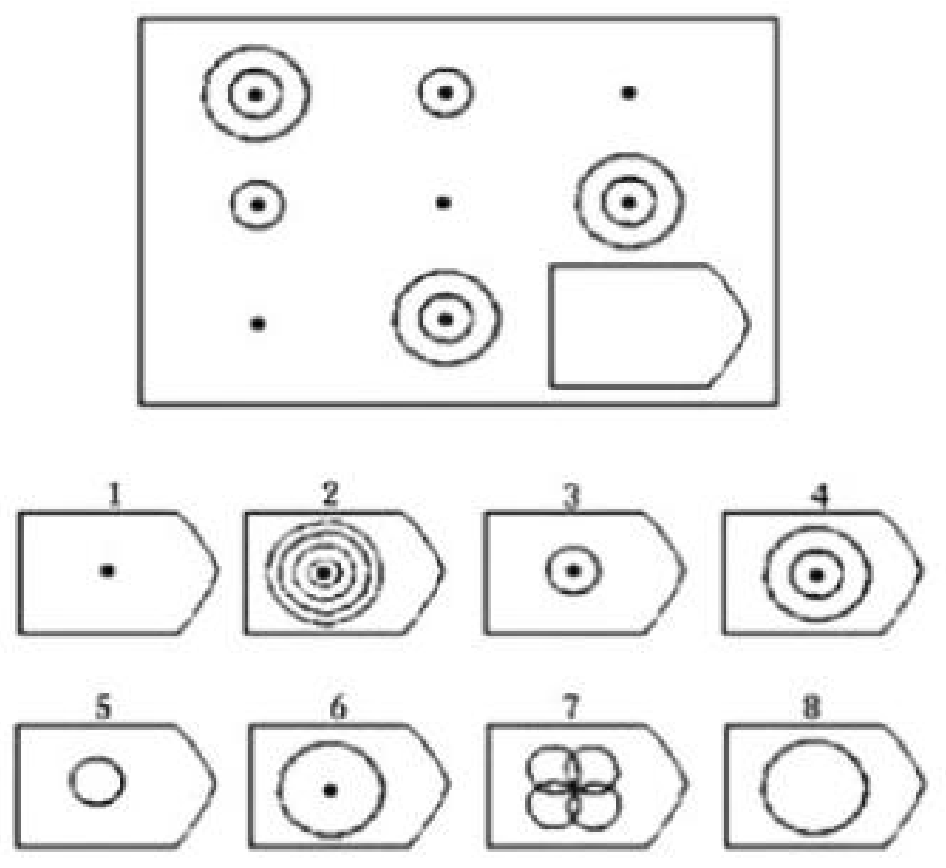

D3

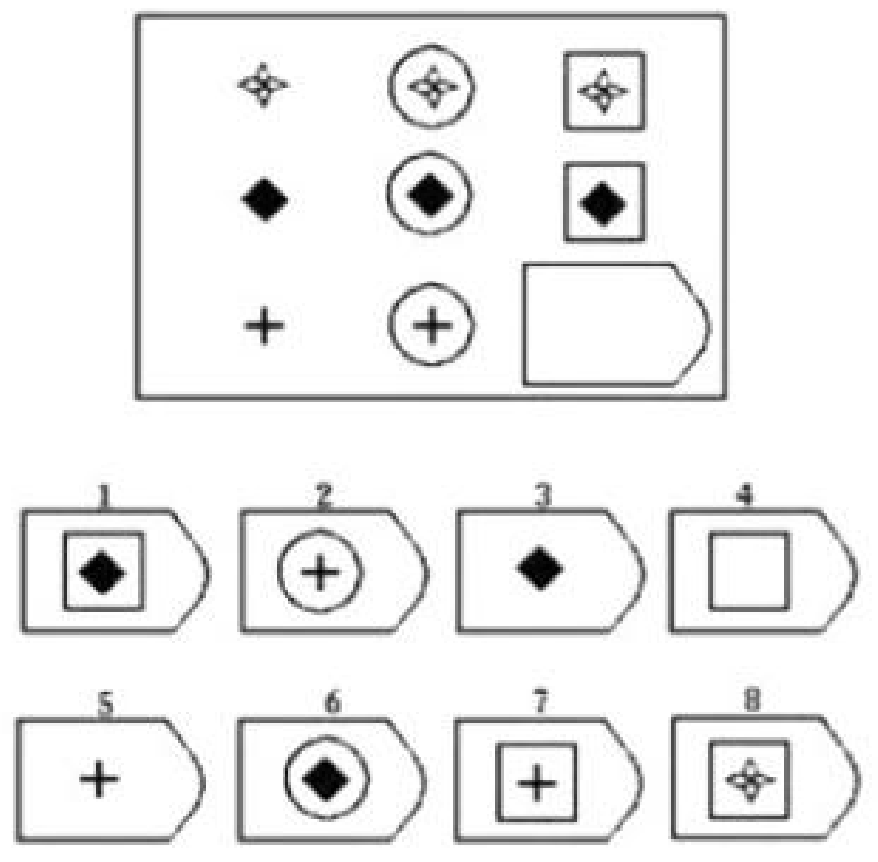

D4

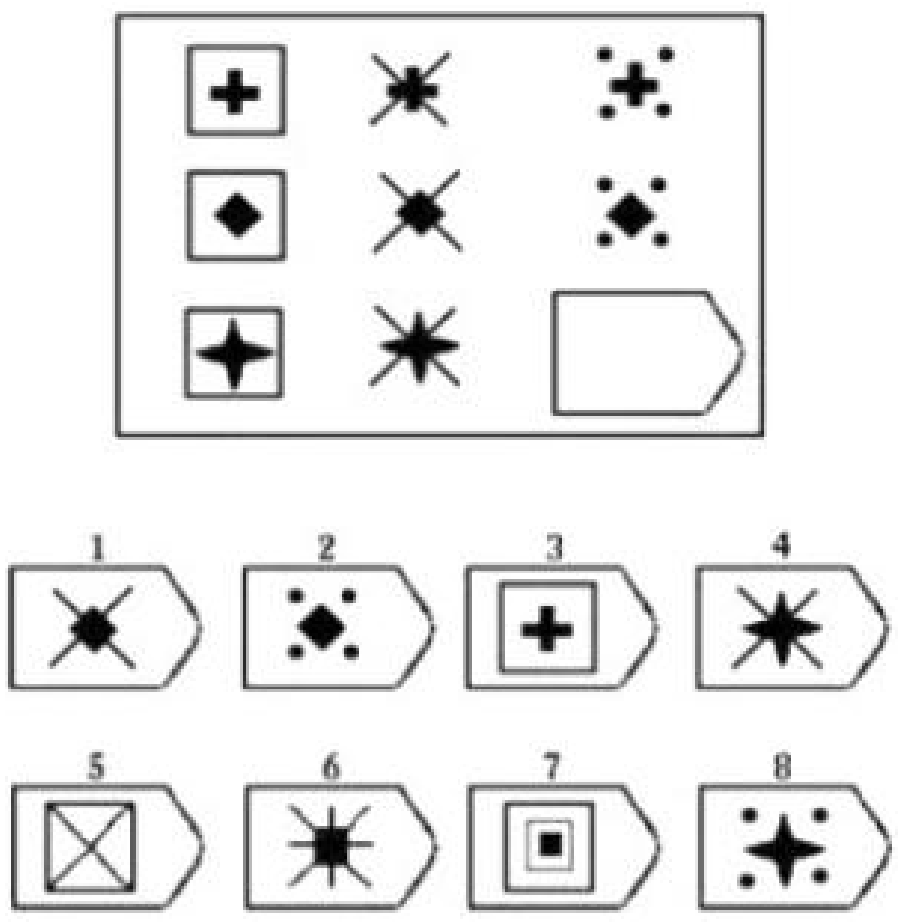

D5

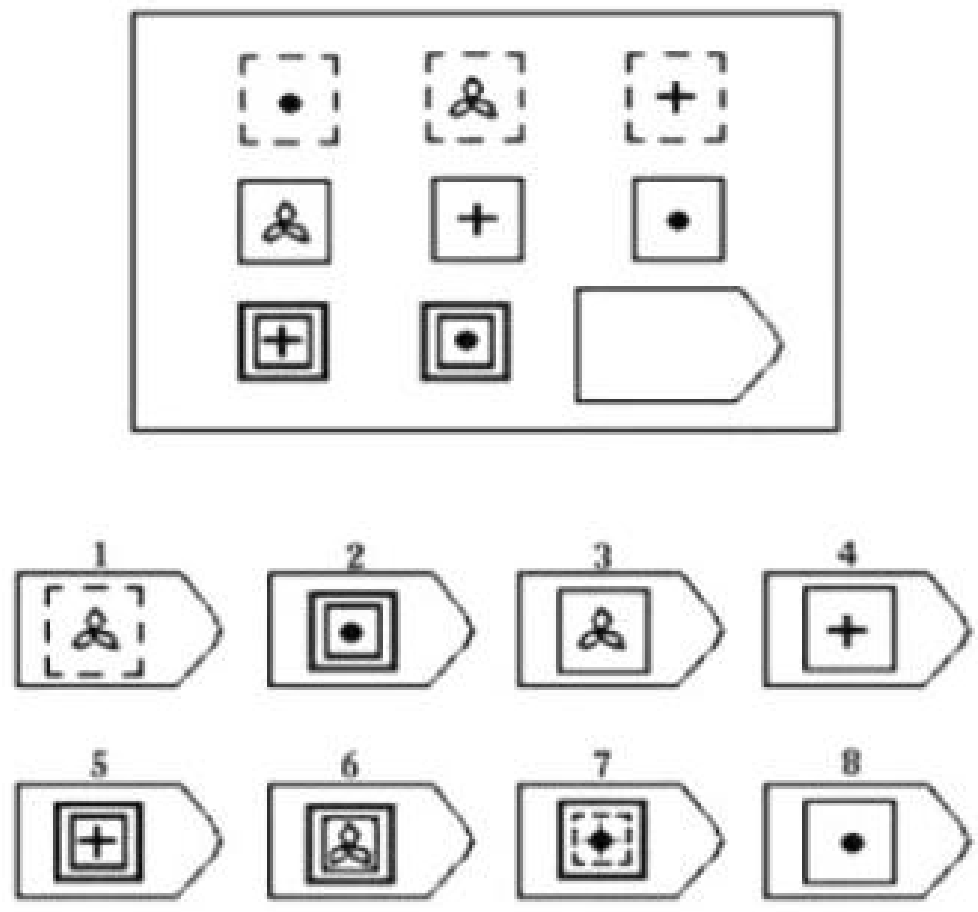

D6

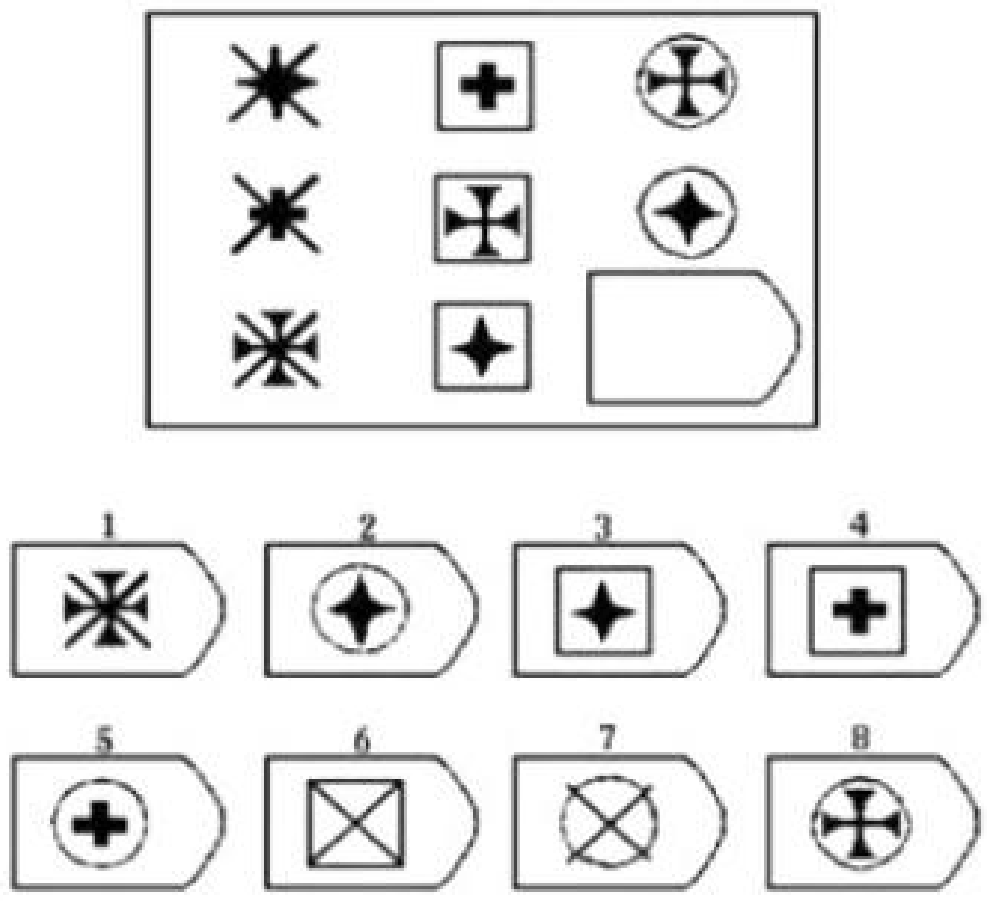

D7

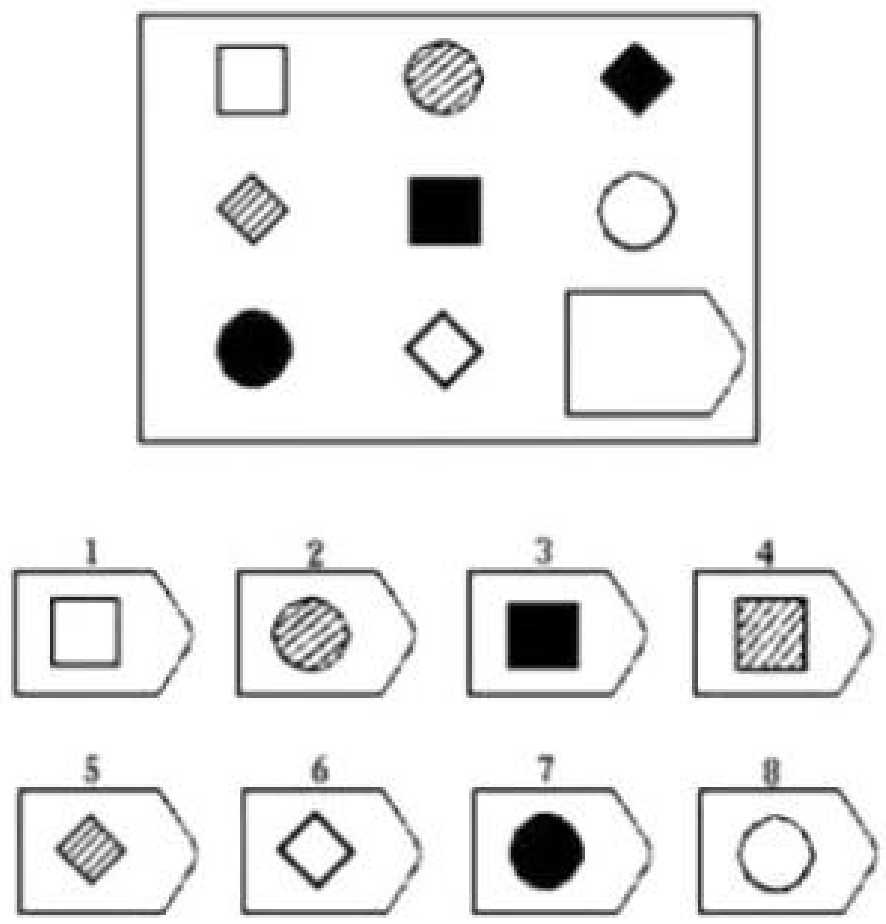

D8

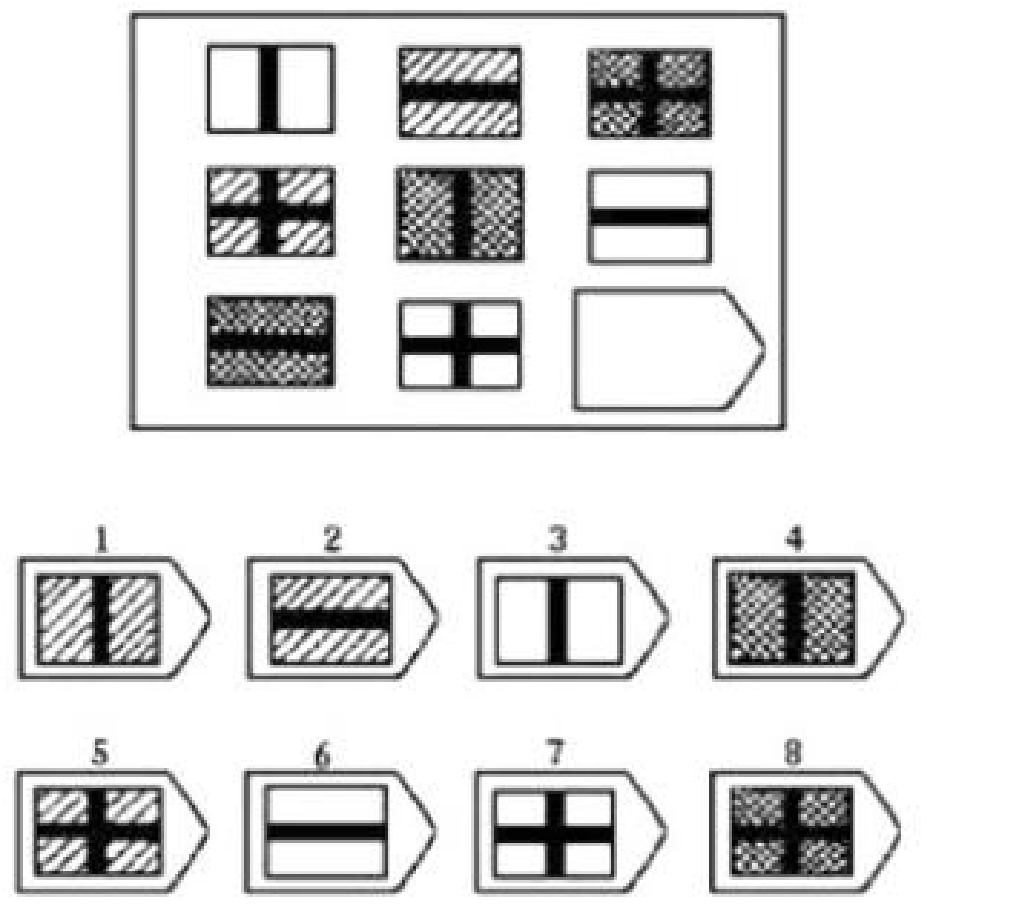

D9

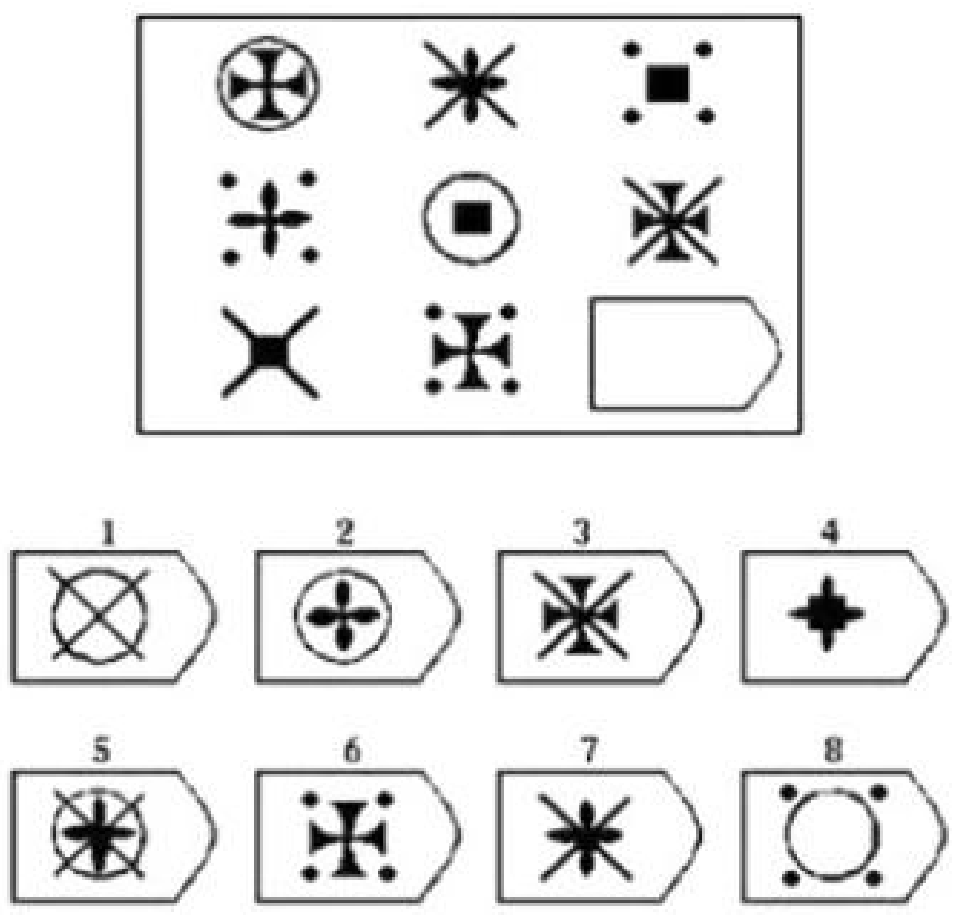

D10

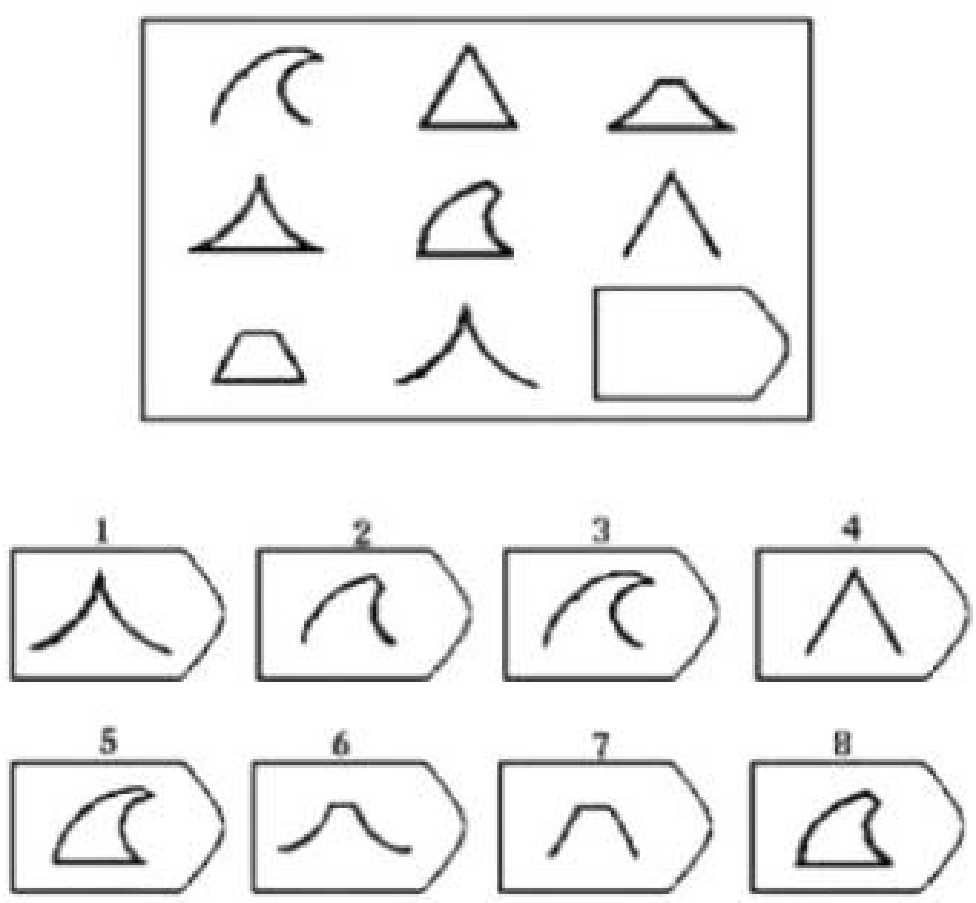

D11

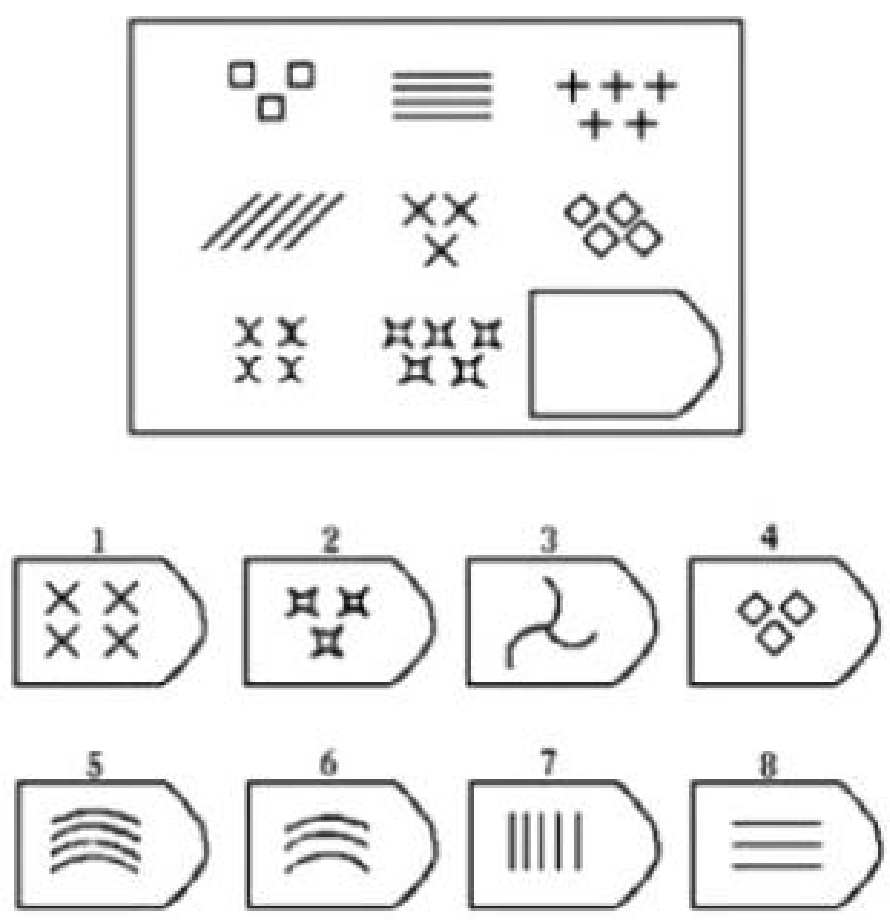

D12

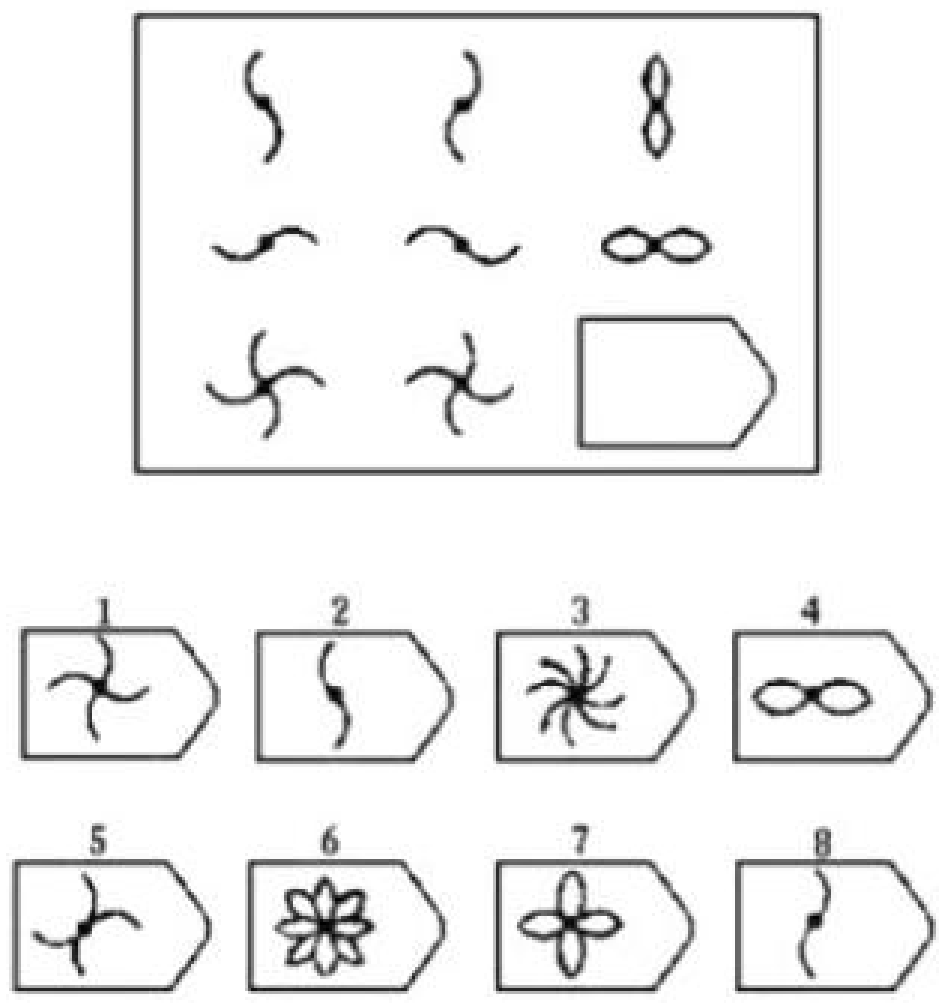

E1

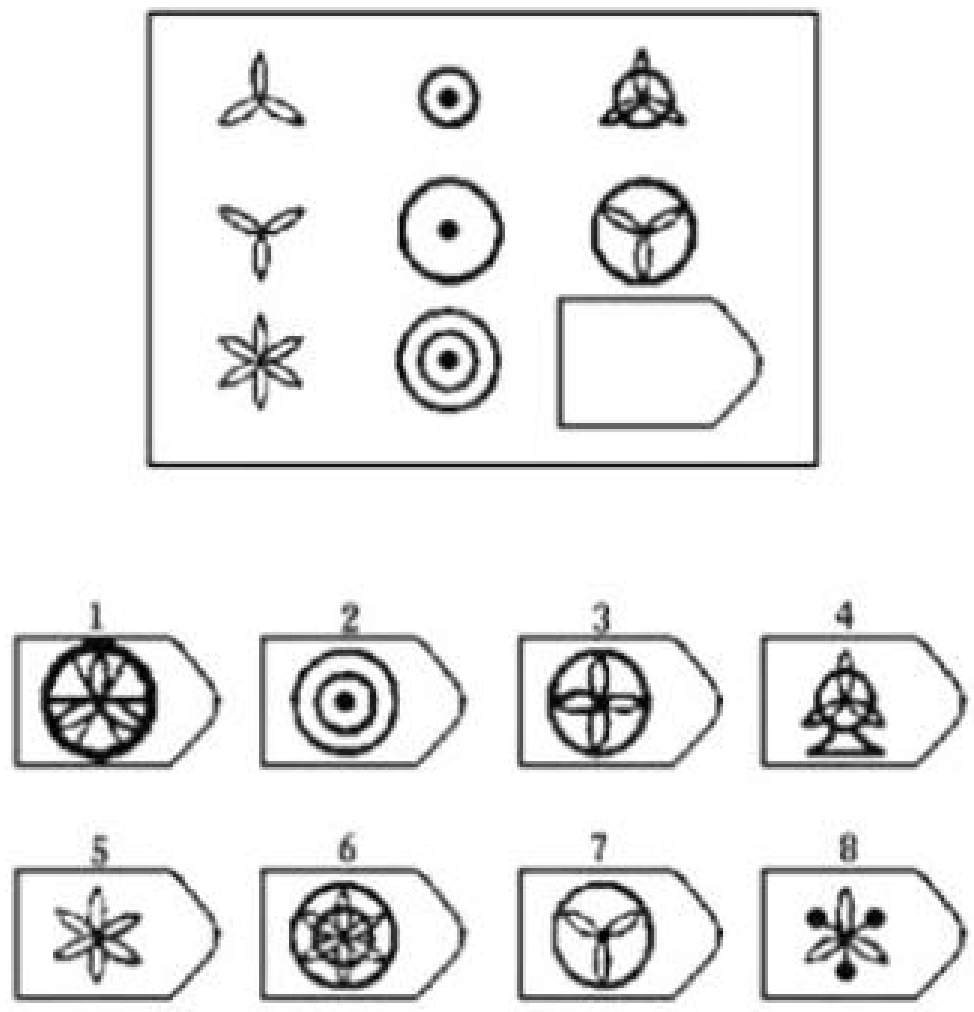

E2

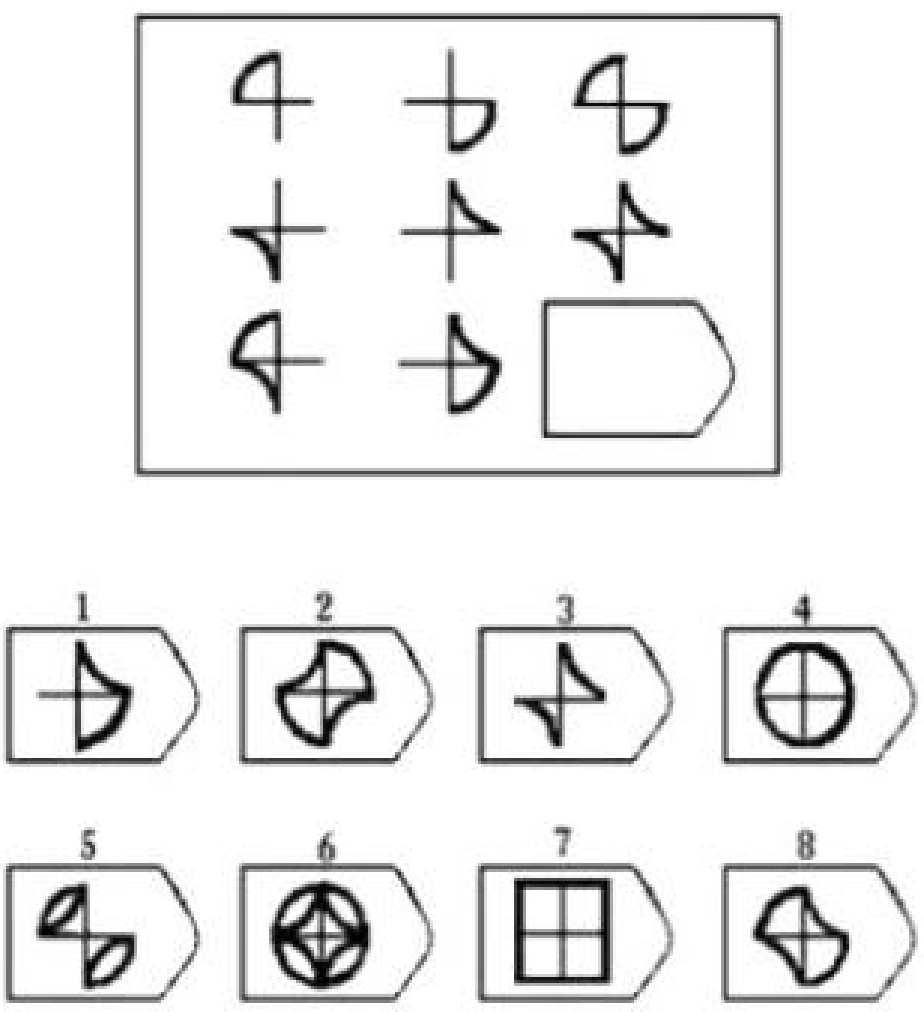

E3

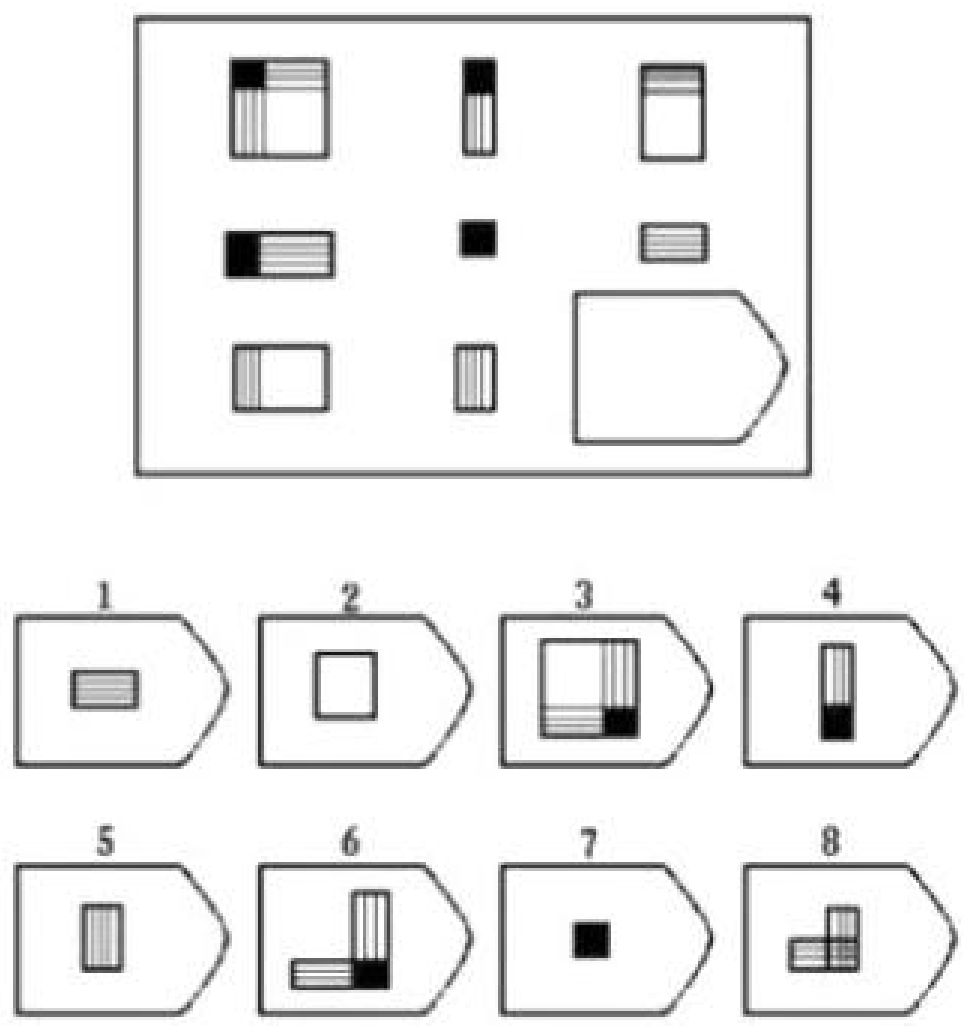

E4

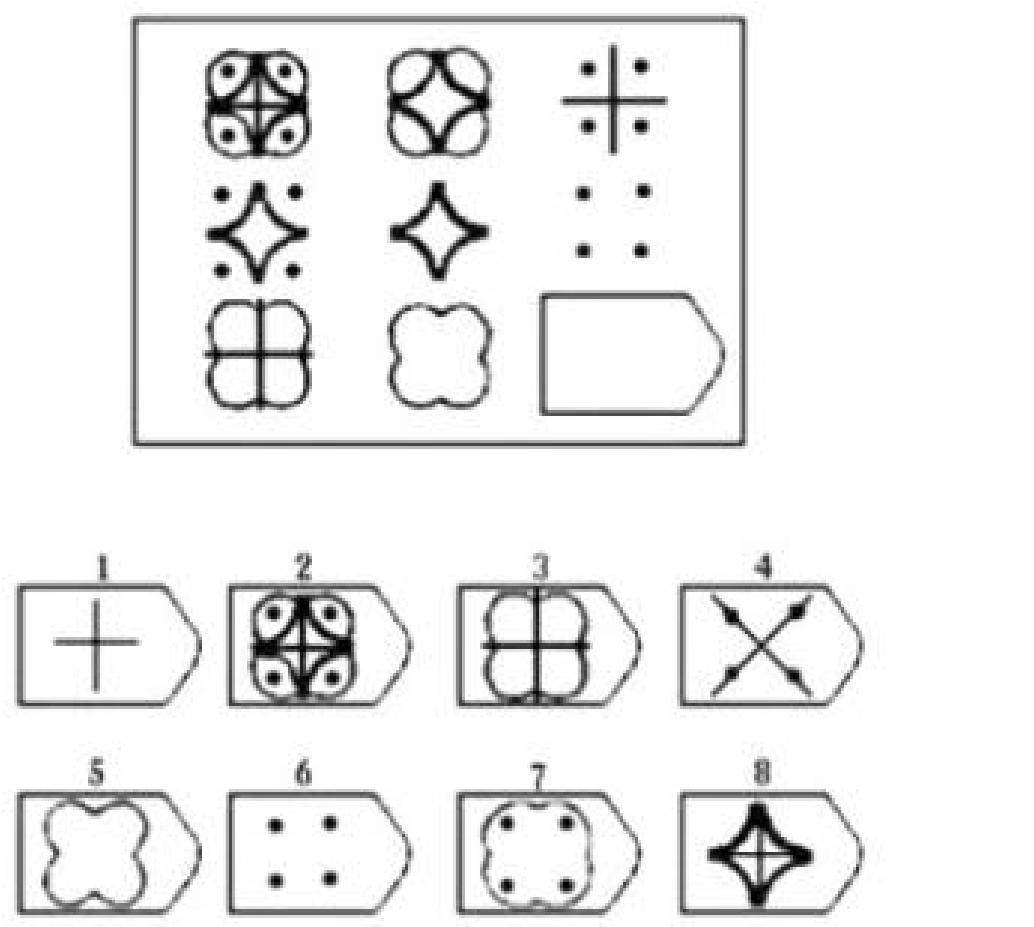

E5

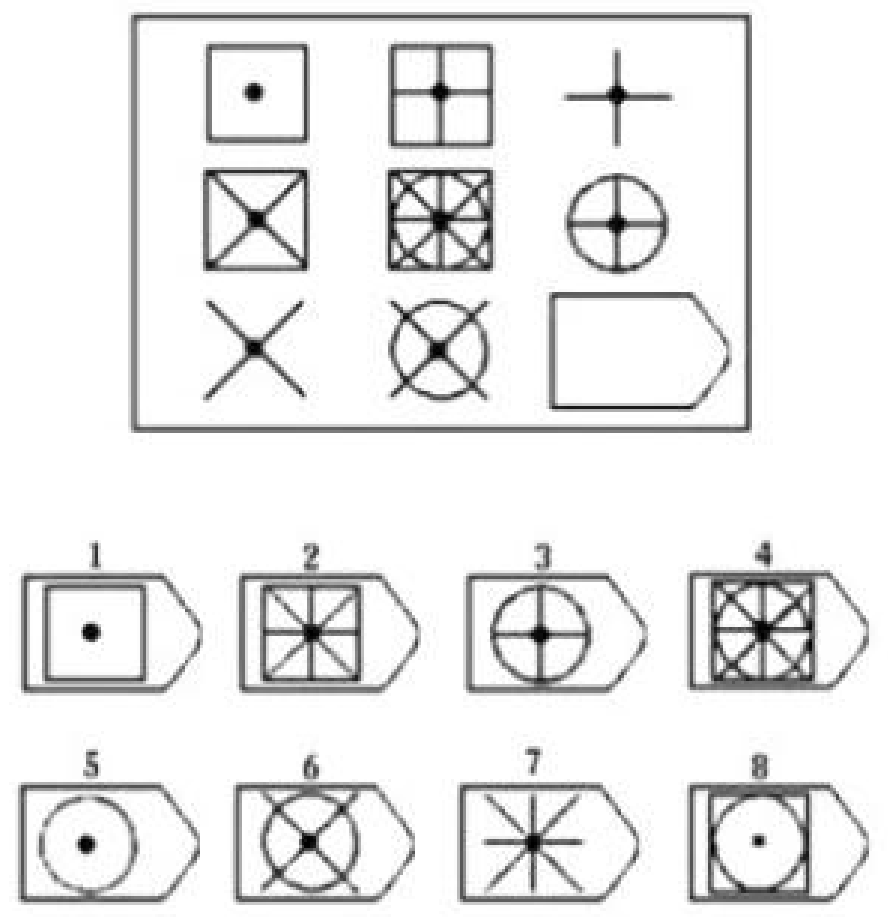

E6

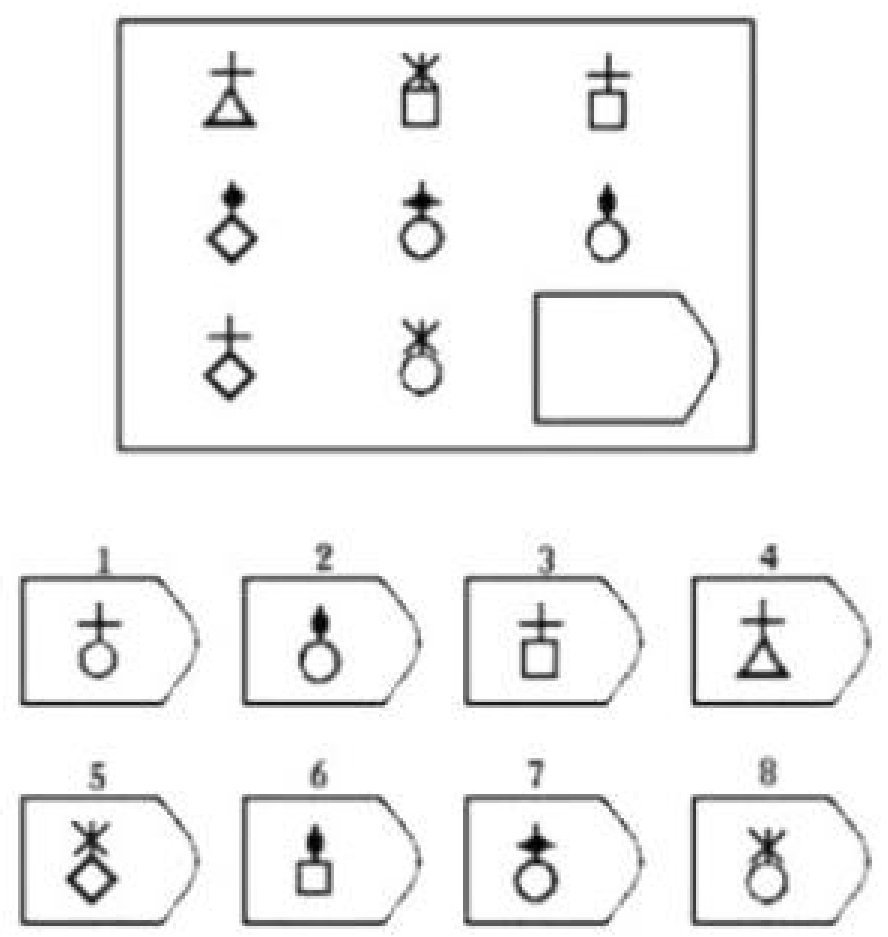

E7

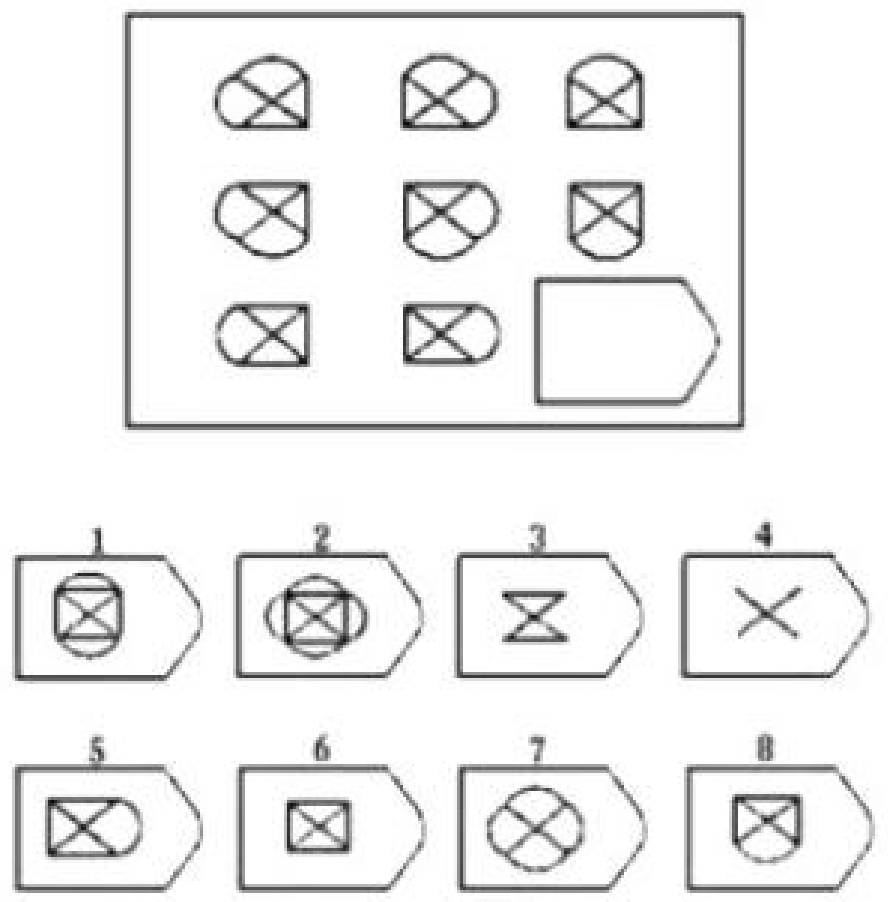

E8

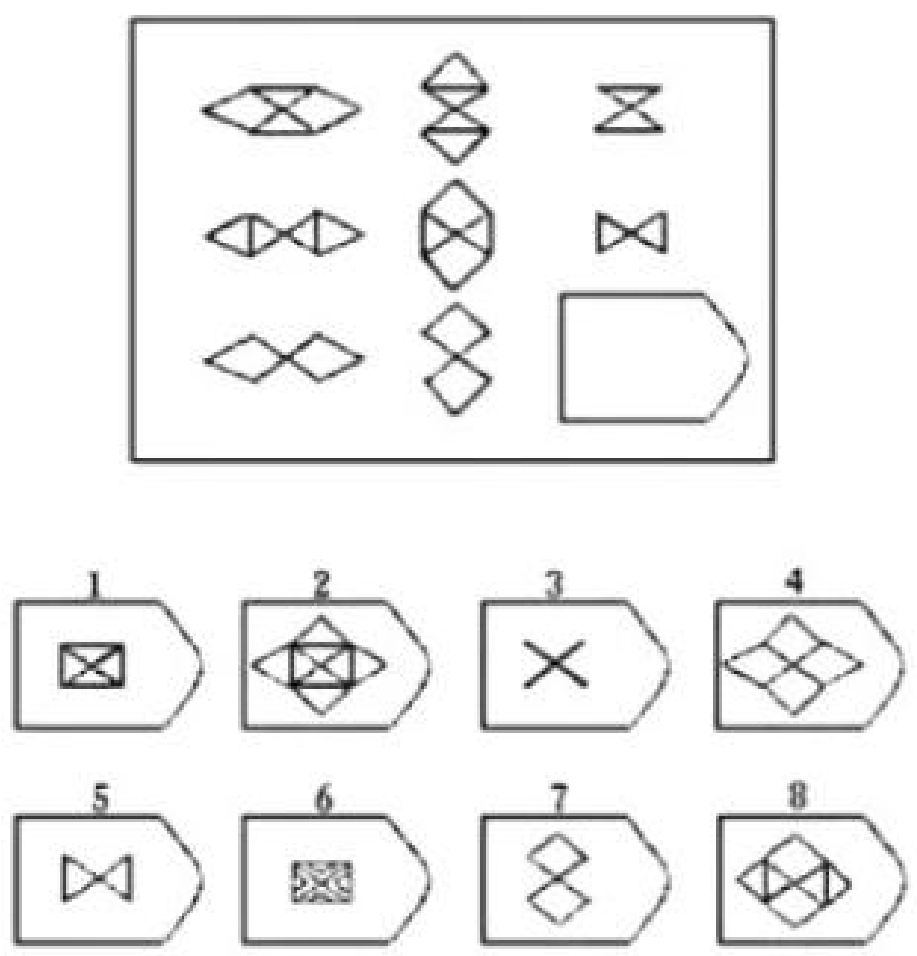

E9

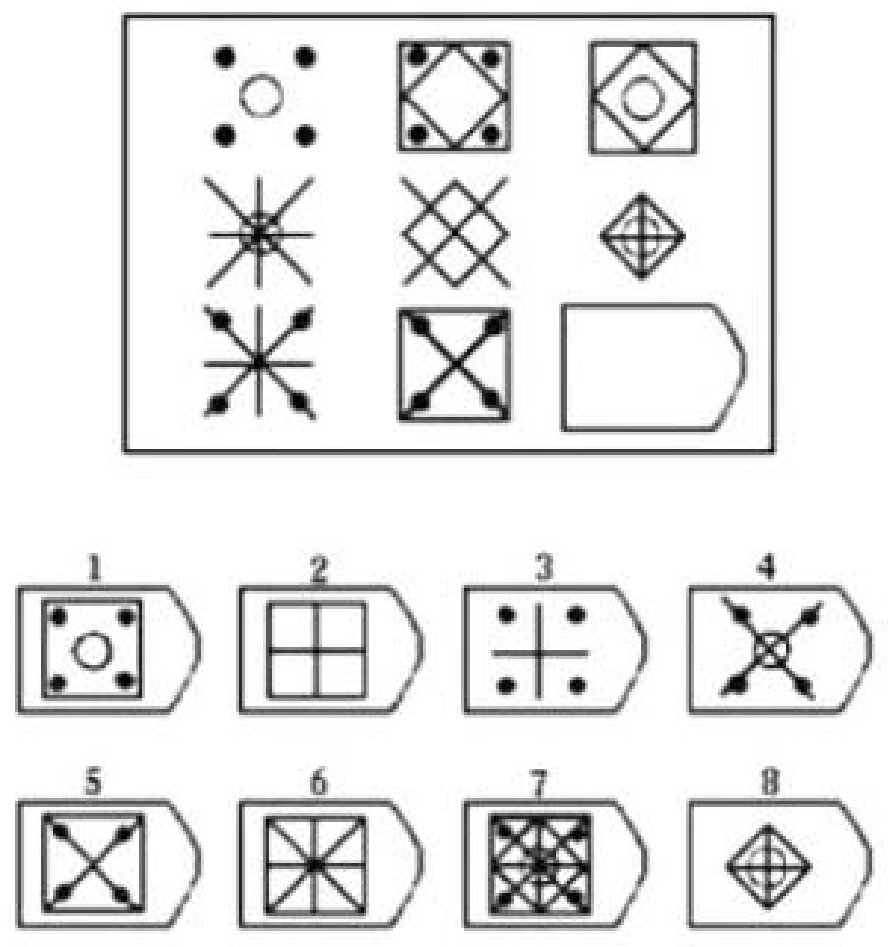

E10

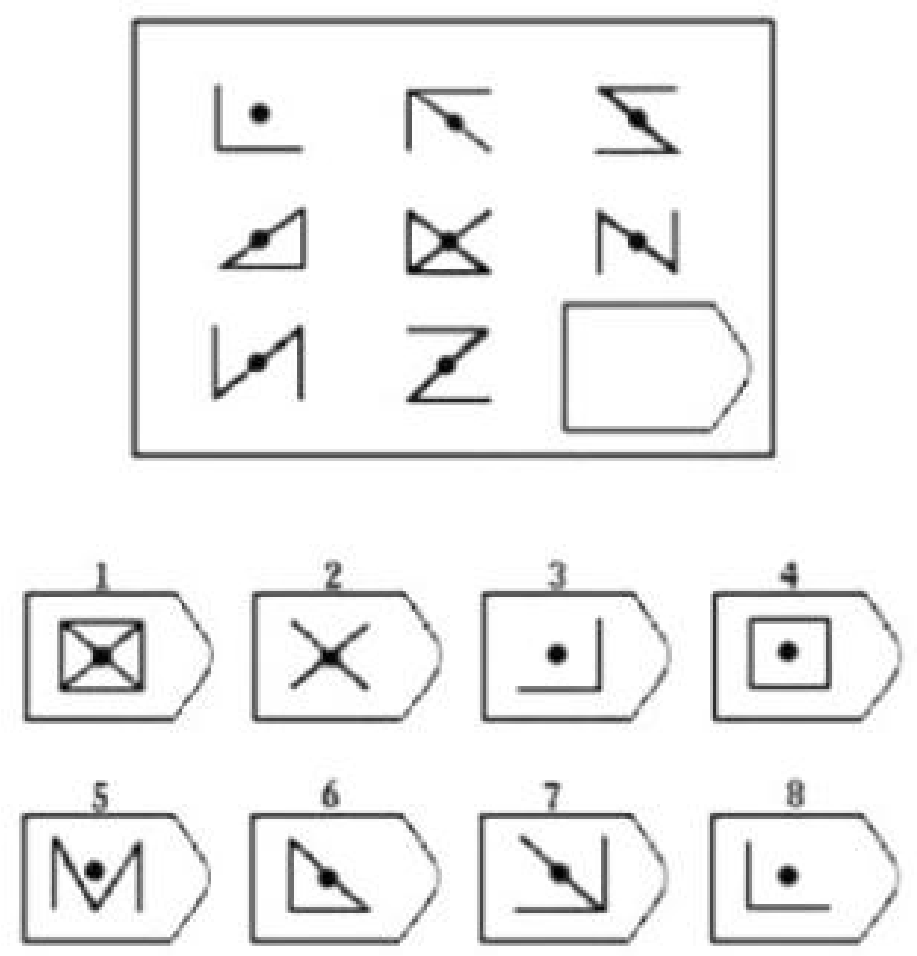

E11

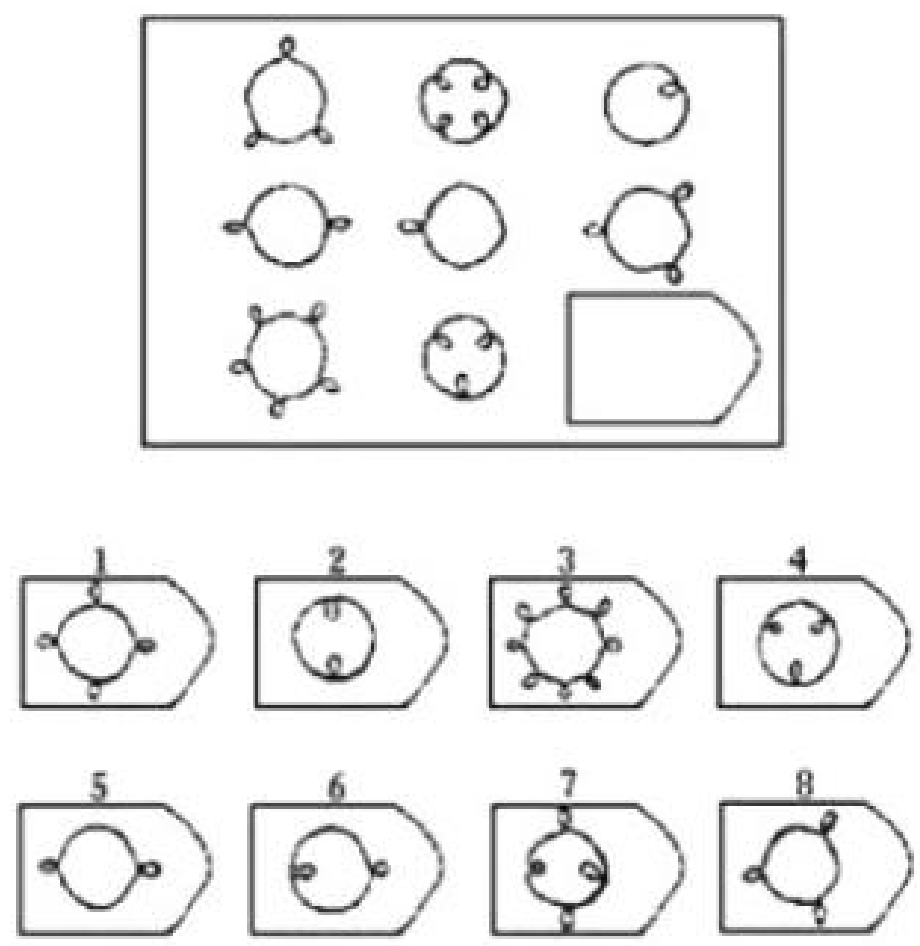

E12
